# Narrowing down a major QTL region reveals *Phytochrome E* (*PHYE*) as the candidate gene controlling flowering time in mungbean (*Vigna radiata*)

**DOI:** 10.1270/jsbbs.23036

**Published:** 2024-02-29

**Authors:** Kitiya Amkul, Kularb Laosatit, Yun Lin, Tarika Yimram, Jingbin Chen, Xingxing Yuan, Xin Chen, Prakit Somta

**Affiliations:** 1 Department of Agronomy, Faculty of Agriculture at Kamphaeng Saen, Kasetsart University, Kamphaeng Saen Campus, Nakhon Pathom 73140, Thailand; 2 Institute of Crop Sciences, Jiangsu Academy of Agricultural Sciences, Nanjing 210014, China

**Keywords:** mungbean, flowering, phytochrome E, QTL, *Vigna radiata*

## Abstract

Flowering time is an important agronomic trait that is highly correlated with plant height, maturity time and yield in mungbean. Up to present, however, molecular basis of flowering time in mungbean is poorly understood. Previous studies demonstrated that flowering time in mungbean is largely controlled by a major QTL on linkage group 2 (LG2). In this study, the QTL on the LG2 in mungbean was investigated using F_2_ and F_2:3_ populations derived from a cross between mungbean cultivar Kamphaeng Saen 2 (KPS2) and wild mungbean accession ACC41. The QTL was narrowed down to a genome region of 164.87 Kb containing a phytochrome gene, designated *VrPHYE*, encoding phytochrome E (phyE), a known photoreceptor modulating flowering time. Compared to *VrPHYE* of the wild ACC41, *VrPHYE* of KPS2 contained several single nucleotide polymorphisms (SNPs) causing amino acid changes. Those SNPs were also found in other mungbean cultivars. Some amino acid changes were predicted to occur in the regulatory region of phytochromes. Gene expression analysis revealed that *VrPHYE* in KPS2 was expressed significantly higher than that in ACC41. These results showed that *VrPHYE* is the candidate gene controlling flowering time in the mungbean.

## Introduction

Mungbean (*Vigna radiata* (L.) R. Wilczek var. *radiata*) is an ancient and important legume crop of tropical and subtropical regions. The crop is domesticated from its wild form, *Vigna radiata* var. *sublobata* (Roxb.) Vercourt, in India about 4,000–4,500 years ago (Fuller and Harvey 2006). After domestication, mungbean has been spread into East, West and Southeast Asia via silk road (Tomooka *et al.* 2000). At present, mungbean is widely grown and consumed in Asia and is now gaining popularity for cultivation and consumption in several regions of Africa, America and Australia. India is the largest producer of mungbean, followed by Myanmar and China with the cultivation area of about 4.5 (Anonymous 2021), 1.2 (MAOLI 2019) and 0.8 (Nair and Schreinemachers 2020) million hectares, respectively. Popularity of the mungbean cultivation is due to the facts that the crop has a short life cycle of only about 55–75 days and ability to fix atmospheric nitrogen to the soil, use less water than other crops, and is relatively drought tolerance (Fernandez and Shanmugasundaram 1988). The crop fits well with several cropping systems, especially cereal-based system. For example, in Thailand, the cultivation of mungbeans starts after the cultivation of rice and maize finish. Mungbean is mainly grown for dry seeds. Dry seeds of mungbean contain high protein (~20–25%) and carbohydrate (~65–75%) (Somta and Srinives 2007). The seeds are used to prepared several foods and processed into sprouts, noodles, starch, splited beans, egg substitutes, and plant-based meats. Phytochemicals presented in the mungbean seeds are believed to have detoxification activity that can refresh the mind, alleviate heat stroke, and reduce swelling in summer (Tang *et al.* 2014).

Flowering time is a key phenological trait of plant adaptation and reproduction. In general, flowering time is largely influenced by photoperiod and temperature (Song *et al.* 2013). In crop plants, flowering time is an important agronomic trait that is highly correlated with plant height, maturity time and yield. Therefore, flowering time is correlated with crop management and harvest. Mungbean is a tropical and quantitative short-day plant (Summerfield and Lawn 1987). Therefore, knowledge and understanding on the molecular genetic mechanism underlying the flowering time in mungbean is crucial for expanding mungbean production to new regions. There are some reports on quantitative trait loci (QTLs) and candidate genes controlling flowering in the mungbean. However, most of the QTLs or candidate genes identified for flowering time are different and have not been confirmed/validated (reviewed in Somta *et al.* 2022). In the previous studies by Isemura *et al.* (2012), Kajonphol *et al.* (2012), and Somta *et al.* (2015), major QTLs controlling flowering time were always detected on linkage group 2 (LG2) and they are possibly the same locus (reviewed in Somta *et al.* 2022). Kajonphol *et al.* (2012) identified four QTLs on linkage group (LG) 2, 4, and 11 for days to first flowering (DFF) in an F_2_ population grown during long-day (LD) and short-day (SD) conditions in Thailand. The QTL *Fld2* on LG2 and *Fld4.2* on LG4 were the major QTLs explaining for 15.9 and 28.6% of the flowering time variation, respectively. Isemura *et al.* (2012) identified four QTLs, one each on LGs 2, 4, 6, and 11 in an F_2_ population (wild × cultivated) grown under LD and SD conditions in Japan where the natural day length gradually decreased from 14 h 12 min in July to 9 h 43 min in December. The *Fld5.2.1-* on LG2 and *Fld5.4.1-* on LG4 had the largest effect with the phenotypic variation explained by the QTL (coefficient of determination (*R*^2^)) of 32.9% and 24.0%, respectively. Somta *et al.* (2015) detected seven QTLs for DFF in F_2:3_ population grown in SD and LD conditions and F_2:4_ population grown in SD condition. Five QTLs, two on LG2 and one each on LGs 4, 5, and 6, were associated with LD condition, while two QTLs on LG2 were associated with SD condition. The QTLs *qDFL2.1* and *qDFL2.2* were detected in both conditions and expressed similar effect with *R*^2^ of about 29.0%. The QTLs on LGs 2, 4, 6, and 11 were always detected, especially those on LGs 2 and 4. So, the results in these reports showed that QTLs on LG2 were always detected in both the SD and LD conditions, while the QTLs on LGs 4, 6, and 11 were always identified in the LD condition.

The QTLs on LG2 appeared to be the major genetic factors playing important role in regulating flowering time in mungbean. Nonetheless, marker density of the linkage maps used in the studies of Isemura *et al.* (2012), Kajonphol *et al.* (2012), and Somta *et al.* (2015) are sparse and, up to present, molecular basis of the flowering QTLs on the LG2 has not yet been investigated. In this study, we report fine mapping of the QTL on LG2 and identification of candidate gene controlling flowering time in the mungbean.

## Materials and Methods

### Population development and DNA extraction

Two mapping populations, F_2_ and F_2:3_ population, were used in this study. These populations were derived from a cross between Kamphaeng Saen 2 (hereafter called KPS2) and ACC41. KPS2 is a commercial mungbean cultivar of Thailand. It is developed by Kasetsart University, Thailand. ACC41 is a wild mungbean originated from Australia. The seeds of ACC41 were obtained from Australian Tropical Crops and Forages Genetic Resources Centre, Australia. The F_2_ population comprised 575 individuals. The F_2_ population and 10 plants of each parent were grown under a field condition at Kasetsart University, Kamphaeng Saen Campus, Nakhon Pathom, Thailand from February to May 2018. Twenty-five days after planting, days to first flowering of each plant was observed and recorded. DNA extraction was performed following the procedures reported by Lodhi *et al.* (1994). Details of the F_2:3_ generation is described in the section “Confirmation of the QTL for flowering time”. It is noteworthy that the F_2_ population used in this study was the same population used to identify the gene for seed dormancy previously reported by Laosatit *et al.* (2022).

### Bioinformatics analysis of the mungbean QTLs on LG2 controlling flowering time and development of new markers for fine mapping

Since Somta *et al.* (2015) reported that the *qDFL2.1* and *qDFL2.2* on LG2 controlling DFF were located between marker intervals GBssr-MB87–DMB-SSR160 and DMB-SSR160–CEDAAG002, respectively, while Kajonphol *et al.* (2012) reported that the QTL *Fld2* on LG2 governing DFF was localized to the marker interval DMB-SSR160–VR0364 and Isemura *et al.* (2012) reported that the *Fld5.2.1-* on LG2 regulating DFF was delimited by the marker interval GMES0477–CEDG026. So, physical locations of the markers GBssr-MB87, DMB-SSR160, CEDAAG002 and CEDG026 on the reference genome sequence of the mungbean cultivar Sulv1 (Yan *et al.* 2020) were determined by BLASTN analysis. Once determined, all the genes in genome region harboring the QTLs for DFF were checked and genes having function(s) involving in the flowering time (Lin *et al.* 2020) were selected. Sequence data of genome region of 800-Kb covering each selected gene was downloaded and identified for simple sequence repeats (SSR) using SSRIT (Temnykh *et al.* 2001). Subsequently, primers were designed to amplify SSRs using Primer3 (Untergasser *et al.* 2012). In addition, each of the 800-Kb region was aligned against the genome sequence of *Vigna* sp. accession NI1135 (https://doi.org/10.1101/2022.03.28.486085), an accession genetically closest to wild mungbean (Takahashi *et al.* 2018), to identify insertions/deletions (InDels). Subsequently, primers for InDels were designed using Primer3 (Untergasser *et al.* 2012).

### DNA marker, linkage and QTL analyses

The primers (Supplemental Table 1) were screened for polymorphism between KPS2 and ACC41. DNA marker analysis was carried out as per Laosatit *et al.* (2022). Briefly, polymerase chain reaction (PCR) was carried out in a total volume of 10 μl containing 5 ng of DNA template, 1 × *Taq* buffer, 2 mM MgCl_2_, 0.2 mM dNTPs, 1 U *Taq* DNA polymerase (Thermo Fisher Scientific Inc., Waltham, MA, USA), and 2.5 μM each of forward and reverse primers. PCR products were electrophoresed on 5% polyacrylamide gel electrophoresis and visualized by silver staining. Markers showing clear polymorphic DNA bands were used to analyze the F_2_ population.

Linkage map was constructed using software QTL IciMapping 4.2 (Meng *et al.* 2015). Markers were grouped with logarithm of odds (LOD) values of 5.0, and then ordered using REcombination Counting and ORDering (RECORD) method (Van Os *et al.* 2005). Distances between markers were calculated using Kosambi’s mapping function (Kosambi 1943).

QTL controlling DFF was localize onto the linkage map using the inclusive composite interval mapping method (ICIM) (Li *et al.* 2007) by the QTL IciMapping 4.2. A significant LOD threshold for QTL identification was determined by a 10,000 permutation test at *P* = 0.001. DNA marker and DFF data used in the QTL analysis of the F_2_ population were shown in Supplemental Table 2.

### Confirmation of the QTL for flowering time

Once the QTL for flowering time was identified, the locus was confirmed using an F_2:3_ population. This population comprised 217 F_3_ plants that were progenies of 15 F_2_ (KPS2 × ACC41) plants showing heterozygous genotype at the markers flanking the QTL for flowering time. The number of F_3_ plants derived from each F_2_ plant was between 10 and 25. The F_2:3_ population together with 10 plants of each parent were grown in a field condition from February to May 2020 in the same location with the F_2_ population. Data collection for flowering time (DFF), DNA extraction, DNA marker analysis, linkage analysis and QTL analysis of the F_2:3_ population were the same as described for the F_2_ population. DNA marker and DFF data used in the QTL analysis of this population were shown in Supplemental Table 3.

### Sequence and RT-qPCR analyses of candidate gene

Based on the result from fine mapping, the gene *EVM0010407* (*VrPHYE*) was selected as the single candidate gene controlling the flowering time. A genomic region harboring the gene *VrPHYE* in KPS2 and ACC41 was amplified using primers listed in the Supplemental Table 1. PCR was conducted as described above. The PCR products were electrophoresis in 1.0% agarose gel to confirm whether a single band is amplified. Then, the products were eluted from the gel, cleaned and sequenced. Sanger sequencing was performed using ABI 3730xl DNA Analyzer (Applied Biosystems, CA, USA) by AGTC Genomics (Kuala Lumpur, Malaysia). The *VrPHYE* sequences from KPS2 and ACC41 were aligned with the reference sequences of the mungbean cultivar Sulv1 (Yan *et al.* 2020), Jilv7 (Liu *et al.* 2022) and VC1973A (Ha *et al.* 2021) using Clustal Omega (Sievers *et al.* 2011). Predicted protein sequence encoded by the *VrPHYE* in all the mungbean cultivars were also aligned. The protein sequence was also subjected to InterProScan (Paysan-Lafosse *et al.* 2023) to identify protein domain(s).

Expression of the *VrPHYE* in KPS2 and ACC41 was detected and quantified by quantitative reverse transcription PCR (RT-qPCR). KPS2 and ACC41 were grown in a crossing block. At 35 and 45 days after planting (DAP), total RNA was extracted from leaves of both accessions. RNA extraction and complementary DNA (cDNA) synthesis were conducted as described by Laosatit *et al.* (2022). Primers for RT-qPCR of the *VrPHYE* and reference gene *VrACTIN* (*LOC106770112*) were listed in the Supplemental Table 1. The RT-qPCR was performed with three biological and technical replicates were conducted using ViiA 7 Real-Time PCR System (Applied Biosystems). PCR reaction mixtures and thermal cycle conditions were the same as described by Laosatit *et al.* (2022). Expression levels of the gene *EVM0010407* were calculated based on the 2^–ΔΔCT^ method (Livak and Schmittgen 2001). Statistical difference in the gene expression between KPS2 and ACC41 was determined by t-test at 5% probability using R-program. The expression of the *VrPHYE* gene was detected at only 35 and 45 DAP because at later stages KPS2 was at maturing and harvesting stages and the leaves were old, while ACC41 was still at the vegetative stage or may be at the beginning of the flowering initiation, and thus comparison at the later stages was not suitable.

### Phylogenetic analysis

The predicted proteins encoded by the *VrPHYE* in the mungbean together with their homologous proteins from other plants including azuki bean (*Vigna angularis* (Ohwi) Ohwi and Ohashi), black gram (*Vigna mungo* (L.) Hepper), cowpea (*Vigna unguiculata* (L.) Walp.), common bean (*Phaseolus vulagaris* L.), lima bean (*Phaseolus lunatus* L.), lablab (Lablab *purpureus* (L.) Sweet), soybean (*Glycine max* (L.) Merr.), wild soybean (*Glycine soja* Siebold and Zucc.), groundnut (*Arachis hypogaea* L.), wild groundnut (*Arachis duraensis* L.), pigeon pea (*Cajanus cajan* (L.) Millsp.), chickpea (*Cicer arietinum* L.), barrel medic (*Medicago truncatula* Gaertn.), *Lotus japonicus* (Regel) K. Larsen and subterranean clover (*Trifolium subterraneum* L.) were subjected to phylogenetic analysis using MEGA 11 (Tamura *et al.* 2021). The sequences were aligned using MUSCLE and the phylogenetic tree was constructed using maximum likelihood method with 1,000 bootstraps. Details of the protein sequences used in the phylogenic analysis are shown in Supplemental Table 4.

## Results

### Physical location of the mungbean QTLs on LG2 controlling flowering time

Previous studies showed that major QTLs (*qDFL2.1*, *qDFL2.2*, *Fld*, and *Fld5.2.1-*) controlling mungbean flowering time are on LG2, but location(s) of those QTLs on the mungbean reference genome is not known. In this study, bioinformatics analysis showed that markers GBssr-MB87, DMB-SSR160, CEDAAG002 and CEDG026 associating with the major QTL(s) for flowering time located between the positions 36.172 and 42.480 Mb on the chromosome 4 of the Sulv1 genome. So, the *qDFL2.1*, *qDFL2.2*, *Fld*, and *Fld5.2.1-* controlling the flowering time were believed to reside in this region. We checked all the genes in the region focusing on the one(s) with annotated function relating to flowering time. Four flowering-related genes were identified including *EVM0010407*, *EVM0000157*, *EVM0026933* and *EVM0031852*. *EVM0010407* and *EVM0000157* were annotated to encode phytochrome E (phyE) and agamous-like MADS-box protein AGL82 (AGL82), respectively, while both *EVM0026933* and *EVM0031852* were annotated to encode clock-associated PAS protein ZEITLUPE (ZTL). These genes located at the positions 39.700, 39.128, 42.035, and 42.041 Mb, respectively.

### QTL region controlling flowering and identification of candidate gene

**Fig. 1.**
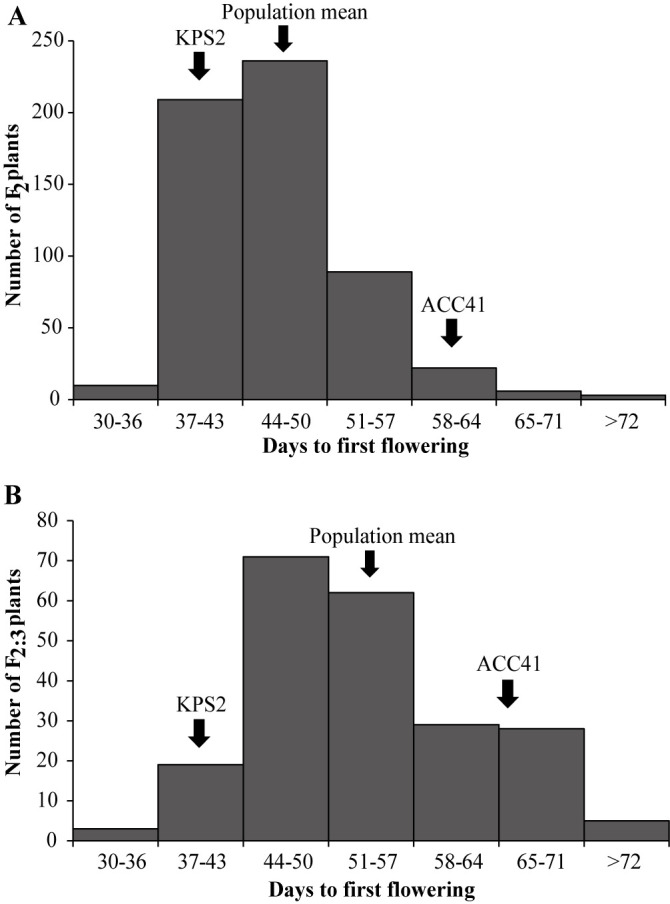
Frequency distribution of days to first flowering in F_2_ (A) and F_2:3_ (B) populations derived from a cross between cultivated mungbean Kamphaeng Saen 2 (KPS2) and wild mungbean ACC41.

The QTL(s) controlling flowering time appeared to locate on region between 36.172 and 42.480 Mb on the chromosome 4 of the Sulv1 mungbean reference sequence. In order to finely mapped the QTLs on LG2, SSR and InDel markers were developed from the 38.00–43.00 Mb region. In total, 83 SSR and 64 InDel markers were developed (Supplemental Table 1). We narrowed down the QTL region using an F_2_ population derived from parents showing contrasting flowering time (days to first flowering (DFF)). DFF in the F_2_ population varied between 32 and 80 days with a mean of 46.2 days. DFF in the mapping parents KPS2 and ACC41 was of 38.2 and 64.0 days, respectively. DFF in the F_2_ population showed continuous distribution (Fig. 1A).

**Fig. 2.**
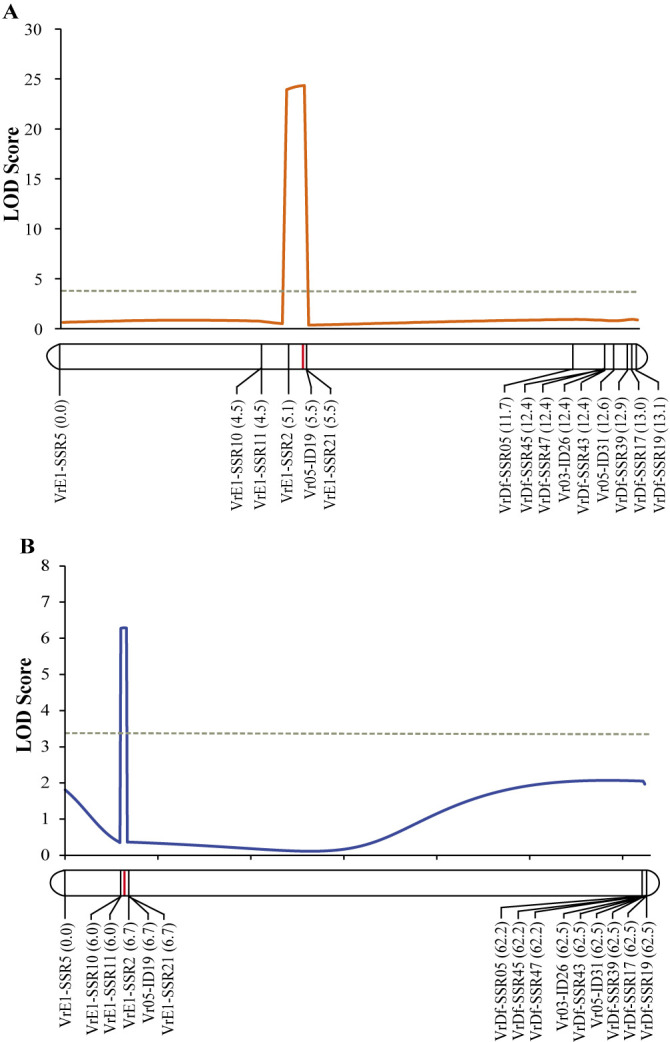
LOD graph of *qFld2.1* on linkage group 2 controlling days to first flowering in F_2_ (A) and F_2:3_ (B) populations derived from a cross between cultivated mungbean Kamphaeng Saen 2 (KPS2) and wild mungbean ACC41.

Screening of 147 markers in the parents revealed that 57 showed polymorphisms (Supplemental Table 1). Fifteen markers were selected and used to genotype the F_2_ population. Linkage analysis of the LG2 for the F_2_ population showed that the LG2 spanned 13.13 cM in length with average distance between adjacent markers of 0.94 cM. QTL analysis by ICIM method identified a single QTL controlling the days to first flowering (Fig. 2A, Table 1). The QTL was mapped between the markers VrE1-SSR2 and Vr05-ID19. It accounted for 17.79% of the total variation of the days to first flowering in the F_2_ population and expressed the additive effect of –3.96 and dominant effect of 0.64. The QTL was designated *qFld2.1*.

The *qFld2.1* was confirmed using an F_2:3_ population derived from F_2_ plants. In this population, DFF ranged from between 30 to 75 days with a mean of 53.2 days. DFF in KPS2 was 38.7 days, while that in ACC41 was 64.4 days. DFF in the F_2:3_ population showed continuous distribution (Fig. 1B). ICIM detected a single QTL, *qFld2.1*, for the days to first flowering (Fig. 2B, Table 1). The *qFld2.1* was located between the markers VrE1-SSR11 and VrE1-SSR2. It explained 12.44% of the total variation of the days to first flowering in the F_2:3_ population with the additive and dominant effects of –4.09 and 1.49, respectively.

**Fig. 3.**
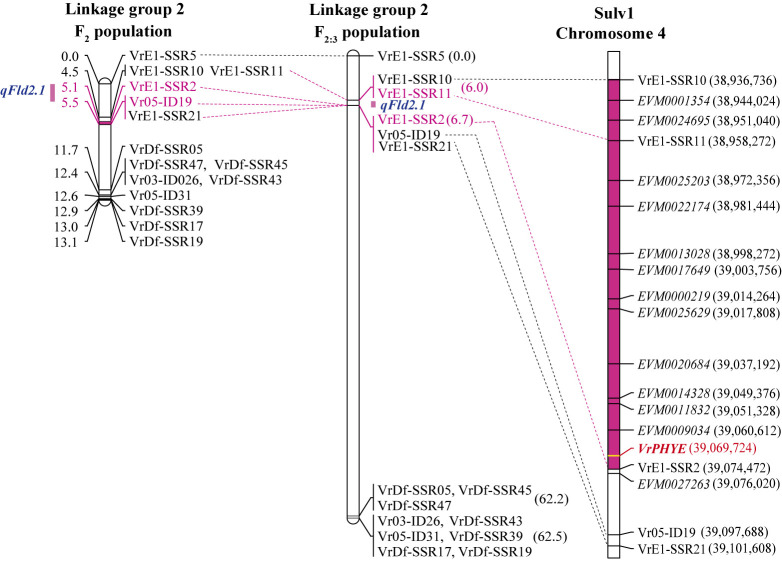
A comparative map showing location of *qFld2.1* detected on linkage group 2 in the F_2_ (left) and F_2:3_ (middle) populations and position of the *qFld2.1* on the reference genome sequence of the mungbean cultivar Sulv1 (left).

Based on the results in the F_2_ and F_2:3_ populations, the location of the *qFld2.1* was in region from the marker VrE1-SSR10 to VrE1-SSR21 with VrE1-SSR2 always the flanking marker to the *qFld2.1*. Candidate gene(s) was identified using the reference genome sequence of mungbean cultivar Sulv1. BLASTN analysis showed that the markers VrE1-SSR10 and VrE1-SSR21 were 164.87 Kb apart, being at the positions 38,936,736 and 39,101,608 on the chromosome 4 (Fig. 3). There were 14 predicted genes in the 164.87-Kb region, including the gene *EVM0010407* (Fig. 3, Supplemental Table 5). So, the gene *EVM0010407*, designated *VrPHYE*, was chosen as the candidate gene at the *qFld5.1* for the days to first flowering.

### *VrPHYE* sequence and expression

**Fig. 4.**
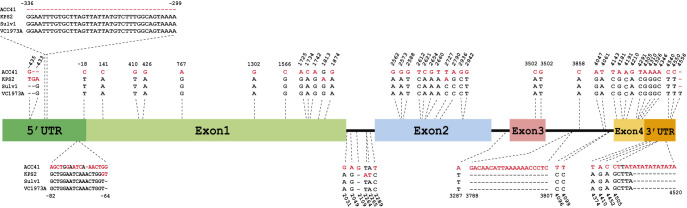
*PHYE* sequence polymorphisms among mungbean accessions ACC41, Kamphaeng Saen 2 (KPS2), Sulv1, VC1973A and Jilv1.

*VrPHYE* in KPS2 and ACC41 was sequenced. The *VrPHYE* sequences of KPS2 and ACC41 were aligned with the Sulv1 reference sequence and VC1973A reference sequence. The alignment revealed that several polymorphisms in 5ʹ untranslated region (UTR), exons, introns, and 3ʹ UTR (Fig. 4). Most of the polymorphisms discriminated between the cultivated (KPS2, Sulv1, Jilv7 and VC1973A) and wild (ACC41) mungbeans. Nonetheless, coding sequence (CDS) comparison between the mapping parents (KPS2 and ACC41) revealed 31 single nucleotide polymorphisms (SNPs). In addition, 5ʹ UTR sequence comparison between ACC41 and KPS2 showed 15 SNPs and 2 InDels (Supplemental Fig. 1). One of the InDel was a 38 bp (insertion in KPS2). This insertion also existed in Sulv1, Jilv7, and VC1973A.

**Fig. 5.**
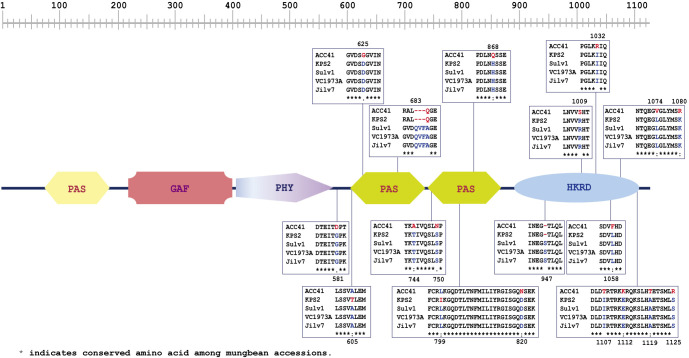
*VrPhyE* sequence polymorphisms among mungbean accessions ACC41, Kamphaeng Saen 2 (KPS2), Sulv1, VC1973A and Jilv1. Protein domains of the PhyE are also shown. PAS = Period/Arnt/Single-Minded domain, GAF = cGMP phosphodiesterase/adenylyl cyclase/FhlA domain, PHY = phytochrome-specificdomain and HKRD = histidine kinase-related domain.

VrPhyE protein sequence alignment among ACC41, KPS2, Jilv7, Sulv1 and VC1973A revealed the ACC41 and KPS2 had the same length of 1,121 amino acids and differed from Sulv1, Jilv7 and VC1973A which had the same length (1,125 amino acids) and sequence (Fig. 5). There were several amino acid changes between the wild mungbean ACC41 and all the cultivated mungbeans. Comparison between ACC41 and KPS2 revealed 17 amino acid changes. Nearly all of these amino acid changes were also found in other cultivated mungbeans. Based on InterProScan, the VrPHYE was composed of 6 protein domains including 3 Period/Arnt/Single-Minded (PAS), a cGMP phosphodiesterase/adenylyl cyclase/FhlA (GAF), a phytochrome-specific (PHY) domain and a histidine kinase-related domain (HKRD) domain (Fig. 5). All of the 17 amino acid changes between ACC41 and KPS2 existed in the C-terminal region: 7 and 9 of the changes occurred in the PAS and HKRD domains, respectively (Fig. 5).

**Fig. 6.**
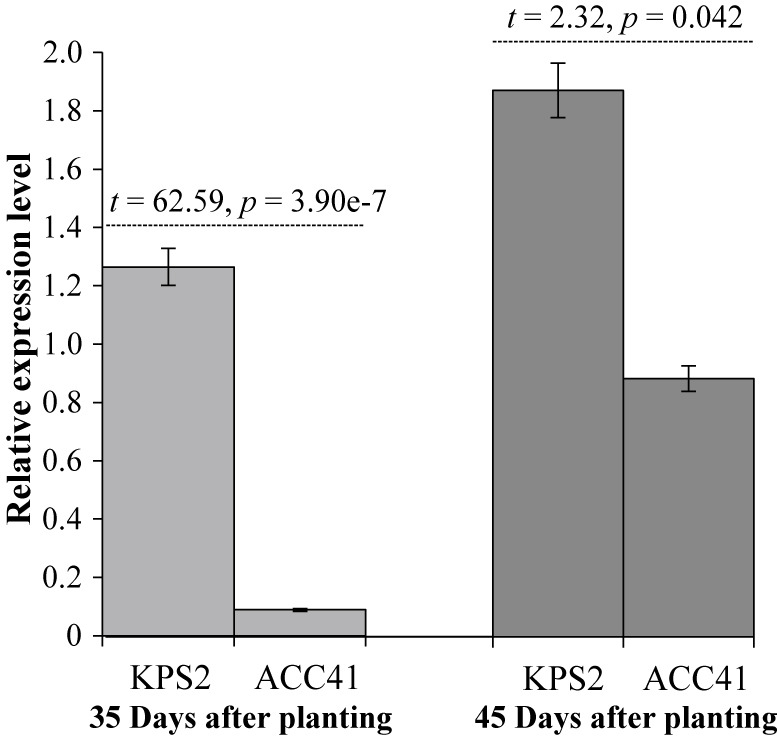
Expression level of *VrPHYE* in leaves of Kamphaeng Saen 2 (KPS2) and ACC41 at 35 (A) and 45 (B) days after planting. Error bars are standard error.

RT-qPCR analysis showed that expression level of the *VrPHYE* in KPS2 was significantly higher than that in ACC41 at both 35 and 45 DAP (Fig. 6), being 14.2 and 2.1 times higher than ACC41, respectively.

### Phylogeny of phyE protein in legume crops

**Fig. 7.**
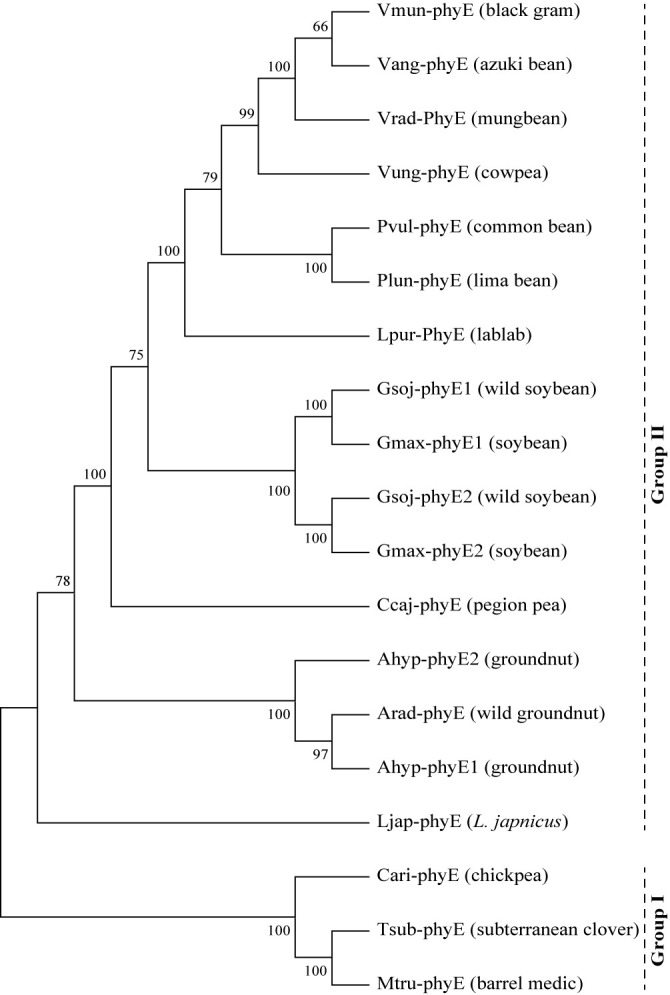
A phylogenic tree depicting relationship of phytochrome E proteins from different legume species. The tree is constructed by maximum likelihood method.

A phylogenic tree constructed from the phyE proteins from different legumes showed that the proteins are largely clustered into two groups, I and II (Fig. 7). Group I comprised phyE proteins from chickpea, subterranean cover and barrel medic, while group II comprised protein from mungbean and the other legumes. phyE from mungbean was closely related with those from black gram, azuki bean and cowpea. All the legume species in the group I were long-day plants, while all the species in the group II except *Lotus japonicus* were short-day plants.

## Discussion

Flowering time is an important agronomic and adaptive trait in crop production, and is therefore a major selection criterion in plant breeding. Usually, wild progenitor(s) and landrace cultivars of tropical crops are short-day plants showing high degree of sensitivity to photoperiod and causing them their flowering is progressively delayed as the photoperiod is increased. Mungbean is a quantitative short-day plant that time to flowering varies appreciably depending on the genotype, photoperiods and temperatures prevailing during the period after sowing (Summerfield and Lawn 1987, Vas Aggarwal and Poehlman 1977). However, mungbean is probably the most short-duration field crop that many improved cultivars mature and can be reaped within 55–65 days after planting (Fernandez and Shanmugasundaram 1988), making the crop useful for diversification in cropping systems. Therefore, understanding and maintaining reduced sensitivity to photoperiod and temperature is important in mungbean breeding. Although QTLs for flowering time have been identified in the mungbean, very little is known about molecular genetic architecture of flowering time in this crop. In this study, we narrowed down the major QTL on LG2 controlling the flowering time in the mungbean from a region of 6.31 Mb to a region of 164.87 Kb (Fig. 3) and showed that *VrPHYE* encoding phytochrome E (phyE) is the candidate gene for the flowering time in the mungbean (Figs. 3–6). In land plants, phytochromes are photoreceptors of red and far-red light that are responsible for triggering responses to specific light signals. The phytochromes play roles in germination, de-etiolation, shade avoidance, and flowering of plants (Legris *et al.* 2019). Five phytochromes have been identified in plants, phyA to phyE (Clack *et al.* 1994, Rockwell *et al.* 2006). Based on phylogenetic analysis in *Arabidopsis thaliana* L., the phytochromes are grouped into three classes, viz. phyA, phyB and phyC in which the phyB class is composed of phyB, phyD and phyE (Clack *et al.* 1994). Based on BLASTP analysis using *A. thaliana* phytochrome protein sequences as queries, genome of mungbean cultivar Sulv1 (Yan *et al.* 2020) contained four phytochrome genes including two *phyA* (*EVM0031724* and *EVM0012118*), one *phyB* (*EVM0002707*) and one *phyE* genes (P. Somta, unpublished data). In *A. thaliana*, *phye* single mutants showed the same phenotypes with wild-type plants but showed earlier flowering in a *phya phyb* background, indicating that the function of phyE overlaps with that of phyA and phyB to delay flowering (Devlin *et al.* 1998). Later, again in *A. thaliana*, Clack *et al.* (2009) demonstrated that (1) phyE do not homodimerize, (2) heterodimeric phytochromes containing phyE or phyC play roles in light regulation of flowering time, (3) in the obligate heterodimerization of phyE and phyC, the latter become unstable by removal of its phyB binding partner, and (4) under SD condition, *phyE* and *phyB* mutants flower earlier than the wild type, and combination of these two mutants exacerbates early flowering. They further illustrated that in the absence of its phyB and phyD dimerization partners, phyE has biological activity. Recently, however, Sanchez-Lamas *et al.* (2016) showed that none of the phytochromes alone conferred a photoperiodic response and phyE and phyB are the strongest repressor of flowering time in *A. thaliana* and the repression is highly dependent on ambient temperature under long-day conditions in *A. thaliana*, albeit both phyB and phyC are needed to confer flowering response to photoperiod. Shapulatov *et al.* (2021) demonstrated that upregulation of both PHYA and PHYB under far-red light is dependent on PHYE. A recent study in soybean demonstrated that *PHYE1* (*Glyma.09g088500*) may delay flowering (Dietz *et al.* 2022). These supported our findings that the gene *VrPHYE* is the candidate for the flowering time in the mungbean. Several SNPs resulted in phyE amino acid changes between the wild mungbean ACC41 and the cultivated mungbeans (Fig. 5). All these cultivated mungbeans are early flowering. The amino acids changes occurred in the PAS and HKRD domains in the C-terminal (Fig. 5). These domains constitute the C-terminal output module (OPM) of the phytochromes (Cheng *et al.* 2021, Rockwell *et al.* 2006) which mediates dimerization and signal transmission to the downstream effectors (Cheng *et al.* 2021). So, the mutation(s) in the OPM of the phyE is likely the cause of early flowering in the mungbean. It is also worth noting that the *VrPHYE* showed different expression levels between the wild mungbean ACC41 and the cultivated mungbean KPS2 (Fig. 6). The different expression may stem from the sequence variations in the promotor and/or 5ʹ UTR regions between ACC41 and KPS2, especially the 38-bp InDel in the 5ʹ UTR region (Supplemental Fig. 1). Nonetheless, although fine mapping (Figs. 2, 3), gene sequencing (Figs. 4, 5), and gene expression analysis (Fig. 6) strongly suggested that *VrPHYE* is the candidate gene controlling flowering time in mungbean, additional study is necessary due to the fact that *VrPHYE* was not narrowed to a single gene and functional study of the *VrPHYE* was not conducted to confirm the gene has function in photoperiod regulation of flowering in the mungbean.

QTL mapping in azuki bean (*Vigna angularis* (Ohwi) Ohwi and Ohashi) using a population from a cross between cultivated and wild azuki beans demonstrated that phyE gene *LOC108331824* is one of the two candidate genes controlling flowering time at the QTL *qVaFld4.1*, the strongest-effect QTL for the flowering time (Li *et al.* 2017). Similarly, QTL mapping in cowpea (*Vigna unguiculata* (L.) Walp.) using a population of a cross between cultivated and wild cowpeas showed that the gene *Vigun09g050600* encoding phyE is a candidate gene controlling flowering time at the locus *CFt9*, the strongest-effect QTL for the flowering time (Lo *et al.* 2018). In our study, *VrPHYE* is the candidate gene flowering time in populations deriving from a cross between the cultivated and wild mungbeans. These suggest that phyE gene is the most important gene playing role in reducing sensitivity to photoperiod during the process of domestication of legume crops of the genus *Vigna*. It is worth noting that phyE proteins in mungbean, azuki bean, and cowpea were closely related (Fig. 7). Additional study should be conducted to determine how the phyE modulate the flowering time in the mungbean and other *Vigna* species.

In mungbean and azuki bean, flowering time and pod maturity time are highly correlated and the largest-effect QTLs controlling flowering time and maturity time are closely linked or mapped to the same position on the LG2 (Isemura *et al.* 2012, Kaga *et al.* 2008, Kajonphol *et al.* 2012, Li *et al.* 2017). The QTLs for these two traits on the LG2 are also closely linked or locate to the same position with QTL controlling seed weight (Isemura *et al.* 2012, Kaga *et al.* 2008, Li *et al.* 2017, Somta *et al.* 2015) and internode length (Isemura *et al.* 2012). It is possible the QTL on LG2 has a pleiotropic effect controlling flowering time, maturity time, seed weight and internode length in mungbean and azuki bean. Devlin *et al.* (1998) showed that *phyE* plays a predominant role in controlling the internode elongation response to end-of-day far-red light of *phyA phyB* mutants. Since the phyE gene is the candidate gene at the QTL on the LG2 responsible for flowering time in both mungbean (this study) and azuki bean (Li *et al.* 2017), the gene may also be responsible for maturity time and seed weight. If this is really the case, it would suggest that mutation(s) in phyE gene contributes greatly to phenology changes and seed weight during domestication of these legumes. Additional study is necessary to determine the effect of the phyE gene to the maturity and seed weight/seed yield.

## Author Contribution Statement

PS, KL and XC conceived idea, designed the study and secured research fund. PS and KL supervised the study. KA, KL and TY conducted field experiment. KA, KL, YL, JC and XY performed molecular experiments. KA carried out all data analyses. PS and KA wrote and revised the manuscript. All authors read and approved the manuscript.

## Supplementary Material

Supplemental Figure

Supplemental Tables

## Figures and Tables

**Table 1.** Location of effects of QTLs for days to first flowering detected in the F_2_ and F_2:3_ populations derived from a cross between mungbean cultivar Kamphaeng Saen 2 and wild mungbean accession ACC41

Population	QTL name	Position (cM)	Marker interval	LOD score	PVE (%)	Additive effect	Dominant effect
F_2_	*qFld2.1*	5.5	VrE1-SSR2–Vr05-ID019	24.34	17.79	–3.96	0.64
F_2:3_	*qFld2.1*	6.3	VrE1-SSR11–VrE1-SSR2	6.29	12.44	–4.09	1.49
