# Supplementary material for: Narrowing down a major QTL region reveals Phytochrome E (PHYE) as the candidate gene controlling flowering time in mungbean (Vigna radiata)
Source: Breed Sci. 2024 Feb 29;74(2):83–92. doi: 10.1270/jsbbs.23036 (PMC11442112; doi:10.1270/jsbbs.23036)
Supplement: Supplementary file 1 — Supplemental Figure [file 74_083_s1.pdf]

doi: 10.1270/jsbbs.23036

```
ACC41           CAAATCTAATACTAATTAACATCAAAGTATATCTTTAAGACATCTCTCAGCAATTTAAAT   60
KPS2            CAAATCTAATACTAATTAACATCAAAGTATATCTTTAAGACTTCTCTCATCAATTTAAAT   60
Sulv1           CAAATCTAATACTAATTAACATCAAAGTATATCTTTAAGACTTCTCTCATCAATTTAAAT   60
Jilv7           CAAATCTAATACTAATTAACATCAAAGTATATCTTTAAGACTTCTCTCATCAATTTAAAT   60
VC1973A         CAAATCTAATACTAATTAACATCAAAGTATATCTTTAAGACTTCTCTCATCAATTTAAAT   60
                ***************************************** ******* **********

ACC41           TTATTTATGAAATTTAAAAGTATTTATTCCACTTAATTAAAATACATTTTCCATCAATTA   120
KPS2            TTATTTATGAAATTTAAAAGTATTTATTCCACTTAATTAAAATACATTTTCCATCAATTA   120
Sulv1           TTATTTATGAAATTTAAAAGTATTTATTCCACTTAATTAAAATACATTTTCCATCAATTA   120
Jilv7           TTATTTATGAAATTTAAAAGTATTTATTCCACTTAATTAAAATACATTTTCCATCAATTA   120
VC1973A         TTATTTATGAAATTTAAAAGTATTTATTCCACTTAATTAAAATACATTTTCCATCAATTA   120
                ************************************************************

ACC41           ATATTTCAAATTTAAAATTAAAATTATTTTTCATATAATTAAATGTTAATATTTTATCAT   180
KPS2            ATATTTCATATTTAAAATTAAAATTATTTTTCATATAATTAAATGTTAATATTTTATCAT   180
Sulv1           ATATTTCATATTTAAAATTAAAATTATTTTTCATATAATTAAATGTTAATATTTTATCAT   180
Jilv7           ATATTTCATATTTAAAATTAAAATTATTTTTCATATAATTAAATGTTAATATTTTATCAT   180
VC1973A         ATATTTCATATTTAAAATTAAAATTATTTTTCATATAATTAAATGTTAATATTTTATCAT   180
                ******** ***************************************************

ACC41           TTATTTTATTTTAAAGATAAGTAAAAATCAAACTCTAAATTTAACTCCCCTGTAATCATA   240
KPS2            TTATTTTATTTTAAAGATAAGTAAAAATCGAACTCTAAATTTAACTCCCCTGTAATCCAA   240
Sulv1           TTATTTTATTTTAAAGATAAGTAAAAATCGAACTCTAAATTTAACTCCCCTGTAATCCAA   240
Jilv7           TTATTTTATTTTAAAGATAAGTAAAAATCGAACTCTAAATTTAACTCCCCTGTAATCCAA   240
VC1973A         TTATTTTATTTTAAAGATAAGTAAAAATCGAACTCTAAATTTAACTCCCCTGTAATCCAA   240
                ***************************** ***************************  *

ACC41           TTCTCATTGATTTTTCAGAACCTATCAAACGAAACAAATACCAGAAACCGAATTTCTGGG   300
KPS2            TTCTCATTGATTTTTCAGAACCTATCAAACA-AACATATACCAGAAACCGAATTTCTGGG   299
Sulv1           TTCTCATTGATTTTTCAGAACCTATCAAACA-AACATATACCAGAAACCGAATTTCTGGG   299
Jilv7           TTCTCATTGATTTTTCAGAACCTATCAAACA-AACATATACCAGAAACCGAATTTCTGGG   299
VC1973A         TTCTCATTGATTTTTCAGAACCTATCAAACA-AACATATACCAGAAACCGAATTTCTGGG   299
                ******************************  **** ***********************

ACC41           CACTGAATTCCAACTTAAAAACCTGTACTATTTCAATGAATATAGAAATAAAAGTTGGAA   360
KPS2            CACTGAATTCCAACTTAAAAACCTGTACTATTTCAATAAATATAGAAATAAAAGTTGGAA   359
Sulv1           CACTGAATTCCAACTTAAAAACCTGTACTATTTCAATAAATATAGAAATAAAAGTTGGAA   359
Jilv7           CACTGAATTCCAACTTAAAAACCTGTACTATTTCAATAAATATAGAAATAAAAGTTGGAA   359
VC1973A         CACTGAATTCCAACTTAAAAACCTGTACTATTTCAATAAATATAGAAATAAAAGTTGGAA   359
                ************************************* **********************

ACC41           TAATTTTCTCTGGGACAATGTTGTTTGGGAAAAAGAAAACCTAGAAGATAACCTTAGTTT   420
KPS2            TAATTTTCTCTGGGACAATGTTGTTTGGGAAAAAGAAAACCTAGAAGATAACCTTAGTTT   419
Sulv1           TAATTTTCTCTGGGACAATGTTGTTTGGGAAAAAGAAAACCTAGAAGATAACCTTAGTTT   419
Jilv7           TAATTTTCTCTGGGACAATGTTGTTTGGGAAAAAGAAAACCTAGAAGATAACCTTAGTTT   419
VC1973A         TAATTTTCTCTGGGACAATGTTGTTTGGGAAAAAGAAAACCTAGAAGATAACCTTAGTTT   419
                ************************************************************

ACC41           AACCAATTGCATGAAAAGAACATGTTGGGTGGAATAGCCTATAAATTGATAAGTCCAAAG   480
KPS2            AACCAATTGCATGAAAAGACCATGTTGGGTGGAATAGCCTATAAATTGATAAGTCCAAAG   479
Sulv1           AACCAATTGCATGAAAAGACCATGTTGGGTGGAATAGCCTATAAATTGATAAGTCCAAAG   479
Jilv7           AACCAATTGCATGAAAAGACCATGTTGGGTGGAATAGCCTATAAATTGATAAGTCCAAAG   479
VC1973A         AACCAATTGCATGAAAAGACCATGTTGGGTGGAATAGCCTATAAATTGATAAGTCCAAAG   479
                ******************* ****************************************

ACC41           TAGATTCATGGATTCTAAACAAGCAGCAAAAAAGAAGTGGATGCAAAAACCTCTATTCTC   540
KPS2            TAGATTCATGGATTCTAAACAAGCAGCAAAAAAGAAGTGGATGCAAAAACCTCAATTCTC   539
Sulv1           TAGATTCATGGATTCTAAACAAGCAGCAAAAAAGAAGTGGATGCAAAAACCTCAATTCTC   539
Jilv7           TAGATTCATGGATTCTAAACAAGCAGCAAAAAAGAAGTGGATGCAAAAACCTCAATTCTC   539
VC1973A         TAGATTCATGGATTCTAAACAAGCAGCAAAAAAGAAGTGGATGCAAAAACCTCAATTCTC   539
                ***************************************************** ******

ACC41           AACCACTTAAATAAATCCGTCAAGTCAATTCAACTTTTGATTTCTCATTGTGTGAAGAAC   600
KPS2            AACCACTTAAATAAATCCGTCAAGTCAATTCAACTTTTGATTTCTCATTGTGTGAAGAAC   599
Sulv1           AACCACTTAAATAAATCCGTCAAGTCAATTCAACTTTTGATTTCTCATTGTGTGAAGAAC   599
Jilv7           AACCACTTAAATAAATCCGTCAAGTCAATTCAACTTTTGATTTCTCATTGTGTGAAGAAC   599
VC1973A         AACCACTTAAATAAATCCGTCAAGTCAATTCAACTTTTGATTTCTCATTGTGTGAAGAAC   599
                ************************************************************

ACC41           ATTTCAAATACTCATCAAGATAATCAGAAAAAATATATTCATTAAAAGTTGTTGTGAAAA   660
KPS2            ATTTCAAATACTCATCAAGATAATCAGAAAAAATATATTCATTAAAAGTTGTTGTGAAAA   659
Sulv1           ATTTCAAATACTCATCAAGATAATCAGAAAAAATATATTCATTAAAAGTTGTTGTGAAAA   659
Jilv7           ATTTCAAATACTCATCAAGATAATCAGAAAAAATATATTCATTAAAAGTTGTTGTGAAAA   659
```

```
VC1973A         ATTTCAAATACTCATCAAGATAATCAGAAAAAATATATTCATTAAAAGTTGTTGTGAAAA    659
                ************************************************************

ACC41           AGTTTTCTTATTTGTTTTAGAAAAGAATTAAGTAAAAAGCTCATAATAACACAAATAAAA    720
KPS2            AGTTTTCATATTTGTTTTAGAAAAGAATTAAGTAAAAAGCTCATAATAACACAAATAAAA    719
Sulv1           AGTTTTCATATTTGTTTTAGAAAAGAATTAAGTAAAAAGCTCATAATAACACAAATAAAA    719
Jilv7           AGTTTTCATATTTGTTTTAGAAAAGAATTAAGTAAAAAGCTCATAATAACACAAATAAAA    719
VC1973A         AGTTTTCATATTTGTTTTAGAAAAGAATTAAGTAAAAAGCTCATAATAACACAAATAAAA    719
                ******* ****************************************************

ACC41           TTGTATCTATTTGGGTGTTTTCCATCTCCAACCATGAATAATAAAACTTCTTTTCACAGT    780
KPS2            TTGTATCTATTTGGGTATTTTCCATCTCCAACCATGAATAATAAAACTTCTTTTCACAGT    779
Sulv1           TTGTATCTATTTGGGTATTTTCCATCTCCAACCATGAATAATAAAACTTCTTTTCACAGT    779
Jilv7           TTGTATCTATTTGGGTATTTTCCATCTCCAACCATGAATAATAAAACTTCTTTTCACAGT    779
VC1973A         TTGTATCTATTTGGGTATTTTCCATCTCCAACCATGAATAATAAAACTTCTTTTCACAGT    779
                **************** *******************************************

ACC41           GAAAACATGTGTGGTCATATTTCGCAAACCATGAAACAAAGACAGAAGGACCGCATTGGC    840
KPS2            GAAAACATGTGTGGTCATATTTCGCAAACCATGAAACAAAGACAGAAGGACCGCATTGGC    839
Sulv1           GAAAACATGTGTGGTCATATTTCGCAAACCATGAAACAAAGACAGAAGGACCGCATTGGC    839
Jilv7           GAAAACATGTGTGGTCATATTTCGCAAACCATGAAACAAAGACAGAAGGACCGCATTGGC    839
VC1973A         GAAAACATGTGTGGTCATATTTCGCAAACCATGAAACAAAGACAGAAGGACCGCATTGGC    839
                ************************************************************

ACC41           ATTTCTTATCTCAGTTTTTGTGGTTCACCTTCGTCTTCTGTCCATTCAGTTTTCACTTCA    900
KPS2            ATTTCTTATCTCAGTTTTTGTGGTTCACCTTCGTCTTCTGTCCATTCAGTTTTCACTTCA    899
Sulv1           ATTTCTTATCTCAGTTTTTGTGGTTCACCTTCGTCTTCTGTCCATTCAGTTTTCACTTCA    899
Jilv7           ATTTCTTATCTCAGTTTTTGTGGTTCACCTTCGTCTTCTGTCCATTCAGTTTTCACTTCA    899
VC1973A         ATTTCTTATCTCAGTTTTTGTGGTTCACCTTCGTCTTCTGTCCATTCAGTTTTCACTTCA    899
                ************************************************************

ACC41           TCGTTTCATTGTCCTTCTAATCACCGACACAGATTTATTTACACAACCTCAAACCAATGA    960
KPS2            TCGTTTCATTGTCCTTCTAATCACCGACACAGATTTATTTACACAACCTCAAACCAATGA    959
Sulv1           TCGTTTCATTGTCCTTCTAATCACCGACACAGATTTATTTACACAACCTCAAACCAATGA    959
Jilv7           TCGTTTCATTGTCCTTCTAATCACCGACACAGATTTATTTACACAACCTCAAACCAATGA    959
VC1973A         TCGTTTCATTGTCCTTCTAATCACCGACACAGATTTATTTACACAACCTCAAACCAATGA    959
                ************************************************************

ACC41           TGAAGTTTAGTGTTTCATAGTATTT-TTTAACTCAATCACATTGATTGAAGCAAAATATG    1019
KPS2            TGAAGTTTAGTGTTTCATAGTATTTTTTTAACTCAATCACATTGATTGAAGCAAAATATG    1019
Sulv1           TGAAGTTTAGTGTTTCATAGTATTTTTTTAACTCAATCACATTGATTGAAGCAAAATATG    1019
Jilv7           TGAAGTTTAGTGTTTCATAGTATTTTTTTAACTCAATCACATTGATTGAAGCAAAATATG    1019
VC1973A         TGAAGTTTAGTGTTTCATAGTATTTTTTTAACTCAATCACATTGATTGAAGCAAAATATG    1019
                ************************* **********************************

ACC41           ATAATATAATTTAATCCATTCATGGGGAATCTCAGTCATTCTGTGAGGAAGTTCATAAAG    1079
KPS2            ATAATATAATTTAATCCATTCATGGGGCATCTCAGTCATTCTGTGAGGAAGTTCATAAAG    1079
Sulv1           ATAATATAATTTAATCCATTCATGGGGCATCTCAGTCATTCTGTGAGGAAGTTCATAAAG    1079
Jilv7           ATAATATAATTTAATCCATTCATGGGGCATCTCAGTCATTCTGTGAGGAAGTTCATAAAG    1079
VC1973A         ATAATATAATTTAATCCATTCATGGGGCATCTCAGTCATTCTGTGAGGAAGTTCATAAAG    1079
                *************************** ********************************

ACC41           ATAAAGTTATTAATCTCTTCAAGTAAATACAGAAGTTTAGAATATCATTGTGGTTTATAA    1139
KPS2            ATAAAGTTATTAATCTCTTCAAGTAAATACAGAAGTTTAGAATATCATTGTGGTTTATAA    1139
Sulv1           ATAAAGTTATTAATCTCTTCAAGTAAATACAGAAGTTTAGAATATCATTGTGGTTTATAA    1139
Jilv7           ATAAAGTTATTAATCTCTTCAAGTAAATACAGAAGTTTAGAATATCATTGTGGTTTATAA    1139
VC1973A         ATAAAGTTATTAATCTCTTCAAGTAAATACAGAAGTTTAGAATATCATTGTGGTTTATAA    1139
                ************************************************************

ACC41           TCTCTATTATGAAGGACAAATGCTGTAGTTGGAATAAAGGAGAAAATAACATTAAAAGCT    1199
KPS2            TCTCTATTATGAAGGACAAATGCTGTAGTTGGAATAAAGGAGAAAATAACATTAAAAGCT    1199
Sulv1           TCTCTATTATGAAGGACAAATGCTGTAGTTGGAATAAAGGAGAAAATAACATTAAAAGCT    1199
Jilv7           TCTCTATTATGAAGGACAAATGCTGTAGTTGGAATAAAGGAGAAAATAACATTAAAAGCT    1199
VC1973A         TCTCTATTATGAAGGACAAATGCTGTAGTTGGAATAAAGGAGAAAATAACATTAAAAGCT    1199
                ************************************************************

ACC41           GTTAAATTTTAAGTAATGGTGATTACAAGCTGAAAATTGTTTCTAGAATTTGAATTTGTG    1259
KPS2            GTTAAATTTTAAGTAATGGTGATTACAAGCTGAAAATTGTTTCTAGAATTTGAATTTGTG    1259
Sulv1           GTTAAATTTTAAGTAATGGTGATTACAAGCTGAAAATTGTTTCTAGAATTTGAATTTGTG    1259
Jilv7           GTTAAATTTTAAGTAATGGTGATTACAAGCTGAAAATTGTTTCTAGAATTTGAATTTGTG    1259
VC1973A         GTTAAATTTTAAGTAATGGTGATTACAAGCTGAAAATTGTTTCTAGAATTTGAATTTGTG    1259
                ************************************************************

ACC41           AATGGAGGAGATGCAATGTTATGCAGTGACTAAGCATTAAAAGAAAGATTTTGCTCCAAA    1319
```

```
KPS2            AATGGAGGAGATGCAATGTTATGCAGTGACTAAGCATTAAAAGAAAGATTTTGCTCCAAA   1319
Sulv1           AATGGAGGAGATGCAATGTTATGCAGTGACTAAGCATTAAAAGAAAGATTTTGCTCCAAA   1319
Jilv7           AATGGAGGAGATGCAATGTTATGCAGTGACTAAGCATTAAAAGAAAGATTTTGCTCCAAA   1319
VC1973A         AATGGAGGAGATGCAATGTTATGCAGTGACTAAGCATTAAAAGAAAGATTTTGCTCCAAA   1319
                ************************************************************

ACC41           AGAATATGAGTCCGAGTCTGCCGCCTACTCCTTCCTCTGTGGTTTTCTTGGATTGGTGGA   1379
KPS2            AGAATATGAGTCCGAGTCTGCCGCCTACTCCTTCCTCTGTGGTTTTCTTGGATTGGTGGA   1379
Sulv1           AGAATATGAGTCCGAGTCTGCCGCCTACTCCTTCCTCTGTGGTTTTCTTGGATTGGTGGA   1379
Jilv7           AGAATATGAGTCCGAGTCTGCCGCCTACTCCTTCCTCTGTGGTTTTCTTGGATTGGTGGA   1379
VC1973A         AGAATATGAGTCCGAGTCTGCCGCCTACTCCTTCCTCTGTGGTTTTCTTGGATTGGTGGA   1379
                ************************************************************

ACC41           AAAAGATGGAAAAGTAACACTGAAGTGGTGAAGTGAGAAGATGAAATTATA--GGATCTT   1437
KPS2            AAAAGATGGAAAAGTAACACTGAAGTGGTGAAGTGAGAAGATGAAATTATATGAGATCTT   1439
Sulv1           AAAAGATGGAAAAGTAACACTGAAGTGGTGAAGTGAGAAGATGAAATTATA--GGATCTT   1437
Jilv7           AAAAGATGGAAAAGTAACACTGAAGTGGTGAAGTGAGAAGATGAAATTATA--GGATCTT   1437
VC1973A         AAAAGATGGAAAAGTAACACTGAAGTGGTGAAGTGAGAAGATGAAATTATA--GGATCTT   1437
                ***************************************************   ******

ACC41           GTCTTTTTGACAAATAAACTAAGTGAAGATTTCCAAGTTAACCAATTTGTGAATGTTTTC   1497
KPS2            GTCTTTTTGACAAATAAACTAAGTGAAGATTTCCAAGTTAACCAATTTGTGAATGTTTTC   1499
Sulv1           GTCTTTTTGACAAATAAACTAAGTGAAGATTTCCAAGTTAACCAATTTGTGAATGTTTTC   1497
Jilv7           GTCTTTTTGACAAATAAACTAAGTGAAGATTTCCAAGTTAACCAATTTGTGAATGTTTTC   1497
VC1973A         GTCTTTTTGACAAATAAACTAAGTGAAGATTTCCAAGTTAACCAATTTGTGAATGTTTTC   1497
                ************************************************************

ACC41           CTAGAATTACTGTCGAAACTGGCTGGCTAA------------------------------   1527
KPS2            CTAGAATTACTGTCGAAACTGGCTGGCTAAGGAATTTGTGCTTAGTTATTATGTCTTTGG   1559
Sulv1           CTAGAATTACTGTCGAAACTGGCTGGCTAAGGAATTTGTGCTTAGTTATTATGTCTTTGG   1557
Jilv7           CTAGAATTACTGTCGAAACTGGCTGGCTAAGGAATTTGTGCTTAGTTATTATGTCTTTGG   1557
VC1973A         CTAGAATTACTGTCGAAACTGGCTGGCTAAGGAATTTGTGCTTAGTTATTATGTCTTTGG   1557
                ******************************

ACC41           --------GTAAGAGAGGGTAGAAATAAGCTTGAGTTGGACTGAACGAAAACAACATAGG   1579
KPS2            CAGTAAAAGTAAGAGAGGGTAGAAATAAGCTTGAGTTGGACTGAACGAAAACAACATAGG   1619
Sulv1           CAGTAAAAGTAAGAGAGGGTAGAAATAAGCTTGAGTTGGACTGAACGAAAACAACATAGG   1617
Jilv7           CAGTAAAAGTAAGAGAGGGTAGAAATAAGCTTGAGTTGGACTGAACGAAAACAACATAGG   1617
VC1973A         CAGTAAAAGTAAGAGAGGGTAGAAATAAGCTTGAGTTGGACTGAACGAAAACAACATAGG   1617
                        ****************************************************

ACC41           AGAGACATTAAGTGGGAGGTGCACATCATAGGGCAATTCCTTGTCGGTTTAACACAACAG   1639
KPS2            AGAGACATTAAGTGGGAGGTGCACATCATAGGGCAATTCCTTGTCGGTTTAACACAACAG   1679
Sulv1           AGAGACATTAAGTGGGAGGTGCACATCATAGGGCAATTCCTTGTCGGTTTAACACAACAG   1677
Jilv7           AGAGACATTAAGTGGGAGGTGCACATCATAGGGCAATTCCTTGTCGGTTTAACACAACAG   1677
VC1973A         AGAGACATTAAGTGGGAGGTGCACATCATAGGGCAATTCCTTGTCGGTTTAACACAACAG   1677
                ************************************************************

ACC41           AAGAACAATGCACTTTGAATAAAAACTATCTTCTCAATTTTTCGAGTTGAGGGTATCTAA   1699
KPS2            AAGAACAATGCACTTTGAATAAAAACTATCTTCTCAATTTTTCGAGTTGAGGGTATCTAA   1739
Sulv1           AAGAACAATGCACTTTGAATAAAAACTATCTTCTCAATTTTTCGAGTTGAGGGTATCTAA   1737
Jilv7           AAGAACAATGCACTTTGAATAAAAACTATCTTCTCAATTTTTCGAGTTGAGGGTATCTAA   1737
VC1973A         AAGAACAATGCACTTTGAATAAAAACTATCTTCTCAATTTTTCGAGTTGAGGGTATCTAA   1737
                ************************************************************

ACC41           TATGTTTGTGTATATATCTCCTTCACAAACCACACAGTAAAAAAAGCTGGAATCAA-ACT   1758
KPS2            TATGTTTGTGTATATATCTCCTTCACAAACCACACAGTAAAAAAGCTGGAATCAAACTGG   1799
Sulv1           TATGTTTGTGTATATATCTCCTTCACAAACCACACAGTAAAAAAGCTGGAATCAAACTGG   1797
Jilv7           TATGTTTGTGTATATATCTCCTTCACAAACCACACAGTAAAAAAGCTGGAATCAAACTGG   1797
VC1973A         TATGTTTGTGTATATATCTCCTTCACAAACCACACAGTAAAAAAGCTGGAATCAAACTGG   1797
                ********************************************    * *   **

ACC41           GGTTTCATAGAAGGTGCAATACAAGGTGAGCAAACACATTTGGCTTTTCGTCAAACAGCC   1818
KPS2            GTTTTCATAGAAGGTGCAATACAAGGTGAGCAAACACATTTGGCTTTTTGTCAAACAGCC   1859
Sulv1           --TTTCATAGAAGGTGCAATACAAGGTGAGCAAACACATTTGGCTTTTTGTCAAACAGCC   1855
Jilv7           --TTTCATAGAAGGTGCAATACAAGGTGAGCAAACACATTTGGCTTTTTGTCAAACAGCC   1855
VC1973A         --TTTCATAGAAGGTGCAATACAAGGTGAGCAAACACATTTGGCTTTTTGTCAAACAGCC   1855
                  ********************************************** ***********

ACC41           TTGAAAATGAGCTATGGAAGCAGAGGGAAGCTGAAAGATACAAGCTTGAGCACATCAGCT   1878
KPS2            TTGAAAATGAGCTATGGAAGCAGAGGGAAGCTGAAAGATACAAGCTTGAGCACATCAGCT   1919
Sulv1           TTGAAAATGAGCTATGGAAGCAGAGGGAAGCTGAAAGATACAAGCTTGAGCACATCAGCT   1915
Jilv7           TTGAAAATGAGCTATGGAAGCAGAGGGAAGCTGAAAGATACAAGCTTGAGCACATCAGCT   1915
VC1973A         TTGAAAATGAGCTATGGAAGCAGAGGGAAGCTGAAAGATACAAGCTTGAGCACATCAGCT   1915
```

```
                    ************************************************************

ACC41               GAAAGCAACATGAACAGCAGGAGGGACAAGATACTGGCTCAGTACAGTGCAGATGCTGAG    1938
KPS2                GAAAGCAACATGAACAGCAGGAGGGACAAGATACTGGCTCAGTACAGTGCAGATGCTGAG    1979
Sulv1               GAAAGCAACATGAACAGCAGGAGGGACAAGATACTGGCTCAGTACAGTGCAGATGCTGAG    1975
Jilv7               GAAAGCAACATGAACAGCAGGAGGGACAAGATACTGGCTCAGTACAGTGCAGATGCTGAG    1975
VC1973A             GAAAGCAACATGAACAGCAGGAGGGACAAGATACTGGCTCAGTACAGTGCAGATGCTGAG    1975
                    ************************************************************

ACC41               ATTCTGGCTGAGTTTGAGCAGTCTGGTGTGTCTGGCAAATCATTTGACTACTCCAGGATG    1998
KPS2                ATTCTGGCTGAGTTTGAGCAGTCTGGTGTGTCTGGAAAATCATTTGACTACTCCAGGATG    2039
Sulv1               ATTCTGGCTGAGTTTGAGCAGTCTGGTGTGTCTGGAAAATCATTTGACTACTCCAGGATG    2035
Jilv7               ATTCTGGCTGAGTTTGAGCAGTCTGGTGTGTCTGGAAAATCATTTGACTACTCCAGGATG    2035
VC1973A             ATTCTGGCTGAGTTTGAGCAGTCTGGTGTGTCTGGAAAATCATTTGACTACTCCAGGATG    2035
                    *********************************** ************************

ACC41               GTCCTTGATCCTCCCAGGTTAGTGTCTGGAGAAAAAATGACTGCATATCTGTCCAAAATC    2058
KPS2                GTCCTTGATCCTCCCAGGTTAGTGTCTGGAGAAAAAATGACTGCATATCTGTCCAAAATC    2099
Sulv1               GTCCTTGATCCTCCCAGGTTAGTGTCTGGAGAAAAAATGACTGCATATCTGTCCAAAATC    2095
Jilv7               GTCCTTGATCCTCCCAGGTTAGTGTCTGGAGAAAAAATGACTGCATATCTGTCCAAAATC    2095
VC1973A             GTCCTTGATCCTCCCAGGTTAGTGTCTGGAGAAAAAATGACTGCATATCTGTCCAAAATC    2095
                    ************************************************************

ACC41               CAAAGGGGTGGCCTTATCCAACCCTTTGGTTGTATGCTTGCAATTGAGGAACCCACTTTT    2118
KPS2                CAAAGGGGTGGCCTTATCCAACCCTTTGGTTGTATGCTTGCAATTGAGGAACCCACTTTT    2159
Sulv1               CAAAGGGGTGGCCTTATCCAACCCTTTGGTTGTATGCTTGCAATTGAGGAACCCACTTTT    2155
Jilv7               CAAAGGGGTGGCCTTATCCAACCCTTTGGTTGTATGCTTGCAATTGAGGAACCCACTTTT    2155
VC1973A             CAAAGGGGTGGCCTTATCCAACCCTTTGGTTGTATGCTTGCAATTGAGGAACCCACTTTT    2155
                    ************************************************************

ACC41               AGGATCATTGGGTACAGTGAGAATTGCTTTCAGTTGTTGGGTTTGGAGCGGCAAATTGAC    2178
KPS2                AGGATCATTGGGTACAGTGAGAATTGCTTTCAGTTGTTGGGTTTGGAGCGGCAAATTGAC    2219
Sulv1               AGGATCATTGGGTACAGTGAGAATTGCTTTCAGTTGTTGGGTTTGGAGCGGCAAATTGAC    2215
Jilv7               AGGATCATTGGGTACAGTGAGAATTGCTTTCAGTTGTTGGGTTTGGAGCGGCAAATTGAC    2215
VC1973A             AGGATCATTGGGTACAGTGAGAATTGCTTTCAGTTGTTGGGTTTGGAGCGGCAAATTGAC    2215
                    ************************************************************

ACC41               TCTAACATGTTAATAAATTTGATTGGGGTTGATGCAACGACCCCTTTTCACTCCACCGTCT    2238
KPS2                TCTAACATGTTAATAAATTTGATTGGGGTTGATGCAACGACCCCTTTTCACTCCACCTTCT    2279
Sulv1               TCTAACATGTTAATAAATTTGATTGGGGTTGATGCAACGACCCCTTTTCACTCCACCTTCT    2275
Jilv7               TCTAACATGTTAATAAATTTGATTGGGGTTGATGCAACGACCCCTTTTCACTCCACCTTCT    2275
VC1973A             TCTAACATGTTAATAAATTTGATTGGGGTTGATGCAACGACCCCTTTTCACTCCACCTTCT    2275
                    ******************************************************** ***

ACC41               GGGGCTTCCCTGGCTAAAGCTGTGGCTTCCAGGGAAATTTCTCTTCTGAACCCGATTTGG    2298
KPS2                GGGGCTTCCCTAGCTAAAGCTGTGGCTTCCAGGGAAATTTCTCTTCTGAACCCGATTTGG    2339
Sulv1               GGGGCTTCCCTAGCTAAAGCTGTGGCTTCCAGGGAAATTTCTCTTCTGAACCCGATTTGG    2335
Jilv7               GGGGCTTCCCTAGCTAAAGCTGTGGCTTCCAGGGAAATTTCTCTTCTGAACCCGATTTGG    2335
VC1973A             GGGGCTTCCCTAGCTAAAGCTGTGGCTTCCAGGGAAATTTCTCTTCTGAACCCGATTTGG    2335
                    *********** ************************************************

ACC41               GTCTATGCAAGGACAACCCAGAAGCCATTTTATGCCATACTACATAGGATCGATGTTGGG    2358
KPS2                GTCTATGCAAGGACAACCCAGAAGCCATTTTATGCCATACTACATAGGATCGATGTTGGG    2399
Sulv1               GTCTATGCAAGGACAACCCAGAAGCCATTTTATGCCATACTACATAGGATCGATGTTGGG    2395
Jilv7               GTCTATGCAAGGACAACCCAGAAGCCATTTTATGCCATACTACATAGGATCGATGTTGGG    2395
VC1973A             GTCTATGCAAGGACAACCCAGAAGCCATTTTATGCCATACTACATAGGATCGATGTTGGG    2395
                    ************************************************************

ACC41               GTTGTGATTGATTTGGAGCCAGCGCGGATGAGTGATCCGGCATTGTCACTTGCCGGGGCA    2418
KPS2                GTTGTGATTGATTTGGAGCCAGCGCGGATGAGTGATCCGGCATTGTCACTTGCCGGGGCA    2459
Sulv1               GTTGTGATTGATTTGGAGCCAGCGCGGATGAGTGATCCGGCATTGTCACTTGCCGGGGCA    2455
Jilv7               GTTGTGATTGATTTGGAGCCAGCGCGGATGAGTGATCCGGCATTGTCACTTGCCGGGGCA    2455
VC1973A             GTTGTGATTGATTTGGAGCCAGCGCGGATGAGTGATCCGGCATTGTCACTTGCCGGGGCA    2455
                    ************************************************************

ACC41               GTTCAATCCCAGAAGCTGGCTGTGAGGGCTATTTCAAGGCTGCAATCTCTTCCTGGGGAA    2478
KPS2                GTTCAATCCCAGAAGCTGGCTGTGAGGGCTATTTCAAGGCTGCAATCTCTTCCTGGGGAA    2519
Sulv1               GTTCAATCCCAGAAGCTGGCTGTGAGGGCTATTTCAAGGCTGCAATCTCTTCCTGGGGAA    2515
Jilv7               GTTCAATCCCAGAAGCTGGCTGTGAGGGCTATTTCAAGGCTGCAATCTCTTCCTGGGGAA    2515
VC1973A             GTTCAATCCCAGAAGCTGGCTGTGAGGGCTATTTCAAGGCTGCAATCTCTTCCTGGGGAA    2515
                    ************************************************************

ACC41               GATATTGGCTTGCTGTGTGACACAGTTGTTGAGGAAGTGCAGAAGCTTACCGGATATGAC    2538
KPS2                GATATTGGCTTGCTGTGTGACACAGTTGTTGAGGAAGTGCAGAAGCTTACCGGATATGAC    2579
```

```
Sulv1       GATATTGGCTTGCTGTGTGACACAGTTGTTGAGGAAGTGCAGAAGCTTACCGGATATGAC   2575
Jilv7       GATATTGGCTTGCTGTGTGACACAGTTGTTGAGGAAGTGCAGAAGCTTACCGGATATGAC   2575
VC1973A     GATATTGGCTTGCTGTGTGACACAGTTGTTGAGGAAGTGCAGAAGCTTACCGGATATGAC   2575
            ************************************************************

ACC41       AGGGTAATGGTTTATAAGTTTCATGAGGATGATCATGGTGAGGTTGTAGCTGAAATTAGG   2598
KPS2        AGGGTAATGGTTTATAAGTTTCATGAGGATGATCATGGTGAGGTTGTAGCTGAGATTAGG   2639
Sulv1       AGGGTAATGGTTTATAAGTTTCATGAGGATGATCATGGTGAGGTTGTAGCTGAGATTAGG   2635
Jilv7       AGGGTAATGGTTTATAAGTTTCATGAGGATGATCATGGTGAGGTTGTAGCTGAGATTAGG   2635
VC1973A     AGGGTAATGGTTTATAAGTTTCATGAGGATGATCATGGTGAGGTTGTAGCTGAGATTAGG   2635
            ***************************************************** ******

ACC41       AGGTCAGATTTGGAGCCTTACCTGGGTTTACATTATCCTGCAACAGATATCCCTCAAGCT   2658
KPS2        AGGTCAGATTTGGAGCCTTACCTGGGTTTACATTATCCTGCAACAGATATCCCTCAAGCT   2699
Sulv1       AGGTCAGATTTGGAGCCTTACCTGGGTTTACATTATCCTGCAACAGATATCCCTCAAGCT   2695
Jilv7       AGGTCAGATTTGGAGCCTTACCTGGGTTTACATTATCCTGCAACAGATATCCCTCAAGCT   2695
VC1973A     AGGTCAGATTTGGAGCCTTACCTGGGTTTACATTATCCTGCAACAGATATCCCTCAAGCT   2695
            ************************************************************

ACC41       TCTCGCTTCTTGTTCAAGCAAAACCGGGTTAGGATTATTTGTGATTGCCATGCAAAGCCA   2718
KPS2        TCTCGCTTCTTGTTCAAGCAAAACCGGGTTAGGATTATTTGTGATTGCCATGCAAAGCCA   2759
Sulv1       TCTCGCTTCTTGTTCAAGCAAAACCGGGTTAGGATTATTTGTGATTGCCATGCAAAGCCA   2755
Jilv7       TCTCGCTTCTTGTTCAAGCAAAACCGGGTTAGGATTATTTGTGATTGCCATGCAAAGCCA   2755
VC1973A     TCTCGCTTCTTGTTCAAGCAAAACCGGGTTAGGATTATTTGTGATTGCCATGCAAAGCCA   2755
            ************************************************************

ACC41       GTTAAGGTCATTCAGAGTGAAGAATTAAGGCAACCTCTTTGCTTGGTGAATTCAACACTT   2778
KPS2        GTTAAGGTCATTCAGAGTGAAGAATTAAGGCAACCTCTTTGCTTGGTGAATTCAACACTT   2819
Sulv1       GTTAAGGTCATTCAGAGTGAAGAATTAAGGCAACCTCTTTGCTTGGTGAATTCAACACTT   2815
Jilv7       GTTAAGGTCATTCAGAGTGAAGAATTAAGGCAACCTCTTTGCTTGGTGAATTCAACACTT   2815
VC1973A     GTTAAGGTCATTCAGAGTGAAGAATTAAGGCAACCTCTTTGCTTGGTGAATTCAACACTT   2815
            ************************************************************

ACC41       AGGTTACCACATGGTTGTCACACACAGTACATGGCCAACATGGGATCAATTGCCTCTCTG   2838
KPS2        AGGTTACCACATGGTTGTCACACACAGTACATGGCCAACATGGGATCAATTGCCTCTCTG   2879
Sulv1       AGGTTACCACATGGTTGTCACACACAGTACATGGCCAACATGGGATCAATTGCCTCTCTG   2875
Jilv7       AGGTTACCACATGGTTGTCACACACAGTACATGGCCAACATGGGATCAATTGCCTCTCTG   2875
VC1973A     AGGTTACCACATGGTTGTCACACACAGTACATGGCCAACATGGGATCAATTGCCTCTCTG   2875
            ************************************************************

ACC41       GTGATGGCAATTATAGTCAATGGAAAGAATGAAACAAGGCTTTGGGGTTTGCTAGTTTGT   2898
KPS2        GTGATGGCAATTATAGTCAATGGAAAGAATGAAACAAGGCTTTGGGGTTTGCTAGTTTGT   2939
Sulv1       GTGATGGCAATTATAGTCAATGGAAAGAATGAAACAAGGCTTTGGGGTTTGCTAGTTTGT   2935
Jilv7       GTGATGGCAATTATAGTCAATGGAAAGAATGAAACAAGGCTTTGGGGTTTGCTAGTTTGT   2935
VC1973A     GTGATGGCAATTATAGTCAATGGAAAGAATGAAACAAGGCTTTGGGGTTTGCTAGTTTGT   2935
            ************************************************************

ACC41       CATCACACTTCACCACGATTTGTTTCTTTCCCGGTTCGCTATGCTTGTGAGTTCCTAATG   2958
KPS2        CATCACACTTCACCACGATTTGTTTCTTTCCCGGTTCGCTATGCTTGTGAGTTCCTAATG   2999
Sulv1       CATCACACTTCACCACGATTTGTTTCTTTCCCGGTTCGCTATGCTTGTGAGTTCCTAATG   2995
Jilv7       CATCACACTTCACCACGATTTGTTTCTTTCCCGGTTCGCTATGCTTGTGAGTTCCTAATG   2995
VC1973A     CATCACACTTCACCACGATTTGTTTCTTTCCCGGTTCGCTATGCTTGTGAGTTCCTAATG   2995
            ************************************************************

ACC41       CAGGCTTTTGGACTGCAACTTTATATGGAGATTCAATTGGCATCACAAATGGCAGAAAAG   3018
KPS2        CAGGCTTTTGGACTGCAACTTTATATGGAGATTCAATTGGCATCACAAATGGCAGAAAAG   3059
Sulv1       CAGGCTTTTGGACTGCAACTTTATATGGAGATTCAATTGGCATCACAAATGGCAGAAAAG   3055
Jilv7       CAGGCTTTTGGACTGCAACTTTATATGGAGATTCAATTGGCATCACAAATGGCAGAAAAG   3055
VC1973A     CAGGCTTTTGGACTGCAACTTTATATGGAGATTCAATTGGCATCACAAATGGCAGAAAAG   3055
            ************************************************************

ACC41       AGAATGCTTAAAACTCAAACCTTACTGTGTGACATGCTCCTTCGTGATGCACCATTCGGC   3078
KPS2        AGAATGCTTAAAACTCAAACCTTACTGTGTGACATGCTCCTTCGTGATGCACCATTCGGC   3119
Sulv1       AGAATGCTTAAAACTCAAACCTTACTGTGTGACATGCTCCTTCGTGATGCACCATTCGGC   3115
Jilv7       AGAATGCTTAAAACTCAAACCTTACTGTGTGACATGCTCCTTCGTGATGCACCATTCGGC   3115
VC1973A     AGAATGCTTAAAACTCAAACCTTACTGTGTGACATGCTCCTTCGTGATGCACCATTCGGC   3115
            ************************************************************

ACC41       ATTGTGACTCAGTCTCCAAGTATCATGGATCTTGTGAAGTGTGATGGGGCTGCCTTATTT   3138
KPS2        ATTGTGACTCAGTCTCCAAGTATCATGGATCTTGTGAAGTGTGATGGAGCTGCCTTATTT   3179
Sulv1       ATTGTGACTCAGTCTCCAAGTATCATGGATCTTGTGAAGTGTGATGGAGCTGCCTTATTT   3175
Jilv7       ATTGTGACTCAGTCTCCAAGTATCATGGATCTTGTGAAGTGTGATGGAGCTGCCTTATTT   3175
VC1973A     ATTGTGACTCAGTCTCCAAGTATCATGGATCTTGTGAAGTGTGATGGAGCTGCCTTATTT   3175
            *********************************************** ************
```

```
ACC41           TATGATGGAAATTGCTGGTTGTTAGGTACATCCCCAACTGAAGCACAGGTAAAAGACATT   3198
KPS2            TATGATGGAAATTGCTGGTTGTTAGGTACATCCCCAACTGAAGCACAGGTAAAAGACATT   3239
Sulv1           TATGATGGAAATTGCTGGTTGTTAGGTACATCCCCAACTGAAGCACAGGTAAAAGACATT   3235
Jilv7           TATGATGGAAATTGCTGGTTGTTAGGTACATCCCCAACTGAAGCACAGGTAAAAGACATT   3235
VC1973A         TATGATGGAAATTGCTGGTTGTTAGGTACATCCCCAACTGAAGCACAGGTAAAAGACATT   3235
                ************************************************************

ACC41           GCAGAATGGCTACTTAGTAATCATGGGGATTCAACAGGTTTGACAACAGATAGTTTAGCT   3258
KPS2            GCAGAATGGCTACTTAGTAATCATGGGGATTCAACAGGTTTGACAACAGATAGTTTAGCT   3299
Sulv1           GCAGAATGGCTACTTAGTAATCATGGGGATTCAACAGGTTTGACAACAGATAGTTTAGCT   3295
Jilv7           GCAGAATGGCTACTTAGTAATCATGGGGATTCAACAGGTTTGACAACAGATAGTTTAGCT   3295
VC1973A         GCAGAATGGCTACTTAGTAATCATGGGGATTCAACAGGTTTGACAACAGATAGTTTAGCT   3295
                ************************************************************

ACC41           GATGCTGGTTATCCAGGTGCTGCTTCACTGGGTGATGCGGTTTGTGGCATGGCCACGGCA   3318
KPS2            GATGCTGGTTATCCAGGTGCTGCTTCACTGGGTGATGCGGTTTGTGGCATGGCCACGGCA   3359
Sulv1           GATGCTGGTTATCCAGGTGCTGCTTCACTGGGTGATGCGGTTTGTGGCATGGCCACGGCA   3355
Jilv7           GATGCTGGTTATCCAGGTGCTGCTTCACTGGGTGATGCGGTTTGTGGCATGGCCACGGCA   3355
VC1973A         GATGCTGGTTATCCAGGTGCTGCTTCACTGGGTGATGCGGTTTGTGGCATGGCCACGGCA   3355
                ************************************************************

ACC41           AGAATCAATTCAAAACATTTCTTGTTCTGGTTCAGGTCTCACACTGCTAAGGAAGTCAGA   3378
KPS2            AGAATCAATTCAAAACATTTCTTGTTCTGGTTCAGGTCTCACACTGCTAAGGAAGTCAGA   3419
Sulv1           AGAATCAATTCAAAACATTTCTTGTTCTGGTTCAGGTCTCACACTGCTAAGGAAGTCAGA   3415
Jilv7           AGAATCAATTCAAAACATTTCTTGTTCTGGTTCAGGTCTCACACTGCTAAGGAAGTCAGA   3415
VC1973A         AGAATCAATTCAAAACATTTCTTGTTCTGGTTCAGGTCTCACACTGCTAAGGAAGTCAGA   3415
                ************************************************************

ACC41           TGGGGAGGAGCCAAGCACCATCCAGAGGATAGGGATGATGGAGGAAAAATGAACCCAAGA   3438
KPS2            TGGGGAGGAGCGAAGCACCATCCAGAGGATAGGGATGATGGAGGAAAAATGAACCCAAGA   3479
Sulv1           TGGGGAGGAGCGAAGCACCATCCAGAGGATAGGGATGATGGAGGAAAAATGAACCCAAGA   3475
Jilv7           TGGGGAGGAGCGAAGCACCATCCAGAGGATAGGGATGATGGAGGAAAAATGAACCCAAGA   3475
VC1973A         TGGGGAGGAGCGAAGCACCATCCAGAGGATAGGGATGATGGAGGAAAAATGAACCCAAGA   3475
                *********** ************************************************

ACC41           TCATCATTCAAAGCTTTTCTTGAAGTAGTCAAAAGCAAAAGTTTGCCTTGGGAAGTGTCA   3498
KPS2            TCATCATTCAAAGCTTTTCTTGAAGTAGTCAAAAGCAAAAGTTTGCCTTGGGAAGTGTCA   3539
Sulv1           TCATCATTCAAAGCTTTTCTTGAAGTAGTCAAAAGCAAAAGTTTGCCTTGGGAAGTGTCA   3535
Jilv7           TCATCATTCAAAGCTTTTCTTGAAGTAGTCAAAAGCAAAAGTTTGCCTTGGGAAGTGTCA   3535
VC1973A         TCATCATTCAAAGCTTTTCTTGAAGTAGTCAAAAGCAAAAGTTTGCCTTGGGAAGTGTCA   3535
                ************************************************************

ACC41           GAAATCAATGCTATTCACTCATTACAACTAATGATAAGAGATTCTTTCCAAGACACTGAG   3558
KPS2            GAAATCAATGCTATTCACTCATTACAACTAATGATAAGAGATTCTTTCCAGGACACTGAG   3599
Sulv1           GAAATCAATGCTATTCACTCATTACAACTAATGATAAGAGATTCTTTCCAGGACACTGAG   3595
Jilv7           GAAATCAATGCTATTCACTCATTACAACTAATGATAAGAGATTCTTTCCAGGACACTGAG   3595
VC1973A         GAAATCAATGCTATTCACTCATTACAACTAATGATAAGAGATTCTTTCCAGGACACTGAG   3595
                ************************************************** *********

ACC41           ATCACCGATCCAAAGACTTCAAACTATGTGCAGAAAACTGACACTTCAACTGGAGCGATG   3618
KPS2            ATAACCGGTCCAAAGACTTCAAACTATGTGCAGAAAACTGACACTTCAACTGGAGCGATG   3659
Sulv1           ATAACCGGTCCAAAGACTTCAAACTATGTGCAGAAAACTGACACTTCAACTGGAGCGATG   3655
Jilv7           ATAACCGGTCCAAAGACTTCAAACTATGTGCAGAAAACTGACACTTCAACTGGAGCGATG   3655
VC1973A         ATAACCGGTCCAAAGACTTCAAACTATGTGCAGAAAACTGACACTTCAACTGGAGCGATG   3655
                ** **** ****************************************************

ACC41           GATGAACTCAGTTCAGTAGCACTTGAAATGGTGAGATTAATCGAGACTGCAACAGTTCCG   3678
KPS2            GATGAACTCAGTTCAGTAACACTTGAAATGGTGAGATTAATCGAGACTGCAACAGTTCCG   3719
Sulv1           GATGAACTCAGTTCAGTAGCACTTGAAATGGTGAGATTAATCGAGACTGCAACAGTTCCG   3715
Jilv7           GATGAACTCAGTTCAGTAGCACTTGAAATGGTGAGATTAATCGAGACTGCAACAGTTCCG   3715
VC1973A         GATGAACTCAGTTCAGTAGCACTTGAAATGGTGAGATTAATCGAGACTGCAACAGTTCCG   3715
                ****************** *****************************************

ACC41           ATTTTCGGGGTTGATTCAGGTGGGGTAATCAATGGATGGAATTCAAAGATTGCTGAACTG   3738
KPS2            ATTTTCGGGGTTGATTCAGATGGGGTAATCAATGGATGGAATTCAAAGATTGCTGAACTG   3779
Sulv1           ATTTTCGGGGTTGATTCAGATGGGGTAATCAATGGATGGAATTCAAAGATTGCTGAACTG   3775
Jilv7           ATTTTCGGGGTTGATTCAGATGGGGTAATCAATGGATGGAATTCAAAGATTGCTGAACTG   3775
VC1973A         ATTTTCGGGGTTGATTCAGATGGGGTAATCAATGGATGGAATTCAAAGATTGCTGAACTG   3775
                ******************* ****************************************

ACC41           ACAGGTCTACAAGGCAGTGAAGCTATGGGGAAATCCATGGTAAATGAAATTATACATGCG   3798
KPS2            ACAGGTCTACAAGGCAGTGAAGCTATGGGGAAATCCATGGTAAATGAAATTATACATGCG   3839
Sulv1           ACAGGTCTACAAGGCAGTGAAGCTATGGGGAAATCCATGGTAAATGAAATTATACATGCG   3835
```

```
Jilv7          ACAGGTCTACAAGGCAGTGAAGCTATGGGAAATCCATGGTAAATGAAATTATACATGCG   3835
VC1973A        ACAGGTCTACAAGGCAGTGAAGCTATGGGAAATCCATGGTAAATGAAATTATACATGCG   3835
               ***********************************************************

ACC41          GACTCATGTGACACTTTTAAAAATACTCTAAGCAGAGCCTTGCAAGGTAAATCAGTTGTT   3858
KPS2           GACTCATGTGACACTTTTAAAAATACTCTAAGCAGAGCCTTGCAAGGTAAATCAGTTATT   3899
Sulv1          GACTCATGTGACACTTTTAAAAATACTCTAAGCAGAGCCTTGCAAGGTAAATCAGTTATT   3895
Jilv7          GACTCATGTGACACTTTTAAAAATACTCTAAGCAGAGCCTTGCAAGGTAAATCAGTTATT   3895
VC1973A        GACTCATGTGACACTTTTAAAAATACTCTAAGCAGAGCCTTGCAAGGTAAATCAGTTATT   3895
               ********************************************************* **

ACC41          GTAGAAAAGATAGTAGTAGATTTAGAGTGTTCACTTCATTAACTCTCAAATGTTATTAAC   3918
KPS2           GTAGAAAAGATAGTGGTAGATTTAGAGTGTTCACTTCATTAACTCTCAAATGTTATTAAC   3959
Sulv1          GTAGAAAAGATAGTGGTAGATTTAGAGTGTTCACTTCATTAACTCTCAAATGTTATTAAC   3955
Jilv7          GTAGAAAAGATAGTGGTAGATTTAGAGTGTTCACTTCATTAACTCTCAAATGTTATTAAC   3955
VC1973A        GTAGAAAAGATAGTGGTAGATTTAGAGTGTTCACTTCATTAACTCTCAAATGTTATTAAC   3955
               ************** *********************************************

ACC41          AATTCATTGTGGGGGAAAAAACGTTCTGTGTAGGTCTTTTAGAATTTGATTCCACATTGT   3978
KPS2           AATTCATTGTGGGG-AAAAAACGTTCTGTGTAGGTCTTTTAGAATTTGATTCCACATTGT   4018
Sulv1          AATTCATTGTGGGG-AAAAAACGTTCTGTGTAGGTCTTTTAGAATTTGATTCCACATTGT   4014
Jilv7          AATTCATTGTGGGG-AAAAAACGTTCTGTGTAGGTCTTTTAGAATTTGATTCCACATTGT   4014
VC1973A        AATTCATTGTGGGG-AAAAAACGTTCTGTGTAGGTCTTTTAGAATTTGATTCCACATTGT   4014
               ************** *********************************************

ACC41          TTTGAAATATTCAATTCATGTACTATATTGGACAATCAAATATTCATGTTTTTTTAGGTT   4038
KPS2           TTTGAAATATTCAATTCATGTACTATATTGGACAATCAAATATTCATGTTTTTTTAGGTT   4078
Sulv1          TTTGAAATATTCAATTCATGTACTATATTGGACAATCAAATATTCATGTTTTTTTAGGTT   4074
Jilv7          TTTGAAATATTCAATTCATGTACTATATTGGACAATCAAATATTCATGTTTTTTTAGGTT   4074
VC1973A        TTTGAAATATTCAATTCATGTACTATATTGGACAATCAAATATTCATGTTTTTTTAGGTT   4074
               ************************************************************

ACC41          GTGCTATTATTTTCCCATTGGAGTATAAAATTTAATGTCTAGTCTTATGTAATTGAATAT   4098
KPS2           GTGCTATTATTTTCCCATTGGAGTATAAAATTTAATGTCTAGTCTTATGATATTGAATAT   4138
Sulv1          GTGCTATTATTTTCCCATTGGAGTATAAAATTTAATGTCTAGTCTTATGTAATTGAATAT   4134
Jilv7          GTGCTATTATTTTCCCATTGGAGTATAAAATTTAATGTCTAGTCTTATGTAATTGAATAT   4134
VC1973A        GTGCTATTATTTTCCCATTGGAGTATAAAATTTAATGTCTAGTCTTATGTAATTGAATAT   4134
               *************************************************  *********

ACC41          GTAAGATAATGTTTTAGTTGCAGTCTTATTTTGATTCGTTCTCCCTGAAAGTTAAGTACT   4158
KPS2           GTAAGATAATGTTTCAGTTGCAGTCTTATTTTGATTCGTTCTCCCTGAAAGTTAAGTACT   4198
Sulv1          GTAAGATAATGTTTCAGTTGCAGTCTTATTTTGATTCGTTCTCCCTGAAAGTTAAGTACT   4194
Jilv7          GTAAGATAATGTTTCAGTTGCAGTCTTATTTTGATTCGTTCTCCCTGAAAGTTAAGTACT   4194
VC1973A        GTAAGATAATGTTTCAGTTGCAGTCTTATTTTGATTCGTTCTCCCTGAAAGTTAAGTACT   4194
               ************** *********************************************

ACC41          CATAGAAATGAATTGTAGTTTTTGCAGGAGAGGAGGACAAAAGTGTTGAGTTGAAAATAA   4218
KPS2           CATAGAAATGAATTGTAGTTTTTGCAGGAGAGGAGGACAAAAGTGTTGAGTTGAAAATAA   4258
Sulv1          CATAGAAATGAATTGTAGTTTTTGCAGGAGAGGAGGACAAAAGTGTTGAGTTGAAAATAA   4254
Jilv7          CATAGAAATGAATTGTAGTTTTTGCAGGAGAGGAGGACAAAAGTGTTGAGTTGAAAATAA   4254
VC1973A        CATAGAAATGAATTGTAGTTTTTGCAGGAGAGGAGGACAAAAGTGTTGAGTTGAAAATAA   4254
               ************************************************************

ACC41          AGCACTTTGGGATTCAACAGCAACAGAAAGTTGTATATCTCATGGTCAATGCTTGCACTA   4278
KPS2           AGCACTTTGGGATTCAACAGCAACAGAAAGTTGTATATCTCATGGTCAATGCTTGCACTA   4318
Sulv1          AGCACTTTGGGATTCAACAGCAACAGAAAGTTGTATATCTCATGGTCAATGCTTGCACTA   4314
Jilv7          AGCACTTTGGGATTCAACAGCAACAGAAAGTTGTATATCTCATGGTCAATGCTTGCACTA   4314
VC1973A        AGCACTTTGGGATTCAACAGCAACAGAAAGTTGTATATCTCATGGTCAATGCTTGCACTA   4314
               ************************************************************

ACC41          GTAGGGACTACACAGATTCTGTTGTTGGGGTATGCTTTGTAGGTAAAGACATCACTTATG   4338
KPS2           GTAGGGACTACACAGATTCTGTTGTTGGGGTATGCTTTGTAGGTAAAGACATCACTTATG   4378
Sulv1          GTAGGGACTACACAGATTCTGTTGTTGGGGTATGCTTTGTAGGTAAAGACATCACTTATG   4374
Jilv7          GTAGGGACTACACAGATTCTGTTGTTGGGGTATGCTTTGTAGGTAAAGACATCACTTATG   4374
VC1973A        GTAGGGACTACACAGATTCTGTTGTTGGGGTATGCTTTGTAGGTAAAGACATCACTTATG   4374
               ************************************************************

ACC41          AGAAAATGGTTCAAGATAAATTCATCAAGTTGGAAGGTGATTACAAAGCAATCGTGCAGA   4398
KPS2           AGAAAATGGTTCAAGATAAATTCATCAAGTTGGAAGGTGATTACAAAACAATCGTGCAAA   4438
Sulv1          AGAAAATGGTTCAAGATAAATTCATCAAGTTGGAAGGTGATTACAAAACAATCGTGCAAA   4434
Jilv7          AGAAAATGGTTCAAGATAAATTCATCAAGTTGGAAGGTGATTACAAAACAATCGTGCAAA   4434
VC1973A        AGAAAATGGTTCAAGATAAATTCATCAAGTTGGAAGGTGATTACAAAACAATCGTGCAAA   4434
               *********************************************** ********** *
```

```
ACC41      GTCTCAATCCACTGATTCCACCCATATTTTCTTCTGATGAGAGTGCCTGCTGTTCTGAAT   4458
KPS2       GTCTCAGTCCACTTATTCCACCCATATTTTCTTCTGACGAGAGTGCATGCTGTTCTGAAT   4498
Sulv1      GTCTCAGTCCACTTATTCCACCCATATTTTCTTCTGACGAGAGTGCATGCTGTTCTGAAT   4494
Jilv7      GTCTCAGTCCACTTATTCCACCCATATTTTCTTCTGACGAGAGTGCATGCTGTTCTGAAT   4494
VC1973A    GTCTCAGTCCACTTATTCCACCCATATTTTCTTCTGACGAGAGTGCATGCTGTTCTGAAT   4494
           ****** ****** *********************** ******** *************

ACC41      GGAATGCAGCCATGGAAAGGCTAACTGGTTGGAAAAGAGATGAAGTCATAGGAAAATTGC   4518
KPS2       GGAATGCAGCCATGGAAAGACTAACAGGTTGGAAAAGAGATGAAGTCATAGGAAAATTGC   4558
Sulv1      GGAATGCAGCCATGGAAAGACTAACAGGTTGGAAAAGAGATGAAGTCATAGGAAAATTGC   4554
Jilv7      GGAATGCAGCCATGGAAAGACTAACAGGTTGGAAAAGAGATGAAGTCATAGGAAAATTGC   4554
VC1973A    GGAATGCAGCCATGGAAAGACTAACAGGTTGGAAAAGAGATGAAGTCATAGGAAAATTGC   4554
           ******************* ***** **********************************

ACC41      TTCCTGGTGAAATATTTGGAAGTTTTTGTCGATTAAAAGGTCAAGATACCCTGACTAATT   4578
KPS2       TTCCTGGTGAAATATTTGGAAGTTTTTGTCGAATAAAAGGTCAAGATACCCTGACTAATT   4618
Sulv1      TTCCTGGTGAAATATTTGGAAGTTTTTGTCGACTAAAAGGTCAAGATACCCTGACTAATT   4614
Jilv7      TTCCTGGTGAAATATTTGGAAGTTTTTGTCGACTAAAAGGTCAAGATACCCTGACTAATT   4614
VC1973A    TTCCTGGTGAAATATTTGGAAGTTTTTGTCGACTAAAAGGTCAAGATACCCTGACTAATT   4614
           ******************************** ***************************

ACC41      TTATGATTTAATATACCGTGGAATAAGTGGTCAAAATTCTGAGAAGTTACCATTTGGAT   4638
KPS2       TTATGATTTAATATACCGTGGAATAAGTGGTCAAGATTCTGAGAAGTTACCATTTGGAT   4678
Sulv1      TTATGATTTAATATACCGTGGAATAAGTGGTCAAGATTCTGAGAAGTTACCATTTGGAT   4674
Jilv7      TTATGATTTAATATACCGTGGAATAAGTGGTCAAGATTCTGAGAAGTTACCATTTGGAT   4674
VC1973A    TTATGATTTAATATACCGTGGAATAAGTGGTCAAGATTCTGAGAAGTTACCATTTGGAT   4674
           ********************************** *************************

ACC41      TTTTTGATAGAAATGGAGAATTTGTTGAGAGTTATATAACAGCAAACAAGAGAATTGATG   4698
KPS2       TTTTTGATAGAAATGGAGAATTTGTTGAGAGTTATATAACAGCAAACAAGAGAATTGATG   4738
Sulv1      TTTTTGATAGAAATGGAGAATTTGTTGAGAGTTATATAACAGCAAACAAGAGAATTGATG   4734
Jilv7      TTTTTGATAGAAATGGAGAATTTGTTGAGAGTTATATAACAGCAAACAAGAGAATTGATG   4734
VC1973A    TTTTTGATAGAAATGGAGAATTTGTTGAGAGTTATATAACAGCAAACAAGAGAATTGATG   4734
           ************************************************************

ACC41      CTGGTGGGAATATACTTGGCTGTTTTTGCTTCATGCAGGTTGTAACACCAGACCTGAATC   4758
KPS2       CTGGTGGGAATATACTTGGCTGTTTTTGCTTCATGCAGGTTGTAACACCAGACCTGAATC   4798
Sulv1      CTGGTGGGAATATACTTGGCTGTTTTTGCTTCATGCAGGTTGTAACACCAGACCTGAATC   4794
Jilv7      CTGGTGGGAATATACTTGGCTGTTTTTGCTTCATGCAGGTTGTAACACCAGACCTGAATC   4794
VC1973A    CTGGTGGGAATATACTTGGCTGTTTTTGCTTCATGCAGGTTGTAACACCAGACCTGAATC   4794
           ************************************************************

ACC41      AGTCCTCCGAAGAACACAAGCCAAGAGGCAGAGAAAACATTTCTGAATCCGAAGAGTTGG   4818
KPS2       ACTCCTCTGAAGAACACAAGCCAAGAGGCAGAGAAAACATTTCTGAATCCGAAGAGTTGG   4858
Sulv1      ACTCCTCTGAAGAACACAAGCCAAGAGGCAGAGAAAACATTTCTGAATCCGAAGAGTTGG   4854
Jilv7      ACTCCTCTGAAGAACACAAGCCAAGAGGCAGAGAAAACATTTCTGAATCCGAAGAGTTGG   4854
VC1973A    ACTCCTCTGAAGAACACAAGCCAAGAGGCAGAGAAAACATTTCTGAATCCGAAGAGTTGG   4854
           * ***** ****************************************************

ACC41      CTTATATACTACAAGAGATGAAGAAACCTTTAAATGGCATACGATTCACGCACAAACTTT   4878
KPS2       CTTATATACTACAAGAGATGAAGAAACCTTTAAATGGCATACGATTCACGCACAAACTTT   4918
Sulv1      CTTATATACTACAAGAGATGAAGAAACCTTTAAATGGCATACGATTCACGCACAAACTTT   4914
Jilv7      CTTATATACTACAAGAGATGAAGAAACCTTTAAATGGCATACGATTCACGCACAAACTTT   4914
VC1973A    CTTATATACTACAAGAGATGAAGAAACCTTTAAATGGCATACGATTCACGCACAAACTTT   4914
           ************************************************************

ACC41      TGGAAAGTACAACTGTTTCAGAAAACCAAAAACAATTTCTTGACACCAGTGATGCATGTG   4938
KPS2       TGGAAAGTACAACTGTTTCAGAAAACCAAAAACAATTTCTTGACACCAGTGATGCATGTG   4978
Sulv1      TGGAAAGTACAACTGTTTCAGAAAACCAAAAACAATTTCTTGACACCAGTGATGCATGTG   4974
Jilv7      TGGAAAGTACAACTGTTTCAGAAAACCAAAAACAATTTCTTGACACCAGTGATGCATGTG   4974
VC1973A    TGGAAAGTACAACTGTTTCAGAAAACCAAAAACAATTTCTTGACACCAGTGATGCATGTG   4974
           ************************************************************

ACC41      AAAGACAAATCATGGCAATTATTGAGGATACAAATTTAGGAAGTATTAATGAAGGGTAAG   4998
KPS2       AAAGACAAATCATGGCAATTATTGAGGATACAAATTTAGGAAGTATTAATGAAGGGTAAG   5038
Sulv1      AAAGACAAATCATGGCAATTATTGAGGATACAAATTTAGGAAGTATTAATGAAGGGTAAG   5034
Jilv7      AAAGACAAATCATGGCAATTATTGAGGATACAAATTTAGGAAGTATTAATGAAGGGTAAG   5034
VC1973A    AAAGACAAATCATGGCAATTATTGAGGATACAAATTTAGGAAGTATTAATGAAGGGTAAG   5034
           ************************************************************

ACC41      AGTTTTTGTTGTGTTTTGGTAGGCTGAAATTCTCTCTTGGAGTAGTACTTTCCTTTTTCG   5058
KPS2       AGTTTTTGTTGTGTTTTGGTAGGCTGAAATTCTCTCTTGGAGTAGTACTTTCCTTTTTCG   5098
Sulv1      AGTTTTTGTTGTGTTTTGGTAGGCTGAAATTCTCTCTTGGAGTAGTACTTTCCTTTTTCG   5094
Jilv7      AGTTTTTGTTGTGTTTTGGTAGGCTGAAATTCTCTCTTGGAGTAGTACTTTCCTTTTTCG   5094
```

```
VC1973A         AGTTTTTGTTGTGTTTTGGTAGGCTGAAATTCTCTCTTGGAGTAGTACTTTCCTTTTTCG   5094
                ************************************************************

ACC41           TCCTCCGATTATACACTGATATTCAATAACACTGACTTTATGTTTCATATCTAATGCTAA   5118
KPS2            TCCTCCGATTATACACTGATATTCAATAACACTGACTTTATGTTTCATATCTTATGCTAA   5158
Sulv1           TCCTCCGATTATACACTGATATTCAATAACACTGACTTTATGTTTCATATCTTATGCTAA   5154
Jilv7           TCCTCCGATTATACACTGATATTCAATAACACTGACTTTATGTTTCATATCTTATGCTAA   5154
VC1973A         TCCTCCGATTATACACTGATATTCAATAACACTGACTTTATGTTTCATATCTTATGCTAA   5154
                **************************************************** *******

ACC41           TGCTAGCAGCACTTTGCAGCTAAACATGGAAGAATTTGTTCTGGGAAATATTTTAGATGC   5178
KPS2            TGCTAGCAGCACTTTGCAGCTAAACATGGAAGAATTTGTTCTGGGAAATATTTTAGATGC   5218
Sulv1           TGCTAGCAGCACTTTGCAGCTAAACATGGAAGAATTTGTTCTGGGAAATATTTTAGATGC   5214
Jilv7           TGCTAGCAGCACTTTGCAGCTAAACATGGAAGAATTTGTTCTGGGAAATATTTTAGATGC   5214
VC1973A         TGCTAGCAGCACTTTGCAGCTAAACATGGAAGAATTTGTTCTGGGAAATATTTTAGATGC   5214
                ************************************************************

ACC41           CATTGTCAGTCAAGTCATGATATTGATAAGAGAAAAGAACTTGCAACTGTTTCATGAAAT   5238
KPS2            CATTGTCAGTCAAGTCATGATATTGATAAGAGAAAAGAACTTGCAACTGTTTCATGAAAT   5278
Sulv1           CATTGTCAGTCAAGTCATGATATTGATAAGAGAAAAGAACTTGCAACTGTTTCATGAAAT   5274
Jilv7           CATTGTCAGTCAAGTCATGATATTGATAAGAGAAAAGAACTTGCAACTGTTTCATGAAAT   5274
VC1973A         CATTGTCAGTCAAGTCATGATATTGATAAGAGAAAAGAACTTGCAACTGTTTCATGAAAT   5274
                ************************************************************

ACC41           TCCTGATGAAATAAAAGTGCTTTCTTTATATGGTGACCAAATTAGGCTTCAGGTGGTCTT   5298
KPS2            TCCTGATGAAATAAAAGTGCTTTCTTTATATGGTGACCAAATTAGGCTTCAGGTGGTCTT   5338
Sulv1           TCCTGATGAAATAAAAGTGCTTTCTTTATATGGTGACCAAATTAGGCTTCAGGTGGTCTT   5334
Jilv7           TCCTGATGAAATAAAAGTGCTTTCTTTATATGGTGACCAAATTAGGCTTCAGGTGGTCTT   5334
VC1973A         TCCTGATGAAATAAAAGTGCTTTCTTTATATGGTGACCAAATTAGGCTTCAGGTGGTCTT   5334
                ************************************************************

ACC41           GTCTGACTTTTTGCTTAATGTAGTCAGCCATACACCATCTCCAAATGGCTGGGTAGAAAT   5358
KPS2            GTCTGACTTTTTGCTTAATGTAGTCAGACATACACCATCTCCAAATGGCTGGGTAGAAAT   5398
Sulv1           GTCTGACTTTTTGCTTAATGTAGTCAGACATACACCATCTCCAAATGGCTGGGTAGAAAT   5394
Jilv7           GTCTGACTTTTTGCTTAATGTAGTCAGACATACACCATCTCCAAATGGCTGGGTAGAAAT   5394
VC1973A         GTCTGACTTTTTGCTTAATGTAGTCAGACATACACCATCTCCAAATGGCTGGGTAGAAAT   5394
                *************************** ********************************

ACC41           CAAGATTTCACCTGGTTTAAAGAGAATACAGGATGGCAATGAGTTTATCCATCTGAAGTT   5418
KPS2            CAAGATTTCACCTGGTTTAAAGATAATACAGGATGGCAATGAGTTTATCCATCTGAAGTT   5458
Sulv1           CAAGATTTCACCTGGTTTAAAGATAATACAGGATGGCAATGAGTTTATCCATCTGAAGTT   5454
Jilv7           CAAGATTTCACCTGGTTTAAAGATAATACAGGATGGCAATGAGTTTATCCATCTGAAGTT   5454
VC1973A         CAAGATTTCACCTGGTTTAAAGATAATACAGGATGGCAATGAGTTTATCCATCTGAAGTT   5454
                ********************** *************************************

ACC41           CAGGTACAAAAAACCAAGAATTTATCACTGTTATCTTCTGGGGATTTACCAATTTGGATTC   5478
KPS2            CAGGTACAAAAAACCAAGAATTTATCACTGTTATCTTCTGGGGATTTACCAATTTGGATTC   5518
Sulv1           CAGGTACAAAAAACCAAGAATTTATCACTGTTATCTTCTGGGGATTTACCAATTTGGATTC   5514
Jilv7           CAGGTACAAAAAACCAAGAATTTATCACTGTTATCTTCTGGGGATTTACCAATTTGGATTC   5514
VC1973A         CAGGTACAAAAAACCAAGAATTTATCACTGTTATCTTCTGGGGATTTACCAATTTGGATTC   5514
                ************************************************************

ACC41           TCTGCTGGGTTTTTCTGGCTGTTTCTCTGTTGTTCCCTCAAACTACACTGATGGACATTG   5538
KPS2            TCTGCTGGGTTTTTCTGGCTGTTTCTCTGTTGTTCCCTCAAACTACACTGATGGACATTG   5578
Sulv1           TCTGCTGGGTTTTTCTGGCTGTTTCTCTGTTGTTCCCTCAAACTACACTGATGGACATTG   5574
Jilv7           TCTGCTGGGTTTTTCTGGCTGTTTCTCTGTTGTTCCCTCAAACTACACTGATGGACATTG   5574
VC1973A         TCTGCTGGGTTTTTCTGGCTGTTTCTCTGTTGTTCCCTCAAACTACACTGATGGACATTG   5574
                ************************************************************

ACC41           ATTTTGAGATTTTAAAATATATTAAAAAGATTTTTAAAGTACTAACATCAGTGTATTTTA   5598
KPS2            ATTTTGAGATTTTAAAATATATTAAAAAGATTTTTAAAGTACTAACATCAGTGTATTTTA   5638
Sulv1           ATTTTGAGATTTTAAAATATATTAAAAAGATTTTTAAAGTACTAACATCAGTGTATTTTA   5634
Jilv7           ATTTTGAGATTTTAAAATATATTAAAAAGATTTTTAAAGTACTAACATCAGTGTATTTTA   5634
VC1973A         ATTTTGAGATTTTAAAATATATTAAAAAGATTTTTAAAGTACTAACATCAGTGTATTTTA   5634
                ************************************************************

ACC41           ATGTTCAACAGAGGACAACATTAAAAAACCCTCTAGAGAATGTGAATCATTCTATACTTT   5658
KPS2            ATGTTCAACAGAG--------------------TAGAGAATGTGAATCATTCTATACTTT   5678
Sulv1           ATGTTCAACAGAG--------------------TAGAGAATGTGAATCATTCTATACTTT   5674
Jilv7           ATGTTCAACAGAG--------------------TAGAGAATGTGAATCATTCTATACTTT   5674
VC1973A         ATGTTCAACAGAG--------------------TAGAGAATGTGAATCATTCTATACTTT   5674
                *************                    ***************************

ACC41           CTACATGGGGATAGTTGATTTCTCCCATTTTTGTTATACTTGAAATTTATGCCTGAGTAG   5718
```

```
KPS2          CTACATGGGGATAGTTGATTTCTACCATTTTTGTTATACTTGAAATTTATGCCTGAGTAG   5738
Sulv1         CTACATGGGGATAGTTGATTTCTACCATTTTTGTTATACTTGAAATTTATGCCTGAGTAG   5734
Jilv7         CTACATGGGGATAGTTGATTTCTACCATTTTTGTTATACTTGAAATTTATGCCTGAGTAG   5734
VC1973A       CTACATGGGGATAGTTGATTTCTACCATTTTTGTTATACTTGAAATTTATGCCTGAGTAG   5734
              *********************** ************************************

ACC41         CTGTGGCTTTATGTTTAACACCTTTTGACAAGGTGAGACAAACTTTGAAGATATACAAGA   5778
KPS2          CTGTGGCTTTATGTTTAACACCTTTTGACAAGGTGAGACAAACTTTGAAGATATACAAGA   5798
Sulv1         CTGTGGCTTTATGTTTAACACCTTTTGACAAGGTGAGACAAACTTTGAAGATATACAAGA   5794
Jilv7         CTGTGGCTTTATGTTTAACACCTTTTGACAAGGTGAGACAAACTTTGAAGATATACAAGA   5794
VC1973A       CTGTGGCTTTATGTTTAACACCTTTTGACAAGGTGAGACAAACTTTGAAGATATACAAGA   5794
              ************************************************************

ACC41         GTTGTCACAAACTTTGAGTAGTTGTGATCTTACGTTTTAGAAACTGACATAGTTTTAGGC   5838
KPS2          GTTGTCACAAACTTTGAGTAGTTGTGATCTTACGTTTTAGAAACTGACATAGTTTTAGGC   5858
Sulv1         GTTGTCACAAACTTTGAGTAGTTGTGATCTTACGTTTTAGAAACTGACATAGTTTTAGGC   5854
Jilv7         GTTGTCACAAACTTTGAGTAGTTGTGATCTTACGTTTTAGAAACTGACATAGTTTTAGGC   5854
VC1973A       GTTGTCACAAACTTTGAGTAGTTGTGATCTTACGTTTTAGAAACTGACATAGTTTTAGGC   5854
              ************************************************************

ACC41         TACAAGTGTTGTGATGGTTACTTGAAGATGTCACCTATGTTAGATTTCTGTGTAGACTAG   5898
KPS2          TACAAGTGTTGTGATGGTTACTTGAAGATGTCGCCTATGTTAGATTACTGTGTAGACTAG   5918
Sulv1         TACAAGTGTTGTGATGGTTACTTGAAGATGTCGCCTATGTTAGATTACTGTGTAGACTAG   5914
Jilv7         TACAAGTGTTGTGATGGTTACTTGAAGATGTCGCCTATGTTAGATTACTGTGTAGACTAG   5914
VC1973A       TACAAGTGTTGTGATGGTTACTTGAAGATGTCGCCTATGTTAGATTACTGTGTAGACTAG   5914
              ******************************** ************* *************

ACC41         AGGCTCATTTGATCATATATTTTTTTTGCAGAATGACCCATTCTGGTCAAGGTCTTCCTT   5958
KPS2          AGGCTCATTTGATCATATATTCTTCTTGCAGAATGACCCATTCTGGTCAAGGTCTTCCTT   5978
Sulv1         AGGCTCATTTGATCATATATTCTTCTTGCAGAATGACCCATTCTGGTCAAGGTCTTCCTT   5974
Jilv7         AGGCTCATTTGATCATATATTCTTCTTGCAGAATGACCCATTCTGGTCAAGGTCTTCCTT   5974
VC1973A       AGGCTCATTTGATCATATATTCTTCTTGCAGAATGACCCATTCTGGTCAAGGTCTTCCTT   5974
              ********************* ** ***********************************

ACC41         CAGATGTTTTTCATGATATGTTTGAAGGAGGGAATCAATGGAATACACAGGAAGGTGTAG   6018
KPS2          CAGATGTTCTTCATGATATGTTTGAAGGAGGGAATCAATGGAATACGCAGGAAGGTCTAG   6038
Sulv1         CAGATGTTCTTCATGATATGTTTGAAGGAGGGAATCAATGGAATACGCAGGAAGGTCTAG   6034
Jilv7         CAGATGTTCTTCATGATATGTTTGAAGGAGGGAATCAATGGAATACGCAGGAAGGTCTAG   6034
VC1973A       CAGATGTTCTTCATGATATGTTTGAAGGAGGGAATCAATGGAATACGCAGGAAGGTCTAG   6034
              ******** ************************************* ********* ***

ACC41         GACTATACATGTCCAGAAAAAATCCTCAGTAGGATGAGTGGTCATGTCCACTACGTAAGAG   6078
KPS2          GACTATACATGTCCAAAAAAAATCCTCAGTAGGATGAGTGGTCATGTCCACTACGTAAGAG   6098
Sulv1         GACTATACATGTCCAAAAAAAATCCTCAGTAGGATGAGTGGTCATGTCCACTACGTAAGAG   6094
Jilv7         GACTATACATGTCCAAAAAAAATCCTCAGTAGGATGAGTGGTCATGTCCACTACGTAAGAG   6094
VC1973A       GACTATACATGTCCAAAAAAAATCCTCAGTAGGATGAGTGGTCATGTCCACTACGTAAGAG   6094
              *************** *********************************************

ACC41         AACAAAATAAGTGTTATTTTCTCATTGATCTTGACACTAGAACAAGAAAAAAAAGACAAA   6138
KPS2          AACAAAATAAGTGTTATTTTCTCATTGATCTTGACATTAGAACAAGAAAAGAAAGGCAAA   6158
Sulv1         AACAAAATAAGTGTTATTTTCTCATTGATCTTGACATTAGAACAAGAAAAGAAAGGCAAA   6154
Jilv7         AACAAAATAAGTGTTATTTTCTCATTGATCTTGACATTAGAACAAGAAAAGAAAGGCAAA   6154
VC1973A       AACAAAATAAGTGTTATTTTCTCATTGATCTTGACATTAGAACAAGAAAAGAAAGGCAAA   6154
              ************************************ ************* **** ****

ACC41         AGAGTTTGCATACAGAGACAAGCATGTTAAGATAGTTTTTTTCTCTTGATGCTCTTTCTA   6198
KPS2          AGAGTTTGCATGCAGAGACAAGCATGTTAAGCTAGTTTTTTTCTCTTGATGCTCTTACTA   6218
Sulv1         AGAGTTTGCATGCAGAGACAAGCATGTTAAGCTAGTTTTTTTCTCTTGATGCTCTTACTA   6214
Jilv7         AGAGTTTGCATGCAGAGACAAGCATGTTAAGCTAGTTTTTTTCTCTTGATGCTCTTACTA   6214
VC1973A       AGAGTTTGCATGCAGAGACAAGCATGTTAAGCTAGTTTTTTTCTCTTGATGCTCTTACTA   6214
              *********** ******************* ************************ ***

ACC41         GTGAAAGGGGTGTGAAGGGTGGTTTGCAAAGAAAGGTCTCTAATCTTGCTTTGAAGCTTG   6258
KPS2          GTGAAAGGGGTGTGAAGGGTGGTTTGCAAAGAGAGGTCTCTAATCTTGCTTTGAAGCTTG   6278
Sulv1         GTGAAAGGGGTGTGAAGGGTGGTTTGCAAAGAGAGGTCTCTAATCTTGCTTTGAAGCTTG   6274
Jilv7         GTGAAAGGGGTGTGAAGGGTGGTTTGCAAAGAGAGGTCTCTAATCTTGCTTTGAAGCTTG   6274
VC1973A       GTGAAAGGGGTGTGAAGGGTGGTTTGCAAAGAGAGGTCTCTAATCTTGCTTTGAAGCTTG   6274
              ******************************** ***************************

ACC41         GCAGATATATGGCACCTGCTCATGTCAACACTGGTATATCATCCTATTACACTACAAGGA   6318
KPS2          GCAGATATATGGCAACTGCTCATGTCAACACTGGTATATCATCCTATTACACTACAAGGA   6338
Sulv1         GCAGATATATGGCAACTGCTCATGTCAACACTGGTATATCATCCTATTACACTACAAGGA   6334
Jilv7         GCAGATATATGGCAACTGCTCATGTCAACACTGGTATATCATCCTATTACACTACAAGGA   6334
VC1973A       GCAGATATATGGCAACTGCTCATGTCAACACTGGTATATCATCCTATTACACTACAAGGA   6334
```

```
                   ************** *********************************************

ACC41              AGTTTATCTTATATATATATATATATATATATATTAGATACACACACACCCCC-TTTTTA   6377
KPS2               AGTTTATGCTTAT-----------ATATATATATTAGATACATACACACCCCTTTTTTA-   6386
Sulv1              AGTTTATGCTTAT-----------ATATATATATTAGATACATACACACCCCTTTTTTA-   6382
Jilv7              AGTTTATGCTTAT-----------ATATATATATTAGATACATACACACCCCTTTTTTA-   6382
VC1973A            AGTTTATGCTTAT-----------ATATATATATTAGATACATACACACCCCTTTTTTTA   6383
                   *******   *            ***************** *********   ****

ACC41              GTTTGGCTTAAAAATATTACAGTAACACAGT       6408
KPS2               GTTTGGCTTAAAAATATTACAGTAACACAGT       6417
Sulv1              GTTTGGCTTAAAAATATTACAGTAACACAGT       6413
Jilv7              GTTTGGCTTAAAAATATTACAGTAACACAGT       6413
VC1973A            GTTTGGCTTAAAAATATTACAGTAACACAGT       6414
                   *******************************
```

* indicates conserved nucleotide among mungbean accessions.

Supplemental Fig. 1. Sequence alignment of promotor and genic regions of *VrPHYE* gene between wild mungbean (ACC41) and cultivated mungbeans (KPS2, Sulv1, Jilv7 and VC1973A). Promotor, 5’UTR, exonic and 3’UTR regions are highlighted in pink, green, yellow, and blue, respectively.
