# Supplementary material for: Narrowing down a major QTL region reveals Phytochrome E (PHYE) as the candidate gene controlling flowering time in mungbean (Vigna radiata)
Source: Breed Sci. 2024 Feb 29;74(2):83–92. doi: 10.1270/jsbbs.23036 (PMC11442112; doi:10.1270/jsbbs.23036)
Supplement: Supplementary file 2 — Supplemental Tables [file 74_083_s2.pdf]

doi: 10.1270/jsbbs.23036

Supplemental Table 1 Sequences of primers used in this study. Primers used for gene mapping showing polymorphisms between the mapping parents (Kamphaeng Saen 2 and ACC41) are bolded.

Primer name	Forward sequence	Reverse sequence	Usage
VrDf-SSR001	CCACACAAATGTGCATTGT	ATAGGCTAATGGACACAGTCTTTG	Gene mapping
VrDf-SSR002	CTTCACAATCTGTTGTACTTTCAA	CATAAACGGTAAAATGAGTAATAGA	Gene mapping
VrDf-SSR003	GAAACAAGTTCATAATTCCATAATG	CCTCTTACAAGGTCCATGGTAG	Gene mapping
VrDf-SSR004	ACTTCACAATCTGTTGTACTTTCA	CATAAACGGTAAAATGAGTAATAG	Gene mapping
**VrDf-SSR005**	**GACCATGACCCTTTATGTAACT**	**GTGCAATGAAAACTGCTTTG**	**Gene mapping**
VrDf-SSR006	GTTTCACATGCAGCGAC	ATGCAGAGGTGCAGATTG	Gene mapping
VrDf-SSR007	GTTCAAGAAAGAGCCACCT	CTGGTAAGGTTCAACACAAAC	Gene mapping
VrDf-SSR008	TCCTATCAAAATAGCTTATACAC	TGGTAACGGTGTTATCG	Gene mapping
VrDf-SSR009	CCAAGTGCAAGAAAACTCC	AGTCACAGTTACAACTGCAGAC	Gene mapping
VrDf-SSR010	CAAGTTGTCACTTTTATCATGG	GGTAAAGAACTGAGTGATCAACA	Gene mapping
VrDf-SSR011	CTTTATTGATAGGATCAAATTCTG	GTGAAAGTGATTTACACAAAAGT	Gene mapping
**VrDf-SSR012**	**GATGGAAGCATCACAAACA**	**GGCCAATACATCGTTTTCA**	**Gene mapping**
**VrDf-SSR013**	**GGAATTCCTGTGTCTCAAGC**	**GCCCATCTTTGTGAAATG**	**Gene mapping**
VrDf-SSR014	CGCCATGTGAATGAGAA	CTTAGTCCATACTGTCATATTTGAA	Gene mapping
VrDf-SSR015	TGGGATTCAAGCTATCACA	TCTCTAACCAGTAACGAAGATG	Gene mapping
VrDf-SSR016	AGAGATTAACCTGTAGATAATTTTCAG	ACATCGGAACCTGATTTG	Gene mapping
**VrDf-SSR017**	**GCAATTTTGTTCGACCACT**	**GCACACCCTGTTAGCAGC**	**Gene mapping**
VrDf-SSR018	CTCAGGACAAGTTATAATAAACAAC	GCCAGAGACAGATGTTGAGGAG	Gene mapping
**VrDf-SSR019**	**AGGCTTTAGCAACGTACTGC**	**GCACATATGCAACTTGTTAAGG**	**Gene mapping**
VrDf-SSR020	CGTCGCACTTAGCAAACC	CCATGATTGTTCTTGGGG	Gene mapping
**VrDf-SSR021**	**GTGCACGCACACACACA**	**CGATGTGATACTCCACATTTC**	**Gene mapping**
**VrDf-SSR022**	**CGGATTGTCCTATCCAAACT**	**GGAGAGGGTACAGCAGC**	**Gene mapping**
VrDf-SSR023	TCATGAATCCTATCATTTTGTGG	AGTGGTCCCCTCTCCCTAAA	Gene mapping
VrDf-SSR024	CACAGATCTTTGTATTGAGTATGAA	CCAGTTATTTCGAATATGGTG	Gene mapping
VrDf-SSR025	ACACCTTTCTATGTGCATGCTT	GCTGAATCTACAGAAAACCCAAA	Gene mapping
VrDf-SSR026	TTGATTCCGATAATAACTTGGTCA	CACGGTTATCTTCTAAACAAGACTTT	Gene mapping

VrDf-SSR027	CCGATAAAAACATTTTCTTAAGGTTC	TGTCACAAAGATAAAAGGTCGTG	Gene mapping
**VrDf-SSR028**	**CAGAACCAAAATCCCAGCAC**	**ACAACCTGAGGAAAGGCTGA**	**Gene mapping**
**VrDf-SSR029**	**ACACCTTTCTATGTGCATGCTT**	**GCTGAATCTACAGAAAACCCAAA**	**Gene mapping**
VrDf-SSR030	ACAAATTGCCTTCACTGTTT	TTTACAAAAGAAGATGTAAGTATTGA	Gene mapping
**VrDf-SSR031**	**GTTCTGGGCAGCAAGGAAT**	**TCTGCCATTTCCCATTTCTC**	**Gene mapping**
**VrDf-SSR032**	**TTCCGGATTGTAATAATCTCTCC**	**TTGTGTGTCTGCTGGGATGT**	**Gene mapping**
**VrDf-SSR033**	**TTGTTGATTCCGATAATAACTTGG**	**AATAATCACACGGTTATCTTCTAAACA**	**Gene mapping**
**VrDf-SSR034**	**CCGATAAAAACATTTTCTTAAGGTTC**	**AAAGATAAAAGGTCGTGATAATTGG**	**Gene mapping**
**VrDf-SSR035**	**GATGGCTTTAATTCCACACACA**	**CCACACATATATGCATTCGTACTG**	**Gene mapping**
**VrDf-SSR036**	**GCTAATATCAGCAGGAAATTCATACA**	**TTCGTGGTTGTTTTATGTTAGAGG**	**Gene mapping**
VrDf-SSR037	TGAGATAGTATGCAGTTTTTGTC	CAACACTTCAAGGCCTACAT	Gene mapping
VrDf-SSR038	TGAGATAGTATGCAGTTTTTGTC	CAACACTTCAAGGCCTACAT	Gene mapping
**VrDf-SSR039**	**AGGCCCATCTTTGTGAAATG**	**TGGAATTCCTGTGTCTCAAGC**	**Gene mapping**
**VrDf-SSR040**	**CTTTTGGGCCAATACATCGT**	**TCTGATGGTTTGGAGCAGTG**	**Gene mapping**
**VrDf-SSR041**	**GGTCCATTGTAGGCCAATGT**	**TGTATTTTTAAAAGTTCCCATGACA**	**Gene mapping**
**VrDf-SSR042**	**TCGATGTGATACTCCACATTTCTC**	**CCACAACTAACACAAAGCCAAT**	**Gene mapping**
**VrDf-SSR043**	**TTAGCCACTTGCGTCTTGTG**	**ATGGTCGCTAGGTTCACCAC**	**Gene mapping**
VrDf-SSR044	GTTTGTTCTGAAAGATGCAA	TTGGGAGGAGATTTTTCTTT	Gene mapping
**VrDf-SSR045**	**TGTCACGCATTCTCTTTTTCA**	**CCCACGCACATTTCTATTCC**	**Gene mapping**
**VrDf-SSR046**	**AGCCTAATATGCACGGTTGG**	**CATGCAGTCATTGACCTTGTG**	**Gene mapping**
**VrDf-SSR047**	**GCAGGCAAATGCAAATGTAA**	**GCCAAGTGCAAGAAAACTCC**	**Gene mapping**
VrE1-SSR1	AGCAAAACCGGGTTAGGATT	AATTGCCATCACCAGAGAGG	Gene mapping
**VrE1-SSR2**	**ATGGCAACTGCTCATGTCAA**	**CCTGGAGGAAGGGTCATACA**	Gene mapping
VrE1-SSR3	ACCAGCCAAAATCTAGTGCCA	AGGTTACATTTTGGTCCCACGA	Gene mapping
VrE1-SSR4	ACAACACAAACAACATGCATCCT	GCGCGTGGTAAGTCAAGTTG	Gene mapping
**VrE1-SSR5**	**GGCTTTGGACGAGTGACAGA**	**CGGCAATGTACATGTGTAGAGG**	**Gene mapping**
VrE1-SSR6	CAGACGAAATTGGATATTGACA	TTTAGTGTGAGCGTGATTGA	Gene mapping
**VrE1-SSR7**	**AAACAGTGTGCTCCTCGTGG**	**TGTGTGTGCAGGACGATGAT**	**Gene mapping**
VrE1-SSR8	CGAACTGATACGTTATGCATG	TCCTAGTCAACTTGATCAGAAA	Gene mapping
VrE1-SSR9	GTGAGAGTGGATTTGGAAGT	GGTTTCATCCCTTGTATTCAC	Gene mapping

**VrE1-SSR10**	**AGGATTTTCGTTGTTACCGA**	**GCAACTATGAGGAAACAAGC**	**Gene mapping**
**VrE1-SSR11**	**TCACTCTTCAACACTTAACCA**	**CCGTAATCATAACAAAACTCAGT**	**Gene mapping**
**VrE1-SSR12**	**GTACACAACCGACCAATGTA**	**AGATGATGCGAGATAAACCTT**	**Gene mapping**
VrE1-SSR13	TTTGTCAATCGTCCTGAAGT	TATTATACTTGCACGGGGTC	Gene mapping
VrE1-SSR14	CAGCAACACATTCACAAGTT	GGTTTGCGGAAGAATTAGTG	Gene mapping
VrE1-SSR16	AGATTCGTACGTGTGTTTGA	AGTGATGAATTATAGAATAGAAAGCA	Gene mapping
VrE1-SSR17	TCATTATAAAGACAAGAAATTTCAATG	CATGAACTGCAATAAGAGAAGT	Gene mapping
**VrE1-SSR18**	**AGTCACTGTTCACCTTTCAA**	**ACACTCAACACCAAATAATACC**	**Gene mapping**
VrE1-SSR19	GTTGAACTAACGGTGTTGAC	AATAATGCTTACGGTGGGTT	Gene mapping
**VrE1-SSR21**	**AGGTGCAGATTTATGTGAGG**	**ACCTTCTCTTGCTTGCTAAA**	**Gene mapping**
**VrE1-SSR23**	**GGGCTTTTACTCTCTTTTGC**	**TCCAGGTTTAATGATACTGTGG**	**Gene mapping**
VrE1-SSR24	TGACAGACACAGTAGGATCA	TCTTACCTAGAGTCCCAGTG	Gene mapping
**VrE1-SSR25**	**ACGTGATTAAGTTTTCGTGAA**	**TTTTACCCGAAACTCAACCC**	**Gene mapping**
**VrE1-SSR26**	**ACCGCCACTTTTAATGATTG**	**AACAACGTGAATAAACTGGC**	**Gene mapping**
VrE1-SSR27	GCTCAACATCATCTTTTCACA	AAGCGTTCAAAAGAAACTCC	Gene mapping
**VrE1-SSR28**	**TGCTAATCCCGATAACAACC**	**ACCGTTGGACAGTTAATAGT**	**Gene mapping**
**VrE1-SSR29**	**GGGCTTTTACTCTCTTTTGC**	**TCCAGGTTTAATGATACTGTGG**	**Gene mapping**
**VrE1-SSR30**	**TAAAGGCTTCGATGAACACA**	**ACGATAAAGAATATGTAGCAGAT**	**Gene mapping**
VrE1-SSR31	TGTCAGTGTGAGTAAATGTGA	TTTTGTCTACTGAAACCCGG	Gene mapping
**VrE1-SSR32**	**CTATTTTATCAATTTTATAACTCTTTCTGT**	**ATGCAGCACAGATATGGATT**	**Gene mapping**
VrE1-SSR33	TCCACATCCAGTTTTCATCT	TTCGTCACTTGTGCATAGTT	Gene mapping
VrE1-SSR34	AACTATGGATGGATGACGTC	AACCAAAATCGTTCCAAAGT	Gene mapping
**VrE1-SSR35**	**ACCAAAATCGTCCCAAAGTA**	**GAGTTGAATCCAGAAATAAGTCA**	**Gene mapping**
**VrE1-SSR36**	**TTATTCTGTCGTGGCATAGG**	**ACCAGATACTAAAACCAAAACT**	**Gene mapping**
**VrE1-SSR37**	**TCCACACTGTGTTTTCTGAT**	**TCAATCTAAGATTTTCTGTAGCA**	**Gene mapping**
VrE1-SSR39	GCTGCAGAACATGTTAAAGA	GCAAACAAGCACAAGGAAG	Gene mapping
**VrE1-SSR40**	**TGTCATGAAAATACAGAGAACG**	**ACCGTTGGACAGTTAATAGT**	**Gene mapping**
VR03-ID001	GCATGAGATTGTGCGCTTAG	TCCTAGCCATTTAAACGTACAC	Gene mapping
VR03-ID002	ATCCAAAAGACGCCGCTTTT	TGCGGCATTACATTCCAGGT	Gene mapping
VR03-ID003	GTTGGTCCATCTGAGTCGGC	GCCATGGACTCCTGAGGAAG	Gene mapping

VR03-ID004	CGACGCCCTGGACAACTATC	ATCAGAAACTGCAATGCCGC	Gene mapping
VR03-ID005	TCCTCACTCGCACACTTTCC	ACGACTCGAACATACACCGG	Gene mapping
VR03-ID006	GGAGTTTGCGGATTCCATTCC	TCCTACGATGTCCATACCCCA	Gene mapping
VR03-ID007	ATCGTTGAAGCTAGCAGTGG	TCTGAGCCTTAGGATTTCTTGC	Gene mapping
**VR03-ID008**	**ACTTGGAAATGATGTGTCTTGA**	**TGGCTTCCGTGTTTTCAAATGT**	**Gene mapping**
VR03-ID009	TAGGGCTTGATTGGGCTTGG	AAAGTGCAGACAGAGGAAAC	Gene mapping
VR03-ID010	TCACTGTTATTGGTCCGCGT	AGTTGGGTATGACAGGAGAACC	Gene mapping
VR03-ID011	CCCAACAGCTGAAACATGCC	TGGGCCACGAACAGAATGAA	Gene mapping
VR03-ID012	GGGCTGCCACGAAGACTTTA	CCCTGATGGTCCCTATTTTCGT	Gene mapping
**VR03-ID013**	**AAAGGCAGTCACATCAACCA**	**CACATCGATGGCATCTTACA**	**Gene mapping**
VR03-ID014	TTTGAGCAGCCAGAAAGTTT	TGCGTTATAAGGGCGTGGTT	Gene mapping
VR03-ID015	GACCCGACTCCGCTCAAC	CAATGGCAGCTATGGAGGGT	Gene mapping
VR03-ID016	TGATTTTGGACAAGACGAAACA	TGGGCATCCTTCACTCAATCT	Gene mapping
VR03-ID017	GGGGCTCTAACATCACACCC	TGTAACTCGTTGGGCAAGCC	Gene mapping
**VR03-ID018**	**CCTCAAACCCAAACACCCCT**	**CGGGGAATGTCTTTGGCTTG**	**Gene mapping**
VR03-ID019	GCCCTTCGGTTTGGCAAATA	CCCCAACACTGCCAAAACC	Gene mapping
VR03-ID020	AGGTCTTCAAGCACAGTCAGT	CCTGGCTAGAAACATACTCCCC	Gene mapping
VR03-ID021	GTCCTCGAACCACACCGTAC	ACTCCATGGTTAAGCGTGCT	Gene mapping
VR03-ID022	AGGAAGACTTGTTCATCTCATG	TCTCGTCCCACCACTCTCTT	Gene mapping
Vr03-Indel23	CCACCTGCAACCAACAACAG	GCCCATTGCCCTCCATAAAG	Gene mapping
Vr03-Indel24	GGTGGCATGTGGAGGCAC	CAGTCTCCGGTACCCATTGC	Gene mapping
**Vr03-Indel25**	**TGTCCAGCCTTTACTTCAGCA**	**GGGAGGAGACAGCAGGTTTG**	**Gene mapping**
**Vr03-Indel26**	**ACTGACACTTCAAAACCTATCA**	**CGGTTTCATTCATTAGCTCTGC**	**Gene mapping**
Vr03-Indel27	GGAAGACTATGAAATGTGGGCA	TCTGGTGAAGATCAATTTCGT	Gene mapping
VR05-ID001	TTATCGTTGGCCATGGGAGG	TTTATCTTTGTTGCCGCCGG	Gene mapping
**VR05-ID002**	**ACCTCAGACGCTCCTCTTTC**	**CCCAAGTCCAGGCCCAATAT**	**Gene mapping**
VR05-ID003	GGGTGCAAGACTCGCCATAG	GGCCTCATTCTCCTCTGCTC	Gene mapping
**VR05-ID004**	**TGAGAAAGTTGGTATGCCCTCT**	**AAAGGGACACTCGGAGGTCT**	**Gene mapping**
**VR05-ID005**	**ACAACTTGCCACGTGTCTGA**	**AGTGTGTTCAGAGGGAGAGA**	**Gene mapping**
VR05-ID006	TTGTGTGTGGTCGGGTGAAA	CAAATGCAAGGGTAAGCTAGGT	Gene mapping

VR05-ID007	TGCAGATGGATAAGGCTTCGA	TCAAGGACTTCAATTGGGGAGT	Gene mapping
VR05-ID008	TGATATTGTGTGGCGTGTCC	TTTAATTGAAGCGGGCCGAC	Gene mapping
**VR05-ID009**	**AGACCTAGCTTCCCTTACCTGA**	**CTTGGACCTAGGGCATGCAA**	**Gene mapping**
VR05-ID010	CCGGAATGAGAGGGATCTGG	TCCTCACTCGCACACTTTCC	Gene mapping
VR05-ID011	GCTGAGCTATATGTCTCTGGA	GAAACATCAGCGTCGTCACTG	Gene mapping
VR05-ID012	TGTGGGATGTGTGTTGAATGT	CATGGCACTCTGAAACGTGG	Gene mapping
VR05-ID013	GATTGACCTTGCTCCTACGT	AAGGTTTAAGCACGCAGGT	Gene mapping
VR05-ID014	ACCTCTGCACCTCTCTCATCA	TACACGTGTTGCTACGTCCC	Gene mapping
VR05-ID015	TGGCATTACTTGGTGACCGT	TCCGAGAACCCTGATAGTGGT	Gene mapping
VR05-ID016	TGGCAGGGATCCAATTCTTTCA	ACCAAACATCTCAGAAGCGTC	Gene mapping
VR05-ID017	AAACGGGTTGACTCACTAGC	CCGCCAACCCACTTACCTAA	Gene mapping
VR05-ID018	TGGTTTGCTGCAAGTGTGAA	AAGCCACCATGTTGTCGTCA	Gene mapping
**VR05-ID019**	**TGGTAGTGGAAGAAGGTTGGG**	**TTTCACACACCAACTCCGCA**	**Gene mapping**
VR05-ID020	CCAAAACAGTGGCATGAAAA	TTAGTTTCACCCATGTCTTTCA	Gene mapping
VR05-ID021	TGGAAATGAGGCTGAGTGGT	GGTTTTGTCAGAGGATCCGC	Gene mapping
VR05-ID022	ACCCTACGCATTTCTGGTTCT	CTCCTAGGGTCAAGCACAACA	Gene mapping
VR05-ID023	CCAGCAAGGCATGTCAATCT	GCTTGGAAGATGCACATACA	Gene mapping
VR05-ID024	ACTTTCCAAGGCATGACTTT	TGCGGAGGTGTAGGGTGT	Gene mapping
VR05-ID025	ACCGCGGCATATACTTTCTCA	ACCGCGGTATATAAGTTCCG	Gene mapping
**VR05-ID026**	**TCCGATCCTCCTTACATCCCA**	**CCTGAGTTGGCATATCCCTGA**	**Gene mapping**
**Vr05-Indel027**	**AAGCACAACCTGAGGAAAGG**	**ACAGAACCAAAATCCCAGCA**	**Gene mapping**
Vr05-Indel028	AGCTATGGATGTTTTGTGCT	GGTGATGAACTTGCTGAAAT	Gene mapping
Vr05-Indel029	TGGCAAATTATTGGCTTTGG	CTTGGGTTGGCAAGTCTCAC	Gene mapping
Vr05-Indel030	CAGGCACATGATCCTCAGAA	CAACCCTTACATTTGCACGTA	Gene mapping
**Vr05-Indel031**	**GGGTTGGATTTTCCTCTTTTT**	**GAGGAGGGTAAAAATTCACGA**	**Gene mapping**
Vr05-Indel032	CAAATACGGCCATGTTAGGG	TCGAGGTTCCTATGTTTTACCAA	Gene mapping
Vr05-Indel033	CCACTATGTTTTGAAGCATTT	CCATTTGGTTTGTACTTATGC	Gene mapping
**Vr05-Indel034**	**ACAAGCATACCCTCCAACCA**	**GTCCTGCAATTGCCTGCT**	**Gene mapping**
**Vr05-Indel035**	**GGGAAAAGCCAAATTCCTTG**	**CTTCTCAGACCCCCTCACC**	**Gene mapping**
Vr05-Indel036	GGGGGTTTCGGTTATGATTT	ACCAAATTGAAAAACCCCTTT	Gene mapping

M0768	GTCAAGGAAGGTCAAACCCA	ACAGAGAGACCGGAAGCGTA	Gene mapping
SeqPhyE1	AAAAGCTGGAATCAAACTG	GAAAGAAACAAATCGTGGT	*VrPhyE* gene sequencing
SeqPhyE2	TGCAAAGCCAGTTAAGGTC	TTCATCCATCGCTCCAGT	*VrPhyE* gene sequencing
SeqPhyE3	GATAGGGATGATGGAGGAA	TCTGCCTCTTGGCTTGTG	*VrPhyE* gene sequencing
SeqPhyE4	TTTGATTCGTTCTCCCTG	GTATAATCGGAGGACGAAA	*VrPhyE* gene sequencing
SeqPhyE5	ATGGCATACGATTCACGC	TGGAGGAAGGGTCATACAAT	*VrPhyE* gene sequencing
qPhyE	GAACAGCAGGAGGGACAAGA	CCCTTTGGATTTTGGACAGA	*VrPhyE* gene expression analysis

Supplemental Table 2 Marker genotypes and days to first flowering of F_2 plants of the cross Kamphaeng Saen 2 (KS2) × ACC41 used for QTL analysis. A, B and H present genotype of Kamphaeng Saen 2 (KS2), ACC41 and hybrid (heterozygous), respectively. M1, M2, M3, M4, M5, M6 M7, M8, M9, M10, M11, M12, M13, M14 and M15 represent markers VrE1-SSR5, VrE1-SSR10, VrE1-SSR11, VrE1-SSR2, Vr05-ID19, VrE1-SSR21, VrDf-SSR5, VrDf-SSR47, VrDf-SSR45, Vr03-ID26, VrDf-SSR43, Vr05-ID31, VrDf-SSR39, VrDf-SSR17 and VrDf-SSR19, respectively.

F_2 individual	Marker															Days to first flowering
	M1	M2	M3	M4	M5	M6	M7	M8	M9	M10	M11	M12	M13	M14	M15	
1	B	B	B	B	B	B	B	B	B	B	B	B	B	B	B	42
2	H	H	H	H	H	H	H	H	H	H	H	H	H	H	H	55
3	H	H	H	H	H	H	H	H	H	H	H	H	H	H	H	39
4	H	H	H	H	H	H	H	H	H	H	H	H	H	H	H	70
5	B	B	B	B	B	B	B	B	B	B	B	B	B	B	B	52
6	H	H	H	H	H	H	H	H	H	H	H	H	H	H	H	46
7	H	H	H	H	H	H	H	H	H	H	H	H	H	H	H	50
8	H	H	H	H	H	H	H	H	H	H	H	H	H	H	H	44
9	A	A	A	A	A	A	A	A	A	A	A	A	A	A	A	44
10	H	H	H	H	H	H	H	H	H	H	H	H	H	H	H	42
11	H	H	H	H	H	H	H	H	H	H	H	H	H	H	H	44
12	A	A	A	A	A	A	A	A	A	A	A	A	A	A	A	44
13	B	B	B	B	B	B	B	B	B	B	B	B	B	B	B	61
14	A	A	A	A	A	A	A	A	A	A	A	A	A	A	A	41
15	H	H	H	H	H	H	H	H	H	H	H	H	H	H	H	47
16	H	H	H	H	H	H	H	H	H	H	H	H	H	H	H	52
17	H	H	H	H	H	H	H	H	H	H	H	H	H	H	H	39
18	H	H	H	H	H	H	H	H	H	H	H	H	H	H	H	43
19	H	H	H	H	H	H	H	H	H	H	H	H	H	H	H	49
20	H	H	H	H	H	H	A	A	A	A	A	A	A	A	A	52
21	H	H	H	H	H	H	H	H	H	H	H	H	H	H	H	44
22	B	B	B	B	B	B	B	B	B	B	B	B	B	B	B	44
23	B	B	B	B	B	B	B	B	B	B	B	B	B	B	B	53
24	H	H	H	H	H	H	H	H	H	H	H	H	H	H	H	47
25	B	B	B	B	B	B	B	B	B	B	B	B	B	B	B	47
26	A	A	A	A	A	A	A	A	A	A	A	A	A	A	A	33
27	A	A	A	A	A	A	A	A	A	A	A	A	A	A	A	36
28	H	H	H	H	H	H	H	H	H	H	H	H	H	H	H	53
29	H	H	H	H	H	H	H	H	H	H	H	H	H	H	H	47
30	B	B	B	B	B	B	B	B	B	B	B	B	B	B	B	54
31	H	H	H	H	H	H	H	H	H	H	H	H	H	H	H	51
32	H	H	H	H	H	H	H	H	H	H	H	H	H	H	H	49
33	A	A	A	A	A	A	A	A	A	A	A	A	A	A	A	44
34	A	A	A	A	A	A	A	A	A	A	A	A	A	A	A	48
35	H	H	H	H	H	H	H	H	H	H	H	H	H	H	H	44
36	B	B	B	B	B	B	B	B	B	B	B	B	B	B	B	51
37	B	B	B	B	B	B	B	B	B	B	B	B	B	B	B	53
38	H	H	H	H	H	H	H	H	H	H	H	H	H	H	H	43
39	A	A	A	A	A	A	A	A	A	A	A	A	A	A	A	37
40	B	B	B	B	B	B	B	B	B	B	B	B	B	B	B	45
41	B	B	B	B	B	B	B	B	B	B	B	B	B	B	B	46
42	H	B	B	B	B	B	B	B	B	B	B	B	B	B	B	62

43	H	H	H	H	H	H	H	H	H	H	H	H	H	H	H	42
44	H	B	B	B	B	B	B	B	B	B	B	B	B	B	B	49
45	A	A	A	A	A	A	A	A	A	A	A	A	A	A	A	32
46	A	A	A	A	A	A	A	A	A	A	A	A	A	A	A	46
47	H	H	H	H	H	H	H	H	H	H	H	H	H	H	H	44
48	B	B	B	B	B	B	B	B	B	B	B	B	B	B	B	55
49	B	B	B	B	B	B	B	B	B	B	B	B	B	B	B	51
50	A	A	A	A	A	A	A	A	A	A	A	A	A	A	A	41
51	H	H	H	H	H	H	H	H	H	H	H	H	H	H	H	37
52	H	B	B	B	B	B	B	B	B	B	B	B	B	B	B	45
53	H	H	H	H	H	H	H	H	H	H	H	H	H	H	H	43
54	H	H	H	H	H	H	H	H	H	H	H	H	H	H	H	40
55	H	H	H	H	H	H	H	H	H	H	H	H	H	H	H	46
56	H	H	H	H	H	H	H	H	H	H	H	H	H	H	H	49
57	H	H	H	H	H	H	H	H	H	H	H	H	H	H	H	56
58	H	H	H	H	H	H	H	H	H	H	H	H	H	H	H	43
59	H	H	H	H	H	H	H	H	H	H	H	H	H	H	H	47
60	H	H	H	H	H	H	H	H	H	H	H	H	H	H	H	46
61	A	A	A	A	A	A	A	A	A	A	A	A	A	A	A	42
62	H	H	H	H	H	H	H	H	H	H	H	H	H	H	H	46
63	A	A	A	A	A	A	A	A	A	A	A	A	A	A	A	43
64	H	H	H	H	H	H	H	H	H	H	H	H	H	H	H	55
65	H	H	H	H	H	H	H	H	H	H	H	H	H	H	H	53
66	H	H	H	H	H	H	H	H	H	H	H	H	H	H	H	68
67	H	H	H	H	H	H	H	H	H	H	H	H	H	H	H	42
68	B	B	B	B	B	B	B	B	B	B	B	B	B	B	B	56
69	B	B	B	B	B	B	B	B	B	B	B	B	B	B	B	57
70	H	H	H	H	H	H	H	H	H	H	H	H	H	H	H	58
71	H	H	H	H	H	H	H	H	H	H	H	H	H	H	H	52
72	B	B	B	B	B	B	B	B	B	B	B	B	B	B	B	63
73	H	H	H	H	H	H	H	H	H	H	H	H	H	H	H	41
74	A	A	A	A	A	A	A	A	A	A	A	A	A	A	A	37
75	B	B	B	B	B	B	B	B	B	B	B	B	B	B	B	47
76	H	H	H	H	H	H	H	H	H	H	H	H	H	H	H	56
77	H	H	H	H	H	H	H	H	H	H	H	H	H	H	H	39
78	B	B	B	B	B	B	B	B	B	B	B	B	B	B	B	57
79	A	A	A	A	A	A	A	A	A	A	A	A	A	A	A	46
80	H	H	H	H	H	H	A	A	A	A	A	A	A	A	A	47
81	H	H	H	H	H	H	H	H	H	H	H	H	H	H	H	70
82	H	H	H	H	H	H	H	H	H	H	H	H	H	H	H	44
83	B	B	B	B	B	B	B	B	B	B	B	B	B	B	B	46
84	B	B	B	B	B	B	B	B	B	B	B	B	B	B	B	47
85	H	H	H	H	H	H	B	B	B	B	B	B	B	B	B	39
86	A	A	A	A	A	A	A	A	A	A	A	A	A	A	A	50
87	H	H	H	H	H	H	H	H	H	H	H	H	H	H	H	47
88	H	H	H	H	H	H	H	H	H	H	H	H	H	H	H	45
89	B	B	B	B	B	B	B	B	B	B	B	B	B	B	B	44
90	B	B	B	B	B	B	B	H	H	H	H	H	H	H	H	51
91	A	A	A	A	A	A	A	A	A	A	A	A	A	A	A	56

92	B	B	B	B	B	B	B	B	B	B	B	B	B	B	B	41
93	B	B	B	B	B	B	B	H	H	H	H	H	H	H	H	42
94	H	H	H	H	H	H	H	H	H	H	H	H	H	H	H	52
95	H	B	B	B	B	B	B	B	B	B	B	B	B	B	B	48
96	A	A	A	A	A	A	A	A	A	A	A	A	A	A	A	41
97	A	A	A	A	A	A	H	H	H	H	H	H	H	H	H	43
98	H	H	H	H	H	H	H	H	H	H	H	H	H	H	H	47
99	H	H	H	H	H	H	H	H	H	H	H	H	H	H	H	50
100	H	H	H	H	H	H	H	H	H	H	H	H	H	H	H	49
101	H	H	H	H	H	H	H	H	H	H	H	H	H	H	H	62
102	H	H	H	H	H	H	H	H	H	H	H	H	H	H	H	45
103	B	B	B	B	B	B	H	H	H	H	H	H	H	H	H	42
104	H	H	H	H	H	H	H	H	H	H	H	H	H	H	H	40
105	A	A	A	A	A	A	A	A	A	A	A	A	A	A	A	36
106	A	A	A	A	A	A	A	A	A	A	A	A	A	A	A	41
107	B	B	B	B	B	B	B	B	B	B	B	B	B	B	B	50
108	H	H	H	H	H	H	H	H	H	H	H	H	H	H	H	48
109	H	B	B	B	B	B	B	B	B	B	B	B	B	B	B	44
110	H	H	H	H	H	H	A	A	A	A	A	A	A	A	A	44
111	A	A	A	A	A	A	H	H	H	H	H	H	H	A	A	39
112	B	B	B	B	B	B	B	B	B	B	B	B	B	B	B	62
113	H	H	H	H	H	H	H	H	H	H	H	H	H	H	H	56
114	A	A	A	A	A	A	A	A	A	A	A	A	A	A	A	42
115	A	A	A	A	A	A	A	A	A	A	A	A	A	A	A	43
116	H	H	H	H	H	H	H	H	H	H	H	H	H	H	H	56
117	B	B	B	B	B	B	B	B	B	B	B	B	B	B	B	41
118	H	H	H	H	H	H	H	H	H	H	H	H	H	H	H	43
119	H	H	H	H	H	H	H	H	H	H	H	H	H	H	H	48
120	A	A	A	A	A	A	A	A	A	A	A	A	A	A	A	45
121	A	A	A	A	A	A	A	A	A	A	A	A	A	A	A	41
122	A	A	A	A	A	A	A	A	A	A	A	A	A	A	A	41
123	A	A	A	A	A	A	A	A	A	A	A	A	A	A	A	40
124	H	H	H	H	H	H	H	H	H	H	H	H	H	H	H	44
125	H	B	B	B	B	B	B	B	B	B	B	B	B	B	B	56
126	H	B	B	B	B	B	B	B	B	B	B	B	B	B	B	63
127	A	A	A	A	A	A	A	A	A	A	A	A	A	A	A	56
128	B	B	B	B	B	B	B	B	B	B	B	B	B	B	B	57
129	H	H	H	H	H	H	H	H	H	H	H	H	H	H	H	57
130	H	B	B	B	B	B	B	B	B	B	B	B	B	B	B	61
131	H	B	B	B	B	B	B	B	B	B	B	B	B	B	B	56
132	H	H	H	H	H	H	H	H	H	H	H	H	H	H	H	55
133	H	H	H	H	H	H	H	H	H	H	H	H	H	H	H	46
134	B	B	B	B	B	B	B	B	B	B	B	B	B	B	B	45
135	H	H	H	H	H	H	H	H	H	H	H	H	H	H	H	55
136	H	H	H	H	H	H	H	H	H	H	H	H	H	H	H	46
137	B	B	B	B	B	B	B	B	B	B	B	B	B	B	B	40
138	A	A	A	A	A	A	A	A	A	A	A	A	A	A	A	41
139	B	H	H	H	H	H	H	H	H	H	H	H	H	H	H	56
140	H	H	H	H	H	H	H	H	H	H	H	H	H	H	H	47

141	H	H	H	H	H	H	H	H	H	H	H	H	H	H	H	47
142	H	H	H	H	H	H	H	H	H	H	H	H	H	H	H	50
143	H	H	H	H	H	H	H	H	H	H	H	H	H	H	H	46
144	H	H	H	H	H	H	H	H	H	H	H	H	H	H	H	55
145	B	H	H	H	H	H	H	H	H	H	H	H	H	H	H	46
146	B	H	H	H	H	H	H	H	H	H	H	H	H	H	H	50
147	A	A	A	A	A	A	A	A	A	A	A	A	A	A	A	41
148	H	B	B	B	B	B	B	B	B	B	B	B	B	B	B	48
149	A	A	A	A	A	A	A	A	A	A	A	A	A	A	A	38
150	A	A	A	A	A	A	A	A	A	A	A	A	A	A	A	44
151	H	H	H	H	H	H	H	H	H	H	H	H	H	H	H	42
152	H	H	H	H	H	H	H	H	H	H	H	H	H	H	H	44
153	H	H	H	H	H	H	H	H	H	H	H	H	H	H	H	40
154	H	B	B	B	B	B	B	B	B	B	B	B	B	B	B	45
155	H	H	H	H	H	H	H	H	H	H	H	H	H	H	H	49
156	A	A	A	A	A	A	A	A	A	A	A	A	A	A	A	44
157	H	H	H	H	H	H	H	H	H	H	H	H	H	H	H	49
158	H	H	H	H	H	H	H	H	H	H	H	H	H	H	H	46
159	B	B	B	B	B	B	B	B	B	B	B	B	B	B	B	43
160	A	A	A	A	A	A	A	A	A	A	A	A	A	A	A	43
161	H	H	H	H	H	H	H	H	H	H	H	H	H	H	H	55
162	H	H	H	H	H	H	H	H	H	H	H	H	H	H	H	42
163	H	H	H	H	H	H	H	H	H	H	H	H	H	H	H	48
164	A	A	A	A	A	A	A	A	A	A	A	A	A	A	A	40
165	H	H	H	H	H	H	H	H	H	H	H	H	H	H	H	57
166	H	H	H	H	H	H	B	B	B	B	B	B	B	B	B	50
167	B	B	B	B	B	B	B	B	B	B	B	B	B	B	B	54
168	A	A	A	A	A	A	A	A	A	A	A	A	A	A	A	48
169	A	A	A	A	A	A	A	A	A	A	A	A	A	A	A	41
170	A	A	A	A	A	A	A	A	A	A	A	A	A	A	A	43
171	A	A	A	A	A	A	A	A	A	A	A	A	A	A	A	44
172	H	H	H	H	H	H	H	H	H	H	H	H	H	H	H	39
173	H	H	H	H	H	H	H	H	H	H	H	H	H	H	H	46
174	H	H	H	H	H	H	H	H	H	H	H	H	H	H	H	42
175	H	H	H	H	H	H	H	H	H	H	H	H	H	H	H	42
176	B	B	B	B	B	B	B	B	B	B	B	B	B	B	B	40
177	H	B	B	B	B	B	B	B	B	B	B	B	B	B	B	63
178	H	H	H	H	H	H	H	H	H	H	H	H	H	H	H	57
179	H	B	B	B	B	B	B	B	B	B	B	B	B	B	H	45
180	H	H	H	H	H	H	A	A	A	A	A	A	A	A	A	48
181	H	H	H	H	H	H	B	B	B	B	B	B	B	B	H	44
182	H	H	H	H	H	H	B	B	B	B	B	B	B	B	B	41
183	H	H	H	H	H	H	H	H	H	H	H	H	H	H	H	44
184	H	H	H	H	H	H	A	A	A	A	A	A	A	A	A	37
185	A	A	A	A	A	A	A	A	A	A	A	A	A	A	A	46
186	H	H	H	H	H	H	H	H	H	H	H	H	H	H	H	53
187	H	H	H	H	H	H	H	H	H	H	H	H	H	H	H	42
188	H	H	H	H	H	H	H	H	H	H	H	H	H	H	H	47
189	B	H	H	H	H	H	H	H	H	H	H	H	H	H	H	45

190	A	A	A	A	A	A	H	H	H	H	H	H	H	H	H	44
191	H	B	B	B	B	B	H	H	H	H	H	H	H	H	H	51
192	H	H	H	H	H	H	H	H	H	H	H	H	H	H	H	68
193	H	H	H	H	H	H	H	H	H	H	H	H	H	H	H	46
194	H	H	H	H	H	H	H	H	H	H	H	H	H	H	H	44
195	A	A	A	A	A	A	A	A	A	A	A	A	A	A	A	55
196	H	H	H	H	H	H	H	H	H	H	H	H	H	H	H	46
197	H	B	B	B	B	B	B	B	B	B	B	B	B	B	B	45
198	A	A	A	A	A	A	A	A	A	A	A	A	A	A	A	39
199	B	B	B	B	B	B	B	B	B	B	B	B	B	B	B	45
200	H	H	H	H	H	H	H	H	H	H	H	H	H	H	H	37
201	H	H	H	H	B	B	B	B	B	B	B	B	B	B	B	55
202	H	H	H	H	H	H	H	H	H	H	H	H	H	H	H	53
203	H	H	H	H	H	H	H	H	H	H	H	H	H	H	H	41
204	H	H	H	H	H	H	B	B	B	B	B	B	B	B	B	45
205	H	B	B	B	B	B	B	B	B	B	B	B	B	B	B	65
206	H	H	H	H	H	H	H	H	H	H	H	H	H	H	H	52
207	A	A	A	A	A	A	A	A	A	A	A	A	A	A	A	41
208	H	H	H	H	H	H	H	H	H	H	H	H	H	H	H	44
209	B	B	B	B	B	B	B	B	B	B	B	B	B	B	B	61
210	B	B	B	B	B	B	B	B	B	B	B	B	B	B	B	50
211	H	H	H	H	H	H	H	H	H	H	H	H	H	H	H	43
212	H	H	H	H	H	H	H	H	H	H	H	H	H	H	H	43
213	A	A	A	A	A	A	A	A	A	A	A	A	A	A	A	41
214	H	H	H	H	H	H	H	H	H	H	H	H	H	H	H	41
215	A	A	A	A	A	A	A	A	A	A	A	A	A	A	A	46
216	H	B	B	B	B	B	H	H	H	H	H	H	H	H	H	80
217	H	H	H	H	H	H	B	B	B	B	B	B	B	B	B	43
218	H	H	H	H	H	H	H	H	H	H	H	H	H	H	H	48
219	B	B	B	B	B	B	A	A	A	A	A	A	A	A	A	43
220	H	H	H	H	H	H	H	H	H	H	H	H	H	H	H	43
221	H	H	H	H	H	H	H	H	H	H	H	H	H	H	H	42
222	H	B	B	B	B	B	B	B	B	B	B	B	B	B	B	55
223	H	B	B	B	B	B	B	B	B	B	B	B	B	B	B	47
224	H	H	H	H	H	H	H	H	H	H	H	H	H	H	H	46
225	H	H	H	H	H	H	B	B	B	B	B	B	B	B	B	50
226	H	H	H	H	H	H	H	H	H	H	H	H	H	H	H	46
227	A	A	A	A	A	A	A	A	A	A	A	A	A	A	A	40
228	A	A	A	A	A	A	A	A	A	A	A	A	A	A	A	42
229	H	H	H	H	H	H	H	H	H	H	H	H	H	H	H	48
230	H	B	B	B	B	B	B	B	B	B	B	B	B	B	B	46
231	H	B	B	B	B	B	B	B	B	B	B	B	B	B	B	41
232	H	B	B	B	B	B	B	B	B	B	B	B	B	B	B	50
233	H	B	B	B	B	B	B	B	B	B	B	B	B	B	B	41
234	H	H	H	H	H	H	H	H	H	H	H	H	H	H	H	44
235	B	B	B	B	B	B	B	B	B	B	B	B	B	B	B	52
236	H	B	B	B	B	B	B	B	B	B	B	B	B	B	B	64
237	H	B	B	B	B	B	B	B	B	B	B	B	B	B	B	51
238	H	H	H	H	H	H	H	H	H	H	H	H	H	H	H	45

239	H	H	H	H	H	H	H	H	H	H	H	H	H	H	H	41
240	B	B	B	B	B	B	H	H	H	H	H	H	H	H	H	53
241	H	B	B	B	B	B	A	A	A	A	A	A	A	A	A	51
242	H	B	B	B	B	B	B	B	B	B	B	B	B	B	B	55
243	H	H	H	H	H	H	A	A	A	A	A	A	A	A	A	45
244	H	H	H	H	H	H	H	H	H	H	H	H	H	H	H	40
245	H	B	B	B	B	B	B	B	B	B	B	B	B	B	B	42
246	H	H	H	H	H	H	H	H	H	H	H	H	H	H	H	49
247	A	A	A	A	A	A	A	A	A	A	A	A	A	A	A	36
248	H	H	H	H	H	H	H	H	H	H	H	H	H	H	H	39
249	H	H	H	H	H	H	H	H	H	H	H	H	H	H	H	55
250	B	B	B	B	B	B	H	H	H	H	H	H	H	H	H	46
251	A	A	A	A	A	A	A	A	A	A	A	A	A	A	A	39
252	H	H	H	H	H	H	H	H	H	H	H	H	H	H	H	42
253	H	H	H	H	H	H	H	H	H	H	H	H	H	H	H	40
254	H	H	H	H	H	H	H	H	H	H	H	H	H	H	H	41
255	H	H	H	H	H	H	H	H	H	H	H	H	H	H	H	46
256	H	H	H	H	H	H	H	H	H	H	H	H	H	H	H	45
257	A	A	A	A	A	A	A	A	A	A	A	A	A	A	A	49
258	A	A	A	A	A	A	A	A	A	A	A	A	A	A	A	42
259	H	H	H	H	H	H	H	H	H	H	H	H	H	H	H	40
260	H	B	B	B	B	B	B	B	B	B	B	B	B	B	B	54
261	A	A	A	A	A	A	A	A	A	A	A	A	A	A	A	44
262	H	H	H	H	H	H	H	H	H	H	H	H	H	H	H	47
263	B	B	B	B	B	B	B	B	B	B	B	B	B	B	B	43
264	H	H	H	H	H	H	H	H	H	H	H	H	H	H	H	61
265	H	H	H	H	H	H	H	H	H	H	H	H	H	H	H	40
266	B	B	B	B	B	B	B	B	B	B	B	B	B	B	B	47
267	H	H	H	H	H	H	H	H	H	H	H	H	H	H	H	49
268	H	H	H	H	H	H	H	H	H	H	H	H	H	H	H	44
269	H	H	H	H	H	H	H	H	H	H	H	H	H	H	H	43
270	H	H	H	H	H	H	H	H	H	H	H	H	H	H	H	48
271	A	A	A	A	A	A	A	A	A	A	A	A	A	A	A	47
272	H	H	H	H	H	H	H	H	H	H	H	H	H	H	H	40
273	B	B	B	B	B	B	B	B	B	B	B	B	B	B	B	46
274	H	H	H	H	H	H	H	H	H	H	H	H	H	H	H	47
275	A	A	A	A	A	A	A	A	A	A	A	A	A	A	A	38
276	H	B	B	B	B	B	B	B	B	B	B	B	B	B	B	41
277	B	B	B	B	B	B	B	B	B	B	B	B	B	B	B	43
278	H	H	H	H	H	H	H	H	H	H	H	H	H	H	H	45
279	H	H	H	H	H	H	H	H	H	H	H	H	H	H	H	47
280	H	H	H	H	H	H	H	H	H	H	H	H	H	H	H	45
281	H	H	H	H	H	H	A	A	A	A	A	A	A	A	A	44
282	H	H	H	H	H	H	H	H	H	H	H	H	H	H	H	45
283	A	A	A	A	A	A	A	A	A	A	A	A	A	A	A	41
284	B	B	B	B	B	B	B	B	B	B	B	B	B	B	B	47
285	H	H	H	H	H	H	H	H	H	H	H	H	H	H	H	47
286	A	A	A	A	A	A	A	A	A	A	A	A	A	A	A	41
287	H	H	H	H	H	H	A	A	A	A	A	A	A	A	A	41

288	H	H	H	H	H	H	H	H	H	H	H	H	H	H	H	49
289	H	H	H	H	H	H	H	H	H	H	H	H	H	H	H	45
290	H	H	H	H	H	H	A	A	A	A	A	A	A	A	A	41
291	B	B	B	B	B	B	B	B	B	B	B	B	B	B	B	42
292	A	A	A	A	A	A	A	A	A	A	A	A	A	A	A	41
293	A	A	A	A	A	A	A	A	A	A	A	A	A	A	A	42
294	H	H	H	H	H	H	A	A	A	A	A	A	A	A	A	43
295	H	H	H	H	H	H	A	A	A	A	A	A	A	A	A	43
296	H	H	H	H	H	H	H	H	H	H	H	H	H	H	H	38
297	A	A	A	A	A	A	A	A	A	A	A	A	A	A	A	41
298	A	A	A	A	A	A	A	A	A	A	A	A	A	A	A	37
299	H	H	H	H	H	H	H	H	H	H	H	H	H	H	H	41
300	H	H	H	H	H	H	H	H	H	H	H	H	H	H	H	41
301	H	B	B	B	B	B	B	B	B	B	B	B	B	B	B	48
302	A	A	A	A	A	A	A	A	A	A	A	A	A	A	A	38
303	H	B	B	B	B	B	H	H	H	H	H	H	H	H	H	37
304	H	B	B	B	B	B	B	B	B	B	B	B	B	B	B	43
305	H	H	H	B	B	B	B	B	B	B	B	B	B	B	B	64
306	A	A	A	A	A	A	A	A	A	A	A	A	A	A	A	43
307	H	B	B	B	B	B	B	B	B	B	B	B	B	B	B	45
308	A	A	A	A	A	A	A	A	A	A	A	A	A	A	A	44
309	B	B	B	B	B	B	B	B	B	B	B	B	B	B	B	43
310	H	H	H	H	H	H	H	H	H	H	H	H	H	H	H	49
311	H	H	H	H	H	H	B	B	B	B	B	B	B	B	B	52
312	H	H	H	H	H	H	H	H	H	H	H	H	H	H	H	45
313	H	H	H	H	H	H	A	A	A	A	A	A	A	A	A	48
314	A	A	A	A	A	A	A	A	A	A	A	A	A	A	A	39
315	A	A	A	A	A	A	A	A	A	A	A	A	A	A	A	43
316	H	H	H	H	H	H	H	H	H	H	H	H	H	H	H	44
317	H	H	H	H	H	H	H	H	H	H	H	H	H	H	H	46
318	H	H	H	H	H	H	H	H	H	H	H	H	H	H	H	46
319	H	H	H	H	H	H	H	H	H	H	H	H	H	H	H	39
320	A	A	A	A	A	A	A	A	A	A	A	A	A	A	A	45
321	B	B	B	H	H	H	H	H	H	H	H	H	H	H	H	40
322	H	H	H	H	H	H	H	H	H	H	H	H	H	H	H	48
323	A	A	A	A	A	A	A	A	A	A	A	A	A	A	A	35
324	H	H	H	H	H	H	B	B	B	B	B	B	B	B	B	41
325	A	A	A	A	A	A	A	A	A	A	A	A	A	A	A	45
326	H	H	H	H	H	H	A	A	A	A	A	A	A	A	A	46
327	H	B	B	B	B	B	B	B	B	B	B	B	B	B	B	47
328	A	A	A	A	A	A	A	A	A	A	A	A	A	A	A	41
329	H	H	H	H	H	H	H	H	H	H	H	H	H	H	H	46
330	B	B	B	B	B	B	B	B	B	B	B	B	B	B	B	39
331	H	H	H	H	H	H	H	H	H	H	H	H	H	H	H	49
332	H	H	H	H	H	H	H	H	H	H	H	H	H	H	H	41
333	A	A	A	A	A	A	A	A	A	A	A	A	A	A	A	47
334	H	H	H	H	H	H	H	H	H	H	H	H	H	H	H	55
335	H	H	H	H	H	H	H	H	H	H	H	H	H	H	H	41
336	B	B	B	B	B	B	B	B	B	B	B	B	B	B	B	41

337	H	H	H	H	H	H	H	H	H	H	H	H	H	H	H	43
338	A	A	A	A	A	A	A	A	A	A	A	A	A	A	A	38
339	H	H	H	H	H	H	H	H	H	H	H	H	H	H	H	41
340	B	B	B	B	B	B	B	B	B	B	B	B	B	B	B	41
341	H	H	H	H	H	H	H	H	H	H	H	H	H	H	H	44
342	A	A	A	A	A	A	A	A	A	A	A	A	A	A	A	37
343	H	H	H	H	H	H	H	H	H	H	H	H	H	H	H	52
344	H	H	H	H	H	H	H	H	H	H	H	H	H	H	H	46
345	A	A	A	A	A	A	H	H	H	H	H	H	H	H	H	40
346	A	H	H	H	H	H	A	A	A	A	A	A	A	A	A	39
347	A	A	A	A	A	A	H	H	H	H	H	H	H	H	H	40
348	H	H	H	H	H	H	A	A	A	A	A	A	A	A	A	36
349	H	H	H	H	H	H	H	H	H	H	H	H	H	H	H	58
350	H	H	H	H	H	H	H	H	H	H	H	H	H	H	H	50
351	H	H	H	H	H	H	H	H	H	H	H	H	H	H	H	47
352	A	A	A	A	A	A	A	A	A	A	A	A	A	A	A	39
353	H	H	H	H	H	H	H	H	H	H	H	H	H	H	H	41
354	A	A	A	A	A	A	A	A	A	A	A	A	A	A	A	38
355	H	H	H	H	H	H	H	H	H	H	H	H	H	H	H	61
356	B	B	B	B	B	B	B	B	B	B	B	B	B	B	B	43
357	A	A	A	A	H	H	H	H	H	H	H	H	A	A	A	39
358	H	H	H	B	B	B	B	B	B	B	B	B	B	B	B	50
359	H	H	H	H	H	H	H	H	H	H	H	H	H	H	H	44
360	H	H	H	H	H	H	H	H	H	H	H	H	H	H	H	42
361	A	A	A	A	A	A	A	A	A	A	A	A	A	A	A	38
362	A	A	A	A	A	A	A	A	A	A	A	A	A	A	A	35
363	H	H	H	H	H	H	H	H	H	H	H	H	H	H	H	43
364	A	A	A	A	A	A	H	H	H	H	H	H	H	H	H	39
365	A	A	A	A	A	A	H	H	H	H	H	H	H	H	H	55
366	B	B	B	B	B	B	B	B	B	B	B	B	B	B	B	44
367	A	A	A	A	A	A	A	A	A	A	A	A	A	A	A	41
368	H	H	H	H	H	H	B	B	B	B	B	B	B	B	B	43
369	H	H	H	H	H	H	A	A	A	A	A	A	A	A	A	48
370	A	A	A	B	A	A	A	A	A	A	A	A	A	A	A	42
371	B	B	B	B	B	B	B	B	B	B	B	B	B	B	B	56
372	H	H	H	H	H	H	H	H	H	H	H	H	H	H	H	49
373	H	H	H	H	H	H	H	H	H	H	H	H	H	H	H	44
374	H	H	H	H	H	H	A	A	A	A	A	A	A	A	A	40
375	A	A	A	A	A	A	A	A	A	A	A	A	A	A	A	39
376	H	H	H	H	H	H	H	H	H	H	H	H	H	H	H	48
377	B	B	B	B	B	B	B	B	B	B	B	B	B	B	B	51
378	H	H	H	H	H	H	H	H	H	H	H	H	H	H	H	47
379	H	H	H	H	H	H	A	A	A	A	A	A	A	A	A	46
380	H	H	H	H	H	H	H	H	H	H	H	H	H	H	H	59
381	H	H	H	H	H	H	H	H	H	H	H	H	H	H	H	42
382	H	H	H	H	H	H	H	H	H	H	H	H	H	H	H	44
383	H	H	H	H	H	H	H	H	H	H	H	H	H	H	H	42
384	H	H	H	H	H	H	H	H	H	H	H	H	H	H	H	45
385	H	B	B	B	B	B	B	B	B	B	B	B	B	B	B	50

386	H	H	H	H	H	H	H	H	H	H	H	H	H	H	H	40
387	H	B	B	B	B	B	H	H	H	H	H	H	H	H	H	47
388	H	H	H	H	H	H	H	H	H	H	H	H	H	H	H	41
389	H	H	H	H	H	H	H	H	H	H	H	H	H	H	H	37
390	A	A	A	A	A	A	A	A	A	A	A	A	A	A	A	42
391	H	B	B	B	B	B	B	B	B	B	B	B	B	B	B	45
392	H	H	H	H	H	H	H	H	H	H	H	H	H	H	H	44
393	H	H	H	H	H	H	B	H	H	H	H	H	H	H	H	41
394	A	A	A	A	A	A	A	A	A	A	A	A	A	A	A	41
395	H	H	H	H	H	H	H	H	H	H	H	H	H	H	H	48
396	H	H	H	H	H	H	A	A	A	A	A	A	A	A	A	41
397	H	H	H	H	H	H	H	H	H	H	H	H	H	H	H	45
398	H	H	H	H	H	H	A	A	A	A	A	A	A	A	A	47
399	H	H	H	H	H	H	H	H	H	H	H	H	H	H	H	56
400	H	H	H	H	H	H	H	H	H	H	H	H	H	H	H	48
401	B	B	B	B	B	B	B	B	B	B	B	B	B	B	B	53
402	H	B	B	B	B	B	B	B	B	B	B	B	B	B	B	48
403	H	B	B	B	B	B	B	B	B	B	B	B	B	B	B	46
404	H	H	H	H	H	H	H	H	H	H	H	H	H	H	H	47
405	H	H	H	H	H	H	H	H	H	H	H	H	H	H	H	39
406	H	H	H	H	H	H	H	H	H	H	H	H	H	H	H	45
407	A	A	A	A	A	A	A	A	A	A	A	A	A	A	A	41
408	H	H	H	H	H	H	H	H	H	H	H	H	H	H	H	39
409	B	B	B	B	B	B	B	B	B	B	B	B	B	B	B	44
410	A	A	A	A	A	A	A	A	A	A	A	A	A	A	A	33
411	H	H	H	H	H	H	H	H	H	H	H	H	H	H	H	43
412	H	H	H	H	H	H	H	H	H	H	H	H	H	H	H	38
413	A	A	A	A	A	A	H	H	H	H	H	H	H	H	H	37
414	H	H	H	H	H	H	H	H	H	H	H	H	H	H	H	47
415	A	A	A	A	A	A	A	A	A	A	A	A	A	A	A	47
416	H	B	B	B	B	B	B	B	B	H	H	B	B	B	B	45
417	H	H	H	H	H	H	H	H	H	H	H	H	H	H	H	48
418	A	A	A	A	A	A	H	H	H	H	H	H	H	H	H	43
419	A	A	A	A	A	A	A	A	A	A	A	A	A	A	A	42
420	B	B	B	B	B	B	B	B	B	B	B	B	B	B	B	41
421	H	H	H	H	H	H	H	H	H	H	H	H	H	H	H	45
422	H	H	H	H	H	H	H	H	H	H	H	H	H	H	H	47
423	H	B	B	B	B	B	B	B	B	B	B	B	B	B	B	44
424	A	A	A	A	A	A	A	A	A	A	A	A	A	A	A	44
425	B	B	B	B	B	B	B	B	B	B	B	B	B	B	B	47
426	H	H	H	H	H	H	H	H	H	H	H	H	H	H	H	52
427	H	H	H	H	H	H	A	A	A	A	A	A	A	A	A	48
428	A	A	A	A	A	A	H	H	H	H	H	H	H	H	H	39
429	B	B	B	B	B	B	B	B	B	B	B	B	B	B	B	44
430	A	A	A	A	A	A	A	A	A	A	A	A	A	A	A	39
431	A	A	A	A	A	A	H	H	H	H	H	H	H	H	H	40
432	H	H	H	H	H	H	H	H	H	H	H	H	H	H	H	45
433	A	A	A	A	A	A	A	A	A	A	A	A	A	A	A	37
434	H	H	H	H	H	H	H	H	H	H	H	H	H	H	H	48

435	H	H	H	H	H	H	A	A	A	A	A	A	A	A	A	46
436	H	B	B	B	B	B	B	B	B	B	B	B	B	B	B	41
437	B	B	B	B	B	B	B	B	B	B	B	B	B	B	B	57
438	H	H	H	H	H	H	H	H	H	H	H	H	H	H	H	41
439	H	H	H	H	H	H	H	H	H	H	H	H	H	H	H	45
440	H	H	H	H	H	H	H	H	H	H	H	H	H	H	H	37
441	H	H	H	H	H	H	H	H	H	H	H	H	H	H	H	43
442	B	B	B	B	H	H	B	B	B	B	B	B	B	B	B	45
443	H	H	H	H	H	H	H	H	H	H	H	H	H	H	H	56
444	H	H	H	H	H	H	H	H	H	H	H	H	H	H	H	43
445	H	H	H	H	H	H	A	A	A	A	A	A	A	A	A	46
446	H	H	H	H	H	H	H	H	H	H	H	H	H	H	H	48
447	H	H	H	H	H	H	H	H	H	H	H	H	H	H	H	37
448	B	B	B	B	B	B	B	B	B	B	B	B	B	B	B	55
449	A	A	A	A	A	A	A	A	A	A	A	A	A	A	A	37
450	B	B	B	B	B	B	B	B	B	B	B	B	B	B	B	44
451	H	H	H	H	H	H	H	H	H	H	H	H	H	H	H	46
452	H	H	H	A	A	A	H	H	H	H	H	H	H	H	H	46
453	A	A	A	A	A	A	A	A	A	A	A	A	A	A	A	41
454	A	A	A	A	A	A	A	A	A	A	A	A	A	A	A	41
455	A	A	A	A	A	A	H	H	H	H	H	H	H	H	H	41
456	A	A	A	A	A	A	A	A	A	A	A	A	A	A	A	42
457	A	A	A	A	A	A	A	A	A	A	A	A	A	A	A	37
458	H	H	H	H	H	H	H	H	H	H	H	H	H	H	H	46
459	A	A	A	A	A	A	A	A	A	A	A	A	A	A	A	40
460	H	H	H	H	H	H	H	H	H	H	H	H	H	H	H	41
461	H	H	H	H	H	H	H	H	H	H	H	H	H	H	H	53
462	H	H	H	H	H	H	H	H	H	H	H	H	H	H	H	45
463	A	A	A	A	A	A	A	A	A	A	A	A	A	A	A	41
464	A	A	A	A	A	A	A	A	A	A	A	A	A	A	A	39
465	H	H	H	H	H	H	H	H	H	H	H	H	H	H	H	51
466	A	A	A	A	A	A	A	A	A	A	A	A	A	A	A	41
467	H	H	H	H	H	H	H	H	H	H	H	H	H	H	H	51
468	H	H	H	H	H	H	H	H	H	H	H	H	H	H	H	47
469	H	H	H	A	A	A	H	H	H	H	H	H	H	H	H	49
470	B	B	B	B	B	B	B	B	B	B	B	B	B	B	B	51
471	B	B	B	B	B	B	B	B	B	B	B	B	B	B	B	44
472	A	A	A	A	A	A	A	A	A	A	A	A	A	A	A	39
473	A	A	A	A	A	A	A	A	A	A	A	A	A	A	A	39
474	H	H	H	H	H	H	A	A	A	A	A	A	A	A	A	55
475	B	B	B	B	B	B	B	B	B	B	B	B	B	B	B	75
476	H	H	H	H	H	H	H	H	H	H	H	H	H	H	H	55
477	H	H	H	H	H	H	H	H	H	H	H	H	H	H	H	61
478	A	A	A	A	A	A	A	A	A	A	A	A	A	A	A	39
479	B	B	B	B	B	B	B	B	B	B	B	B	B	B	B	55
480	H	H	H	H	H	H	H	H	H	H	H	H	H	H	H	50
481	B	B	B	B	B	B	B	B	B	B	B	B	B	B	B	55
482	A	A	A	A	A	A	A	A	A	A	A	A	A	A	A	41
483	B	B	B	B	B	B	B	B	B	B	B	B	B	B	B	49

484	H	H	H	H	H	H	H	H	H	H	H	H	H	H	H	54
485	H	H	H	H	H	H	H	H	H	H	H	H	H	H	H	48
486	A	A	A	A	A	A	A	A	A	A	A	A	A	A	A	39
487	A	A	A	A	A	A	A	A	A	A	A	A	A	A	A	41
488	A	A	A	A	A	A	A	A	A	A	A	A	A	A	A	44
489	H	H	H	H	H	H	H	H	H	H	H	H	H	H	H	53
490	B	B	B	B	B	B	B	B	B	B	B	B	B	B	B	50
491	A	A	A	A	A	A	A	A	A	A	A	A	A	A	A	49
492	H	H	H	H	H	H	H	H	H	H	H	H	H	H	H	45
493	A	A	A	A	A	A	A	A	A	A	A	A	A	A	A	57
494	H	H	H	H	H	H	H	H	H	H	H	H	H	H	H	55
495	B	B	B	B	B	B	B	B	B	B	B	B	B	B	B	48
496	H	H	H	H	H	H	H	H	H	H	H	H	H	H	H	46
497	H	H	H	H	H	H	H	H	H	H	H	H	H	H	H	39
498	H	H	H	H	H	H	H	H	H	H	H	H	H	H	H	48
499	H	H	H	H	H	H	H	H	H	H	H	H	H	H	H	42
500	A	A	A	A	A	A	A	A	A	A	A	A	A	A	A	41
501	A	A	A	A	A	A	A	A	A	A	A	A	A	A	A	45
502	A	A	A	A	A	A	A	A	A	A	A	A	A	A	A	44
503	B	B	B	B	B	B	B	B	B	B	B	B	B	B	B	72
504	A	A	A	A	A	A	A	A	A	A	A	A	A	A	A	44
505	A	A	A	A	A	A	H	H	H	H	H	H	H	H	H	37
506	H	H	H	H	H	H	H	H	H	H	H	H	H	H	H	47
507	A	A	A	A	A	A	A	A	A	A	A	A	A	A	A	46
508	B	B	B	B	B	B	B	B	B	B	B	B	B	B	B	53
509	H	H	H	H	H	H	A	A	A	A	A	A	A	A	A	60
510	H	H	H	H	H	H	H	H	H	H	H	H	H	H	H	45
511	B	B	B	B	B	B	B	B	B	B	B	B	B	B	B	43
512	H	H	H	H	H	H	A	A	A	A	A	A	A	A	A	47
513	A	A	A	A	A	A	A	A	A	A	A	A	A	A	A	42
514	H	H	H	H	H	H	H	H	H	H	H	H	H	H	H	52
515	B	B	B	B	B	B	B	B	B	B	B	B	B	B	B	49
516	B	B	B	B	B	B	B	B	B	B	B	B	B	B	B	58
517	H	H	H	H	H	H	H	H	H	H	H	H	H	H	H	47
518	A	A	A	A	A	A	A	A	A	A	A	A	A	A	A	42
519	B	B	B	B	B	B	B	B	B	B	B	B	B	B	B	47
520	H	H	H	H	H	H	H	H	H	H	H	H	H	H	H	41
521	A	A	A	A	A	A	A	A	A	A	A	A	A	A	A	42
522	A	A	A	A	A	A	A	A	A	A	A	A	A	A	A	44
523	H	H	H	H	H	H	H	H	H	H	H	H	H	H	H	44
524	A	A	A	A	A	A	B	H	H	H	H	H	H	H	H	47
525	A	A	A	A	A	A	H	H	H	H	H	H	H	H	H	46
526	H	H	H	H	H	H	H	H	H	H	H	H	H	H	H	50
527	A	A	A	A	A	A	A	A	A	A	A	A	A	A	A	39
528	H	H	H	H	H	H	H	H	H	H	H	H	H	H	H	49
529	H	H	H	H	H	H	H	H	H	H	H	H	H	H	H	46
530	B	B	B	B	B	B	B	B	B	B	B	B	B	B	B	51
531	B	B	B	B	B	B	B	B	B	B	B	B	B	B	B	45
532	A	A	A	A	A	A	A	A	A	A	A	A	A	A	A	37

533	A	A	A	A	A	A	A	A	A	A	A	A	A	A	A	42
534	B	B	B	B	B	B	B	B	B	B	B	B	B	B	B	40
535	H	H	H	H	H	H	H	H	H	H	H	H	H	H	H	53
536	H	H	H	H	H	H	A	A	A	A	A	A	A	A	A	37
537	A	A	A	A	A	A	A	A	A	A	A	A	A	A	A	37
538	A	A	A	A	A	A	A	A	A	A	A	A	A	A	A	41
539	A	A	A	A	A	A	A	A	A	A	A	A	A	A	A	52
540	A	A	A	A	A	A	A	A	A	A	A	A	A	A	A	39
541	B	B	B	B	B	B	B	B	B	B	B	B	B	B	B	56
542	H	H	H	H	H	H	H	H	H	H	H	H	H	H	H	54
543	H	H	H	H	H	H	H	H	H	H	H	H	H	H	H	42
544	B	B	B	B	B	B	B	B	B	B	B	B	B	B	B	47
545	H	H	H	H	H	H	H	H	H	H	H	H	H	H	H	54
546	H	H	H	H	H	H	H	H	H	H	H	H	H	H	H	40
547	H	B	B	B	B	B	B	B	B	B	B	B	B	B	B	51
548	H	B	B	B	B	B	B	B	B	B	B	B	B	B	B	50
549	H	H	H	H	H	H	H	H	H	H	H	H	H	H	H	44
550	B	B	B	B	B	B	B	B	B	B	B	B	B	B	B	47
551	H	H	H	H	H	H	H	H	H	H	H	H	H	H	H	43
552	B	B	B	B	B	B	B	B	B	B	B	B	B	B	B	61
553	A	A	A	A	A	A	A	A	A	A	A	A	A	A	A	43
554	H	H	H	H	H	H	H	H	H	H	H	H	H	H	H	44
555	A	A	A	A	A	A	A	A	A	A	A	A	A	A	A	53
556	A	A	A	A	A	A	A	A	A	A	A	A	A	A	A	41
557	A	A	A	A	A	A	A	A	A	A	A	A	A	A	A	42
558	B	B	B	B	B	B	B	B	B	B	B	B	A	A	A	46
559	H	H	H	H	H	H	H	H	H	H	H	H	H	H	H	50
560	B	B	B	B	B	B	B	B	B	B	B	B	B	B	B	56
561	H	H	H	H	H	H	H	H	H	H	H	H	H	H	H	43
562	B	B	B	B	B	B	B	B	B	B	B	B	B	B	B	51
563	H	H	H	H	H	H	H	H	H	H	H	H	H	H	H	45
564	H	H	H	H	H	H	H	H	H	H	H	H	H	H	H	47
565	H	H	H	H	H	H	H	H	H	H	H	H	H	H	H	58
566	H	H	H	H	H	H	H	H	H	H	H	H	H	H	H	50
567	H	H	H	H	H	H	H	H	H	H	H	H	H	H	H	37
568	A	A	A	A	A	A	A	A	A	A	A	A	A	A	A	35
569	B	B	B	B	B	B	B	H	H	H	H	H	H	H	H	49
570	H	H	H	H	H	H	H	H	H	H	H	H	H	H	H	52
571	B	B	B	B	B	B	B	H	H	H	H	H	H	H	H	47
572	B	B	B	B	B	B	B	B	B	B	B	B	B	B	B	60
573	A	A	A	A	A	A	A	A	A	A	A	A	A	A	A	46
574	B	B	B	B	B	B	B	B	B	B	B	B	B	B	B	68
575	H	H	H	H	H	H	A	H	H	H	H	A	A	A	A	50

Supplemental Table 3 Marker genotypes and days to first flowering of $F_{2:3}$ plants of the cross Kamphaeng Saen 2 (KS2) × ACC41 used for QTL analysis. A, B and H present genotype of Kamphaeng Saen 2 (KS2), ACC41 and hybrid (heterozygous), respectively. M1, M2, M3, M4, M5, M6 M7, M8, M9, M10, M11, M12, M13, M14 and M15 represent markers VrE1-SSR5, VrE1-SSR10, VrE1-SSR11, VrE1-SSR2, Vr05-ID19, VrE1-SSR21, VrDf-SSR5, VrDf-SSR47, VrDf-SSR45, Vr03-ID26, VrDf-SSR43, Vr05-ID31, VrDf-SSR39, VrDf-SSR17 and VrDf-SSR19, respectively.

$F_{2:3}$ individual	Marker															Days to first flowering
	M1	M2	M3	M4	M5	M6	M7	M8	M9	M10	M11	M12	M13	M14	M15	
1	H	H	H	H	H	H	H	H	H	H	H	H	H	H	H	62
2	A	A	A	A	A	A	A	A	A	A	A	A	A	A	A	38
3	H	H	H	H	H	H	H	H	H	H	H	H	H	H	H	50
4	H	H	H	H	H	H	H	H	H	H	H	H	H	H	H	65
5	H	H	H	H	H	H	H	H	H	H	H	H	H	H	H	41
6	B	B	B	B	B	B	B	B	B	B	B	B	B	B	B	58
7	A	A	A	A	A	A	A	A	A	A	A	A	A	A	A	54
8	H	H	H	H	H	H	H	H	H	H	H	H	H	H	H	50
9	A	A	A	A	A	A	B	B	B	B	B	B	B	B	B	44
10	B	B	B	B	B	B	B	B	B	B	B	B	B	B	B	56
11	B	B	B	B	B	B	B	B	B	B	B	B	B	B	B	53
12	H	H	H	H	H	H	B	B	B	B	B	B	B	B	B	51
13	H	H	H	H	H	H	B	B	B	B	B	B	B	B	B	52
14	H	H	H	H	H	H	B	B	B	B	B	B	B	B	B	50
15	H	H	H	H	H	H	B	B	B	B	B	B	B	B	B	53
16	A	A	A	A	A	A	B	B	B	B	B	B	B	B	B	45
17	H	H	H	H	H	H	B	B	B	B	B	B	B	B	B	49
18	B	B	B	B	B	B	B	B	B	B	B	B	B	B	B	53
19	H	H	H	H	H	H	B	B	B	B	B	B	B	B	B	44
20	H	H	H	H	H	H	B	B	B	B	B	B	B	B	B	45
21	A	A	A	A	A	A	B	B	B	B	B	B	B	B	B	52
22	H	H	H	H	H	H	B	B	B	B	B	B	B	B	B	48
23	B	B	B	B	B	B	B	B	B	B	B	B	B	B	B	57
24	H	H	H	H	H	H	B	B	B	B	B	B	B	B	B	54
25	A	A	A	A	A	A	B	B	B	B	B	B	B	B	B	42
26	H	H	H	H	H	H	B	B	B	B	B	B	B	B	B	48
27	H	H	H	H	H	H	B	B	B	B	B	B	B	B	B	48
28	B	B	B	B	B	B	B	B	B	B	B	B	B	B	B	54
29	B	B	B	B	B	B	B	B	B	B	B	B	B	B	B	56
30	H	H	H	H	H	H	B	B	B	B	B	B	B	B	B	58
31	H	H	H	H	H	H	B	B	B	B	B	B	B	B	B	54
32	H	H	H	H	H	H	B	B	B	B	B	B	B	B	B	49
33	A	A	A	A	A	A	B	B	B	B	B	B	B	B	B	44
34	B	B	B	B	B	B	A	A	A	A	A	A	A	A	A	46
35	B	B	B	B	B	B	A	A	A	A	A	A	A	A	A	45
36	H	H	H	H	H	H	A	A	A	A	A	A	A	A	A	46
37	B	B	B	B	B	B	A	A	A	A	A	A	A	A	A	51
38	H	H	H	H	H	H	A	A	A	A	A	A	A	A	A	44
39	A	A	A	A	A	A	A	A	A	A	A	A	A	A	A	40
40	H	H	H	H	H	H	A	A	A	A	A	A	A	A	A	64
41	A	A	A	A	A	A	A	A	A	A	A	A	A	A	A	44
42	A	A	A	A	A	A	A	A	A	A	A	A	A	A	A	42

43	B	B	B	B	B	B	A	A	A	A	A	A	A	A	A	53
44	A	A	A	A	A	A	A	A	A	A	A	A	A	A	A	56
45	A	A	A	A	A	A	A	A	A	A	A	A	A	A	A	37
46	H	H	H	H	H	H	A	A	A	A	A	A	A	A	A	42
47	B	B	B	B	B	B	A	A	A	A	A	A	A	A	A	55
48	B	B	B	B	B	B	A	A	A	A	A	A	A	A	A	54
49	A	A	A	A	A	A	A	A	A	A	A	A	A	A	A	48
50	B	B	B	B	B	B	A	A	A	A	A	A	A	A	A	56
51	H	H	H	H	H	H	A	A	A	A	A	A	A	A	A	57
52	A	A	A	A	A	A	A	A	A	A	A	A	A	A	A	30
53	B	B	B	B	B	B	A	A	A	A	A	A	A	A	A	63
54	A	A	A	A	A	A	A	A	A	A	A	A	A	A	A	45
55	H	H	H	H	H	H	A	A	A	A	A	A	A	A	A	52
56	B	B	B	B	B	B	A	A	A	A	A	A	A	A	A	46
57	H	H	H	H	H	H	A	A	A	A	A	A	A	A	A	50
58	H	H	H	H	H	H	A	A	A	A	A	A	A	A	A	48
59	H	H	H	H	H	H	A	A	A	A	A	A	A	A	A	43
60	H	H	H	H	H	H	A	A	A	A	A	A	A	A	A	42
61	B	B	B	B	B	B	A	A	A	A	A	A	A	A	A	46
62	H	H	H	H	H	H	A	A	A	A	A	A	A	A	A	56
63	H	H	H	H	H	H	A	A	A	A	A	A	A	A	A	62
64	A	A	A	A	A	A	A	A	A	A	A	A	A	A	A	43
65	B	B	B	B	B	B	A	A	A	A	A	A	A	A	A	53
66	H	H	H	H	H	H	A	A	A	A	A	A	A	A	A	58
67	H	H	H	H	H	H	A	A	A	A	A	A	A	A	A	58
68	B	B	B	B	B	B	A	A	A	A	A	A	A	A	A	49
69	H	H	H	H	H	H	A	A	A	A	A	A	A	A	A	58
70	A	A	A	A	A	A	A	A	A	A	A	A	A	A	A	42
71	B	B	B	B	B	B	A	A	A	A	A	A	A	A	A	51
72	H	H	H	H	H	H	A	A	A	A	A	A	A	A	A	51
73	H	H	H	H	H	H	A	A	A	A	A	A	A	A	A	48
74	B	B	B	B	B	B	A	A	A	A	A	A	A	A	A	51
75	A	A	A	A	A	A	A	A	A	A	A	A	A	A	A	40
76	H	H	H	H	H	H	A	A	A	A	A	A	A	A	A	51
77	B	B	B	B	B	B	A	A	A	A	A	A	A	A	A	40
78	A	A	A	A	A	A	A	A	A	A	A	A	A	A	A	46
79	B	B	B	B	B	B	A	A	A	A	A	A	A	A	A	45
80	H	H	H	H	H	H	A	A	A	A	A	A	A	A	A	45
81	H	H	H	H	H	H	A	A	A	A	A	A	A	A	A	46
82	B	B	B	B	B	B	H	H	H	H	H	H	H	H	H	45
83	H	H	H	H	H	H	A	A	A	A	A	A	A	A	A	44
84	B	B	B	B	B	B	A	A	A	A	A	A	A	A	A	62
85	A	A	A	A	A	A	A	A	A	A	A	A	A	A	A	58
86	H	H	H	H	H	H	A	A	A	A	A	A	A	A	A	54
87	A	A	A	A	A	A	A	A	A	A	A	A	A	A	A	42
88	H	H	H	H	H	H	A	A	A	A	A	A	A	A	A	51
89	H	H	H	H	H	H	A	A	A	A	A	A	A	A	A	48
90	H	H	H	H	H	H	A	A	A	A	A	A	A	A	A	53
91	B	B	B	B	B	B	A	A	A	A	A	A	A	A	A	50

92	B	B	B	B	B	B	A	A	A	A	A	A	A	A	A	56
93	H	H	H	H	H	H	A	A	A	A	A	A	A	A	A	45
94	B	B	B	B	B	B	A	A	A	A	A	A	A	A	A	48
95	H	B	B	B	B	B	B	B	B	B	B	B	B	B	B	56
96	H	B	B	B	B	B	B	B	B	B	B	B	B	B	B	52
97	H	B	B	B	B	B	B	B	B	B	B	B	B	B	B	62
98	H	B	B	B	B	B	B	B	B	B	B	B	B	B	B	48
99	H	B	B	B	B	B	B	B	B	B	B	B	B	B	B	67
100	B	B	B	B	B	B	B	B	B	B	B	B	B	B	B	49
101	H	B	B	B	B	B	B	B	B	B	B	B	B	B	B	68
102	B	B	B	B	B	B	B	B	B	B	B	B	B	B	B	73
103	H	B	B	B	B	B	B	B	B	B	B	B	B	B	B	61
104	B	B	B	B	B	B	B	B	B	B	B	B	B	B	B	53
105	H	B	B	B	B	B	B	B	B	B	B	B	B	B	B	52
106	H	B	B	B	B	B	B	B	B	B	B	B	B	B	B	73
107	H	B	B	B	B	B	B	B	B	B	B	B	B	B	B	56
108	H	B	B	B	B	B	B	B	B	B	B	B	B	B	B	58
109	H	B	B	B	B	B	B	B	B	B	B	B	B	B	B	69
110	H	H	H	H	H	H	A	A	A	A	A	A	A	A	A	74
111	H	B	B	B	B	B	A	A	A	A	A	A	A	A	A	65
112	H	H	H	H	H	H	A	A	A	A	A	A	A	A	A	49
113	A	A	A	A	A	A	A	A	A	A	A	A	A	A	A	41
114	A	A	A	A	A	A	A	A	A	A	A	A	A	A	A	50
115	A	A	A	A	A	A	A	A	A	A	A	A	A	A	A	53
116	A	A	A	A	A	A	A	A	A	A	A	A	A	A	A	49
117	A	A	A	A	A	A	A	A	A	A	A	A	A	A	A	48
118	A	A	A	A	A	A	A	A	A	A	A	A	A	A	A	65
119	H	H	H	H	H	H	A	A	A	A	A	A	A	A	A	69
120	H	H	H	H	H	H	A	A	A	A	A	A	A	A	A	69
121	H	B	B	B	B	B	A	A	A	A	A	A	A	A	A	64
122	H	B	B	B	B	B	A	A	A	A	A	A	A	A	A	68
123	A	A	A	A	A	A	A	A	A	A	A	A	A	A	A	69
124	B	B	B	B	B	B	A	A	A	A	A	A	A	A	A	62
125	H	H	H	H	H	H	A	A	A	A	A	A	A	A	A	53
126	H	H	H	H	H	H	A	A	A	A	A	A	A	A	A	59
127	A	A	A	A	A	A	A	A	A	A	A	A	A	A	A	30
128	H	H	H	H	H	H	A	A	A	A	A	A	A	A	A	49
129	H	H	H	H	H	H	A	A	A	A	A	A	A	A	A	53
130	H	B	B	B	B	B	A	A	A	A	A	A	A	A	A	71
131	H	B	B	B	B	B	A	A	A	A	A	A	A	A	A	50
132	H	H	H	H	H	H	A	A	A	A	A	A	A	A	A	56
133	H	H	H	H	H	H	A	A	A	A	A	A	A	A	A	66
134	H	H	H	H	H	H	A	A	A	A	A	A	A	A	A	59
135	H	B	B	B	B	B	A	A	A	A	A	A	A	A	A	65
136	H	B	B	B	B	B	A	A	A	A	A	A	A	A	A	71
137	A	A	A	A	A	A	A	A	A	A	A	A	A	A	A	49
138	B	B	B	A	A	A	A	A	A	A	A	A	A	A	A	52
139	B	B	B	B	B	B	A	A	A	A	A	A	A	A	A	67
140	B	B	B	H	H	H	A	A	A	A	A	A	A	A	A	56

141	B	B	B	B	B	B	A	A	A	A	A	A	A	A	A	70
142	A	A	A	A	A	A	A	A	A	A	A	A	A	A	A	46
143	B	B	B	B	B	B	A	A	A	A	A	A	A	A	A	66
144	H	H	H	H	H	H	A	A	A	A	A	A	A	A	A	66
145	A	A	A	A	A	A	A	A	A	A	A	A	A	A	A	45
146	H	H	H	H	H	H	A	A	A	A	A	A	A	A	A	69
147	H	H	H	H	H	H	A	A	A	A	A	A	A	A	A	56
148	H	B	B	B	B	B	A	A	A	A	A	A	A	A	A	50
149	A	A	A	A	A	A	A	A	A	A	A	A	A	A	A	66
150	H	H	H	H	H	H	A	A	A	A	A	A	A	A	A	62
151	A	A	A	A	A	A	A	A	A	A	A	A	A	A	A	43
152	A	A	A	A	A	A	A	A	A	A	A	A	A	A	A	48
153	A	A	A	A	A	A	A	A	A	A	A	A	A	A	A	46
154	A	A	A	A	A	A	A	A	A	A	A	A	A	A	A	47
155	A	A	A	A	A	A	A	A	A	A	A	A	A	A	A	67
156	A	A	A	A	A	A	A	A	A	A	A	A	A	A	A	48
157	A	A	A	A	A	A	A	A	A	A	A	A	A	A	A	48
158	A	A	A	A	A	A	A	A	A	A	A	A	A	A	A	44
159	A	A	A	A	A	A	A	A	A	A	A	A	A	A	A	54
160	A	A	A	A	A	A	A	A	A	A	A	A	A	A	A	55
161	H	H	H	H	H	H	A	A	A	A	A	A	A	A	A	65
162	H	H	H	H	H	H	A	A	A	A	A	A	A	A	A	45
163	H	H	H	H	H	H	A	A	A	A	A	A	A	A	A	51
164	H	H	H	H	H	H	A	A	A	A	A	A	A	A	A	48
165	A	A	A	A	A	A	A	A	A	A	A	A	A	A	A	49
166	A	A	A	A	A	A	A	A	A	A	A	A	A	A	A	49
167	H	H	H	H	H	H	A	A	A	A	A	A	A	A	A	57
168	A	A	A	A	A	A	A	A	A	A	A	A	A	A	A	45
169	B	B	B	B	B	B	A	A	A	A	A	A	A	A	A	46
170	H	H	H	H	H	H	A	A	A	A	A	A	A	A	A	50
171	H	H	H	H	H	H	A	A	A	A	A	A	A	A	A	58
172	H	H	H	H	H	H	A	A	A	A	A	A	A	A	A	57
173	H	H	H	H	H	H	A	A	A	A	A	A	A	A	A	72
174	H	H	H	H	H	H	A	A	A	A	A	A	A	A	A	58
175	H	H	H	H	H	H	A	A	A	A	A	A	A	A	A	57
176	A	A	A	A	A	A	A	A	A	A	A	A	A	A	A	58
177	A	A	A	A	A	A	A	A	A	A	A	A	A	A	A	57
178	A	A	A	A	A	A	A	A	A	A	A	A	A	A	A	58
179	H	H	H	H	H	H	A	A	A	A	A	A	A	A	A	60
180	H	H	H	H	H	H	A	A	A	A	A	A	A	A	A	68
181	H	H	H	H	H	H	A	A	A	A	A	A	A	A	A	70
182	A	A	A	A	A	A	A	A	A	A	A	A	A	A	A	50
183	B	B	B	B	B	B	A	A	A	A	A	A	A	A	A	67
184	B	B	B	B	B	B	H	H	H	H	H	H	H	H	H	60
185	H	H	H	H	H	H	A	A	A	A	A	A	A	A	A	64
186	H	B	B	B	B	B	A	A	A	A	A	A	A	A	A	58
187	A	A	A	A	A	A	A	A	A	A	A	A	A	A	A	55
188	H	B	B	B	B	B	A	A	A	A	A	A	A	A	A	56
189	H	H	H	H	H	H	A	A	A	A	A	A	A	A	A	67

190	H	B	B	B	B	B	A	A	A	A	A	A	A	A	A	69
191	H	H	H	H	H	H	A	A	A	A	A	A	A	A	A	53
192	A	A	A	A	A	A	A	A	A	A	A	A	A	A	A	54
193	H	H	H	H	H	H	A	A	A	A	A	A	A	A	A	75
194	H	H	H	H	H	H	A	A	A	A	A	A	A	A	A	70
195	A	A	A	A	A	A	A	A	A	A	A	A	A	A	A	54
196	A	A	A	A	A	A	A	A	A	A	A	A	A	A	A	51
197	H	H	H	H	H	H	H	H	H	H	H	H	H	H	H	38
198	A	A	A	A	A	A	A	A	A	A	A	A	A	A	A	38
199	H	H	H	H	H	H	H	H	H	H	H	H	H	H	H	49
200	H	H	H	H	H	H	H	H	H	H	H	H	H	H	H	46
201	H	H	H	H	H	H	H	H	H	H	H	H	H	H	H	54
202	A	A	A	A	A	A	H	H	H	A	A	A	A	A	A	44
203	B	B	B	B	B	B	B	B	B	B	B	B	B	B	B	59
204	H	B	B	B	B	B	B	B	B	B	B	B	B	B	B	59
205	H	H	H	H	H	H	H	H	H	H	H	H	H	H	H	36
206	B	B	B	B	B	B	H	H	H	H	H	H	H	H	H	48
207	H	B	B	B	B	B	B	B	B	B	B	B	B	B	B	44
208	B	B	B	B	B	B	B	B	B	B	B	B	B	B	B	55
209	H	H	H	H	H	H	A	A	A	A	A	A	A	A	A	56
210	B	B	B	B	B	B	B	B	B	B	B	B	B	B	B	48
211	H	H	H	H	H	H	H	H	H	H	H	H	H	H	H	47
212	H	H	H	H	H	H	H	H	H	H	H	H	H	H	H	45
213	H	H	H	H	H	H	H	H	H	H	H	H	H	H	H	51
214	H	H	H	H	H	H	H	H	H	H	H	H	H	H	H	54
215	H	H	H	H	H	H	H	H	H	H	H	H	H	H	H	46
216	H	H	H	H	H	H	H	H	H	H	H	H	H	H	H	43
217	H	H	H	H	H	H	H	H	H	H	H	H	H	H	H	53

Supplemental Table 4 Details of phytochome E protein sequences used in phylogenetic analysis used in this study.

Code name	Protein sequence ID	Plant species	Sources
Vrad-phyE	EVM0010407.1	*Vigna radiata*	Yan et al. (2020)
Vang-phyE	Vigan.02G285600.01	*Vigna angularis*	https://viggs.dna.affrc.go.jp
Vmun-phyE	evm.TU.Scaffold_5184_HRSCAF=5881.795	*Vigna mungo*	https://www.nstda.or.th/noc/research-outputs/genome-assemblies
Vung-phyE	Vigun09g050600.1.p	*Vigna unguiculata*	https://phytozome-next.jgi.doe.gov
Pvul-phyE	Phvul.009G213400.1.p	*Phaseolus vulgaris*	https://phytozome-next.jgi.doe.gov
Plun-phyE	Pl09G0000305400.5	*Phaseolus lunatus*	https://phytozome-next.jgi.doe.gov
Lpur-phyE	Labpu09g003090.1	*Lablab purpureus*	https://hpc.ilri.cgiar.org
Gmax-phyE1	Glyma.09G088500.1.p	*Glycine max*	https://phytozome-next.jgi.doe.gov
Gmax-PhyE2	Glyma.15G196500.1.p	*Glycine max*	https://phytozome-next.jgi.doe.gov
Gsoj-PhyE1	GlysoPI483463.09G075300.1	*Glycine soja*	https://phytozome-next.jgi.doe.gov
Gsoj-PhyE2	GlysoPI483463.15G173700.1	*Glycine soja*	https://phytozome-next.jgi.doe.gov
Ahyp-PhyE1	arahy.Tifrunner.gnm2.ann1.PM8GQZ.1	*Arachis hypogaea*	https://phytozome-next.jgi.doe.gov
Ahyp-PhyE2	arahy.Tifrunner.gnm2.ann1.F3Y113.1	*Arachis hypogaea*	https://phytozome-next.jgi.doe.gov
Adur-PhyE	aradu.V14167.gnm1.ann1.Aradu.H9LWJ.1	*Arachis duraensis*	https://phytozome-next.jgi.doe.gov
Ccaj-PhyE	XP_020216614.1	*Cajanus cajan*	https://www.ncbi.nlm.nih.gov
Cari-phyE	Ca_22572	*Cicer arietinum*	https://phytozome-next.jgi.doe.gov
Mtru-phyE	Medtr2g049520.1	*Medicago truncatula*	https://phytozome-next.jgi.doe.gov
Ljap-phyE	Lj6g0024888.1	*Lotus japonicus*	https://phytozome-next.jgi.doe.gov
Tsub-phyE	trisu.Daliak.gnm2.ann1.Ts_00852.1	*Trifolium subterraneum*	https://legumeinfo.org

Supplemental Table 5 Genes locating in the 164.87-kb region on chromosome 4 of the mungbean cultivar Sulv1 containing the *qFld2.1* controlling days to first flowering.

**Gene**	**Location on chromosome 4**	**Annotation**
EVM0001354	38,944,024-38,948,741	NEDD8 ultimate buster 1
EVM0024695	38,951,039-38,955,036	Protein OS-9 homolog
EVM0025203	38,972,356-38,980,433	protein PHOX1
EVM0022174	38,981,443-38,989,574	Uncharacterized protein
EVM0013028	38,998,272-39,002,525	Heparanase-like protein 3
EVM0017649	39,003,755-39,011,899	Putative ALA-interacting subunit 2
EVM0000219	39,014,264-39,014,489	Uncharacterized protein
EVM0025629	39,017,808-39,018,285	Uncharacterized protein
EVM0020684	39,037,192-39,045,721	CBL-interacting serine/threonine-protein kinase 23
EVM0014328	39,049,376-39,050,595	Transcription termination factor MTEF1, chloroplastic
EVM0011832	39,051,328-39,055,632	Uncharacterized protein
EVM0009034	39,060,612-39,063,183	Putative pentatricopeptide repeat-containing protein
EVM0010407	39,069,723-39,074,829	Phytochrome E
EVM0027263	39,076,020-39,083,246	Protein MEI2-like 5
